# Supplementary material for: Woody species diversity, structure and community distribution along environmental gradients of Seqela Dry Afromontane forest in Northwestern Ethiopia
Source: PLoS One. 2025 Jan 17;20(1):e0313020. doi: 10.1371/journal.pone.0313020 (PMC11741620; doi:10.1371/journal.pone.0313020)
Supplement: S1 Appendix — (DOCX) [file pone.0313020.s001.docx]

S1 Appendix**.** List of woody plant species collected from Seqela forest Quarit District

| No | Scientific Name | Family | | Habit | Local name |
| --- | --- | --- | --- | --- | --- |
| 1 | *Vachellia abyssinica* Hochst. ex Benth. | Fabaceae | | Tree | Girar |
| 2 | *Acanthus sennii*Chiov. | Acanthaceae | | Shrub | Kosheshila |
| 3 | *Albizia gummifera*J. F. Gmel. C. A. Sm. | Fabaceae   \|  \|  \| \| --- \| --- \| | | tree | Sesa |
| 4 | *Allophylus abyssinicus*(Hochst.) Radlk   \|  \| \| --- \| | Sapindaceae   \|  \|  \| \| --- \| --- \| | | tree | Embs |
| 5 | *Apodytes dimidiata*E. Mey. ex Arn.   \|  \| \| --- \| | Icacinaceae | | Tree | Dong |
| 6 | *Asparagus africanus*Lam. | Asparagaceae | | Shrub | Yeset Kest |
| 7 | *Bersama abyssinica*Fresen. | Melianthaceae | | Shrub | Azamara |
| 8 | *Bridelia micrantha* (Hochst.) Baill. | Euphorbiaceae | | Shrub | Yenebir Tifir |
| 9 | *Brucea antidysenterica* J. F. Mill | Simaroubaceae | | Shrub | Abalo |
| 10 | *Buddleja davidii*Franch. | Loganiaceae | | Shrub | Tikur Anfar |
| 11 | *Buddleja polystachya*Fresen. | Loganiaceae | | Shrub | Nech anfar |
| 12 | *Calpurnia aurea* (Ait.) Benth. | Fabaceae | | Shrub | Ligita |
| 13 | *Capparis tomentosa*Lam.   \|  \| \| --- \| | Capparidaceae | | Shrub | Gomeru |
| 14 | *Carissa spinarum*L. | Apocynaceae | | Shrub | Agam |
| 15 | *Catha edulis* (Vahl) Forssk. ex Endl. | Celastraceae | | Shrub | Chat |
| 16 | *Clausena anisata*Willd.) Benth. | Rutaceae | | Shrub | Limich |
| 17 | *Clematis longicauda Steud. exA.Rich.* | Ranunculaceae | | Liana | Azo hareg |
| 18 | *Clutia abyssinica*Jaub. & Spach. | Euphorbiaceae | | Shrub | Fiyelfej |
| 19 | *Croton macrostachyus*Del. | Euphorbiaceae | | Tree | Bisana |
| 20 | *Discopodium penninervium* Hochst. | Solanaceae | | Tree | Aluma |
| *21* | *Dodonaea angustifolia* L. f | Sapindaceae | | Shrub | Kitkita |
| *22* | *Dombeya torrida* (J.F.Gmel.) P.Bamps | Sterculiaceae | | Tree | Wilkifa |
| *23* | *Dovyalis abyssinica* (A.Rich.) Warb.   \|  \| \| --- \| | Flacourtiaceae   \|  \| \| --- \| | | Tree   \|  \| \| --- \| | Koshim |
| *24* | *Dracaena steudneri Engl* | Dracaenaceae | | Tree | Marqoo |
| *25* | *Ekebergia capensis*Sparrm. | Meliaceae | | Tree | Lol |
| *26* | *Embelia schimperi*Vatke | Myrsinaceae | | Liana | Enkoko |
| *27* | *Erythrina brucei*Schweinf. | Fabaceae | | Tree | Korch |
| *28* | *Euphorbia abyssinica*Gmel. | Euphorbiaceae | | Tree | Kulkual |
| *29* | *Galiniera saxifraga*Hochst.) Bridson | Rubiaceae | | Tree | Yetotakolet |
| *30* | *Gnidia glauca*(Fresen.)Gilg | Thymelaeaceae | | Shrub | Awra |
| *31* | *Grewia ferruginea*Hochst. ex A. Rich. | Tiliaceae | | Tree | Lenquata   \|  \| \| --- \| |
| *32* | *Hagenia abyssinica*(Bruce) J.F. Gmel. | Rosaceae | | Tree | Koso |
| *33* | *Hypericum quartinianum*A. Rich. | Hypericaceae | | Shrub | Amija |
| *34* | *Jasminum abyssinicum* Hochst. ex DC. | Oleaceae | | Liana | Abita hareg |
| *35* | *Juniperus procera*L. | Cupressaceae | | Tree | Tsid |
| *36* | *Laggera tomentosa*Sch.-Bip. | Asteraceae | | Shrub | Gimane |
| 37 | *Maesa lanceolata*Forssk. | Myrsinaceae | | Shrub | Kilaba |
| 38 | *Maytenus addat* (Loes.) Sebsebe | Celastraceae | | Shrub | Qemer atat |
| *39* | *Maytenus obscura*(A.Rich.) Cuf. | Celastraceae | | Tree | Qoba |
| *40* | *Myrica salicifolia*A.Rich | Myricaceae | | Tree | Shi**n**et |
| *41* | *Myrsine africana*L. | Myrsinaceae | | Shrub | Kechemo |
| *42* | *Ocimum lamiifolium*Hochst. Ex Benth   \|  \| \| --- \| | Lamiaceae | | Shrub | Damakese   \|  \| \| --- \| |
| *43* | *Olea europaea*L. subsp. cuspidata | Oleaceae   \|  \| \| --- \| | | Tree | Woira |
| 44 | *Olinia rochetiana*A. Juss | Oliniaceae | | Tree | Tife |
| *45* | *Osyris quadripartita*Decn. | Santalaceae | | Shrub | Qeret |
| *46* | *Otostegia integrifolia*Benth | Lamiaceae | | Shrub | Tunjit |
| *47* | *Periploca linearifolia*Quart. -Dill. & A. Rich. | Asclepiadaceae | | Liana | Yemidir hareg   \|  \| \| --- \| |
| *48* | *Phytolacca dodecandra*L 'Herit. | Phytolaccaceae | | Liana | Endod |
| *49* | *Pittosporum viridiflorum* Sims. | Pittosporaceae | | Tree | Elaho |
| *50* | *Prunus Africana* (Hook. f.) Kalkm. | Rosaceae | | Tree | Koma |
| *51* | *Rhamnus prinoides* L’Herit. | Rhamnaceae | | Shrub | Gesho |
| *52* | *Rhus glutinosa* A. Rich. subsp. *glutinosa* | Anacardiaceae | | Shrub | Qamo |
| *53* | *Rhus vulgaris* Meikle | Anacardiaceae | | Tree | Ashkamo |
| *54* | *Rosa abyssinica* Lindley | Rosaceae | | Shrub | Qega |
| *55* | *Rubus steudneri* Schwienf. | Rosaceae | | Liana | Enjori |
| *56* | *Rumex nervosus* Vahl | Polygonaceae | | Shrub | Ambacho |
| *57* | *Rytigynia neglecta*(Hiern) Robyns | Rubiaceae | | Shrub | Dingayseber |
| *58* | *Schefflera abyssinica*(Hochst. ex A. Rich.) | Araliaceae | | Tree | Getem |
| *59* | *Solanecio gigas* (Vatke) C. Jeffrey | Asteraceae | | Shrub | Yeshkoko Gomen |
| *60* | *Urera hypselodendron*(A. Rich) Wedd. | Urticaceae | | Liana | Lankuso |
| *61* | *Vepris nobilis*Del.   \|  \| \| --- \| | Rutaceae | | Tree | Sila |
| *62* | *Vernonia amygdalina* Del. | Asteraceae | | Shrub | Girawa |
| 63 | *Vernonia myriantha*Hook.f. | Asteraceae | | Shrub | Dengorita |
| *64* | *Zehneria scabra* (Linn.f.) Sond. | Cucurbitaceae | | Liana | Areg Resa |
| 45 | *Cobretum molle*Fresen. | | Combretaceae | Shrub | Kolla abalo |
| 66 | *Ficus sur*Forssk. | | Moraceae | Tree | Shola |
| 67 | *Lippia adoensis*Hochst. ex Walp. | | Verbenaceae | Shrub | Koseret kesi |
| 68 | *Maytenus arbutifolia*(A.Rich.) Wilczek | | Myrsinaceae | Shrub | Atat |
