# Supplementary material for: Woody species diversity, structure and community distribution along environmental gradients of Seqela Dry Afromontane forest in Northwestern Ethiopia
Source: PLoS One. 2025 Jan 17;20(1):e0313020. doi: 10.1371/journal.pone.0313020 (PMC11741620; doi:10.1371/journal.pone.0313020)
Supplement: S2 Appendix — (DOCX) [file pone.0313020.s002.docx]

S2 Appendix. The values for diversity (H) and Evenness are as follows for each plots

| **Plot** | | **H** | **ShanEvenness** |
| --- | --- | --- | --- |
| 1 | 2.759219 | | 0.9370949 |
| 2 | 2.694292 | | 0.9321611 |
| 3 | 2.918543 | | 0.9586209 |
| 4 | 2.419885 | | 0.9169504 |
| 5 | 2.254201 | | 0.9071572 |
| 6 | 2.519761 | | 0.9304704 |
| 7 | 2.211341 | | 0.9222009 |
| 8 | 2.239357 | | 0.9338845 |
| 9 | 2.268098 | | 0.9458703 |
| 10 | 2.199336 | | 0.9171945 |
| 11 | 2.444807 | | 0.9531598 |
| 12 | 2.272374 | | 0.9144707 |
| 13 | 2.167163 | | 0.9411870 |
| 14 | 2.026561 | | 0.9223275 |
| 15 | 2.216568 | | 0.9626431 |
| 16 | 2.309600 | | 0.9294513 |
| 17 | 2.164832 | | 0.9028049 |
| 18 | 2.156616 | | 0.9366064 |
| 19 | 2.327455 | | 0.9366369 |
| 20 | 2.050761 | | 0.9333418 |
| 21 | 2.165885 | | 0.9406321 |
| 22 | 2.045304 | | 0.9308579 |
| 23 | 1.985851 | | 0.9549926 |
| 24 | 2.040652 | | 0.9287410 |
| 25 | 2.204958 | | 0.9195388 |
| 26 | 2.109869 | | 0.9163043 |
| 27 | 1.929624 | | 0.9279531 |
| 28 | 2.071103 | | 0.8994685 |
| 29 | 1.899832 | | 0.9136262 |
| 30 | 1.975314 | | 0.8990044 |
| 31 | 2.039341 | | 0.9281442 |
| 32 | 2.018559 | | 0.9186858 |
| 33 | 2.124350 | | 0.9225935 |
| 34 | 2.050423 | | 0.9331877 |
| 35 | 2.013421 | | 0.9682506 |
| 36 | 2.346634 | | 0.9786223 |
| 37 | 2.189995 | | 0.9511027 |
| 38 | 2.404082 | | 0.9674736 |
| 39 | 2.028287 | | 0.9231131 |
| 40 | 1.639156 | | 0.8423594 |
| 41 | 1.703073 | | 0.8752063 |
| 42 | 2.059033 | | 0.9371062 |
| 43 | 1.815478 | | 0.9329712 |
| 44 | 1.846135 | | 0.8878031 |
| 45 | 2.015226 | | 0.9171688 |
| 46 | 1.930769 | | 0.9285036 |
| 47 | 1.999449 | | 0.9099883 |
| 48 | 1.953450 | | 0.9394107 |
| 49 | 1.813219 | | 0.9318102 |
| 50 | 1.777306 | | 0.9133545 |
| 51 | 1.876093 | | 0.9022098 |
| 52 | 1.798776 | | 0.8650283 |
| **Total** | **2.12** | | **0.92** |
