# Supplementary material for: Woody species diversity, structure and community distribution along environmental gradients of Seqela Dry Afromontane forest in Northwestern Ethiopia
Source: PLoS One. 2025 Jan 17;20(1):e0313020. doi: 10.1371/journal.pone.0313020 (PMC11741620; doi:10.1371/journal.pone.0313020)
Supplement: Attachment — Submitted filename: PONE-D-24-05897_reviewed.pdf [file pone.0313020.s004.pdf]

PLOS ONE

Plant community distribution along environmental gradient of Seqela Dry Afromontane Forest in Northwestern Ethiopia

--Manuscript Draft--

**Manuscript Number:**	PONE-D-24-05897
**Article Type:**	Research Article
**Full Title:**	Plant community distribution along environmental gradient of Seqela Dry Afromontane Forest in Northwestern Ethiopia
**Short Title:**	Plant community distribution
**Corresponding Author:**	Liyew Birhanu<br>Debre Markos University College of Natural and Computational Science<br>Debremarkos, ETHIOPIA
**Keywords:**	Canonical Correspondence Analysis, Hierarchical cluster analysis, Seqela dry Afromontane forest, Plant community
**Abstract:**	Dry evergreen Afromontane forests are severely threatened due to the expansion of agriculture, overgrazing by livestock, and human population growth. The objective of this study was to investigate the floristic composition, vegetation structure, and classification of plant communities and assess the relationship between plant community types and environmental variables in the study forest. Systematic sampling was used to collect vegetation and environmental data from 52 (20 m x 20 m) (400 m2) plots established at every 200 m intervals along transect lines that were 100 m apart from each other. Density, DBH, basal area, frequency, and importance value indices (IVI) of woody species were computed to characterize the vegetation structure of the forest. Hierarchical cluster analysis and canonical Correspondence Analysis (CCA) with R software were used to identify plant communities and analyse the relationship between plant community types and environmental variables, respectively. A total of 68 woody plant species belonging to 63 genera and 44 families were identified. Four plant community types were identified: Albizia gummifera - Ekebergia capensis, Prunus Africana - Croton macrostachyus, Acacia abyssinica -Dombeya torrida and Schefflera abyssinica - Teclea nobilis. The CCA results showed that the variation of species distribution and plant community formation were significantly (P < 0.005) related to altitude, organic matter, aspect, slope, and phosphorus. The total basal area and density of woody species were 27.4 m2 ha−1 and 1079.3 individual ha−1, respectively. The regeneration status assessment of the forest revealed a poor regeneration status, which was linked to the presence of multiple disturbance factors such as cutting and charcoal production signs. Therefore, it needs immediate conservation to ensure sustainable use and management of the forest.
**Order of Authors:**	Liyew Birhanu
	Getaneh Moges, MSc.
	Nigussie Amsalu, PhD
**Additional Information:**	
**Question**	**Response**
**Financial Disclosure**<br><br>Enter a financial disclosure statement that describes the sources of funding for the work included in this submission. Review the submission guidelines for detailed requirements. View published research articles from *PLOS ONE* for specific examples.	The author(s) received no specific funding for this work.

This statement is required for submission and **will appear in the published article** if the submission is accepted. Please make sure it is accurate.<br><br>**Funded studies**<br>Enter a statement with the following details:<br>• Initials of the authors who received each award<br>• Grant numbers awarded to each author<br>• The full name of each funder<br>• URL of each funder website<br>• Did the sponsors or funders play any role in the study design, data collection and analysis, decision to publish, or preparation of the manuscript?<br><br>Did you receive funding for this work?	
**Competing Interests**<br><br>Use the instructions below to enter a competing interest statement for this submission. On behalf of all authors, disclose any competing interests that could be perceived to bias this work—acknowledging all financial support and any other relevant financial or non-financial competing interests.<br><br>This statement is required for submission and **will appear in the published article** if the submission is accepted. Please make sure it is accurate and that any funding sources listed in your Funding Information later in the submission form are also declared in your Financial Disclosure statement.<br><br>View published research articles from *PLOS ONE* for specific examples.	The authors declare that they have no competing interests.

**NO authors have competing interests**<br><br>Enter: *The authors have declared that no competing interests exist.*<br><br>**Authors with competing interests**<br><br>Enter competing interest details beginning with this statement:<br><br>*I have read the journal's policy and the authors of this manuscript have the following competing interests: [insert competing interests here]*<br><br>* typeset	
**Ethics Statement**<br><br>Enter an ethics statement for this submission. This statement is required if the study involved:<br><br>• Human participants<br>• Human specimens or tissue<br>• Vertebrate animals or cephalopods<br>• Vertebrate embryos or tissues<br>• Field research<br><br>Write "N/A" if the submission does not require an ethics statement.<br><br>General guidance is provided below. Consult the submission guidelines for detailed instructions. **Make sure that all information entered here is included in the Methods section of the manuscript.**	"N/A"

**Format for specific study types**<br><br>**Human Subject Research (involving human participants and/or tissue)**<br>• Give the name of the institutional review board or ethics committee that approved the study<br>• Include the approval number and/or a statement indicating approval of this research<br>• Indicate the form of consent obtained (written/oral) or the reason that consent was not obtained (e.g. the data were analyzed anonymously)<br><br>**Animal Research (involving vertebrate animals, embryos or tissues)**<br>• Provide the name of the Institutional Animal Care and Use Committee (IACUC) or other relevant ethics board that reviewed the study protocol, and indicate whether they approved this research or granted a formal waiver of ethical approval<br>• Include an approval number if one was obtained<br>• If the study involved *non-human primates*, add *additional details* about animal welfare and steps taken to ameliorate suffering<br>• If anesthesia, euthanasia, or any kind of animal sacrifice is part of the study, include briefly which substances and/or methods were applied<br><br>**Field Research**<br><br>Include the following details if this study involves the collection of plant, animal, or other materials from a natural setting:<br>• Field permit number<br>• Name of the institution or relevant body that granted permission	
**Data Availability**<br><br>Authors are required to make all data underlying the findings described fully available, without restriction, and from the time of publication. PLOS allows rare exceptions to address legal and ethical concerns. See the PLOS Data Policy and FAQ for detailed information.	Yes - all data are fully available without restriction

A Data Availability Statement describing where the data can be found is required at submission. Your answers to this question constitute the Data Availability Statement and **will be published in the article**, if accepted.<br><br>**Important:** Stating 'data available on request from the author' is not sufficient. If your data are only available upon request, select 'No' for the first question and explain your exceptional situation in the text box.<br><br>Do the authors confirm that all data underlying the findings described in their manuscript are fully available without restriction?	
**Describe where the data may be found in full sentences. If you are copying our sample text, replace any instances of XXX with the appropriate details.**<br><br>• If the data are **held or will be held in a public repository**, include URLs, accession numbers or DOIs. If this information will only be available after acceptance, indicate this by ticking the box below. For example: *All XXX files are available from the XXX database (accession number(s) XXX, XXX.).*<br>• If the data are all contained **within the manuscript and/or Supporting Information files**, enter the following: *All relevant data are within the manuscript and its Supporting Information files.*<br>• If neither of these applies but you are able to provide **details of access elsewhere**, with or without limitations, please do so. For example:<br><br>*Data cannot be shared publicly because of [XXX]. Data are available from the XXX Institutional Data Access / Ethics Committee (contact via XXX) for researchers who meet the criteria for access to confidential data.*<br><br>*The data underlying the results presented in the study are available from (include the name of the third party*	-

and contact information or URL).<br>• This text is appropriate if the data are owned by a third party and authors do not have permission to share the data.<br><br>* typeset	
Additional data availability information:	

Click here to access/download;Manuscript;Liyew PLOS ONE.docx

Plant community distribution along environmental gradient of Seqela Dry Afromontane Forest in Northwestern Ethiopia

Liyew Birhanu[1]*, Getaneh Moges[1], Nigussie Amsalu[1]

[1]Department of Biology, Debre Markos University, P.O. Box: 269, Debre Markos, Ethiopia

***Corresponding**

Author: liyewmtu@gmail.com

ABSTRACT

Dry evergreen Afromontane forests are severely threatened due to the expansion of agriculture, overgrazing by livestock, and human population growth. The objective of this study was to investigate the floristic composition, vegetation structure, and classification of plant communities and assess the relationship between plant community types and environmental variables in the study forest. Systematic sampling was used to collect vegetation and environmental data from 52 (20 m x 20 m) (400 m^2) plots established at every 200 m intervals along transect lines that were 100 m apart from each other. Density, DBH, basal area, frequency, and importance value indices (IVI) of woody species were computed to characterize the vegetation structure of the forest. Hierarchical cluster analysis and canonical Correspondence Analysis (CCA) with R software were used to identify plant communities and analyse the relationship between plant community types and environmental variables, respectively. A total of 68 woody plant species belonging to 63 genera and 44 families were identified. Four plant community types were identified: *Albizia gummifera* - *Ekebergia capensis*, *Prunus Africana* - *Croton macrostachyus*, *Acacia abyssinica* - *Dombeya torrida* and *Schefflera abyssinica* - *Teclea nobilis*. The CCA results showed that the variation of species distribution and plant community formation were significantly ($P < 0.005$) related to altitude, organic matter, aspect, slope, and phosphorus. The total basal area and density of woody species were 27.4 $m^2\ ha^{-1}$ and 1079.3 individual ha^{-1},

respectively. The regeneration status assessment of the forest revealed a poor regeneration status, which was linked to the presence of multiple disturbance factors such as cutting and charcoal production signs. Therefore, it needs immediate conservation to ensure sustainable use and management of the forest.

Key words: Canonical Correspondence Analysis, Hierarchical cluster analysis, Seqela dry Afromontane forest, Plant community

1. Introduction

Forests in Ethiopia play a crucial role in providing essential resources such as food, medicines, construction materials, wood, energy, timber, carbon storage, soil preservation, and nutrient cycling. They also serve as habitats for numerous animal and plant species (Fraticelli et al., 2013). However, the forest cover in Ethiopia has faced significant threats due to rapid population growth and land use change (Williams et al., 2005). According to a recent study (FAO, 2020), Ethiopia's forest cover has decreased by 16% between 1990 and 2020, despite the revised definition of forests that includes woodlands. Deforestation continues to be a major concern in the country. The diverse environmental conditions in Ethiopia, including variations in altitude, climate, and soil, as well as human interactions, result in varying plant community distribution, structure, and composition (Yemata and Haregewoien, 2022).

The dry evergreen Afromontane forests in Ethiopia are facing significant threats due to agricultural expansion, overgrazing by livestock, and population growth (Williams et al., 2005; Meragiaw et al., 2021; Birhanu and Yitayh, 2023). Understanding the distribution patterns of plant species and the factors influencing them is essential for effective biodiversity conservation (Magurran, 2004). In these forests, various studies have been

conducted on flora composition, vegetation structure, and distribution of plant community types in relation to environmental factors (e.g., Bekele, 1993; Teketay and Bekele, 1995; Yineger et al., 2008; Birhanu et al., 2021; Dagne and Birhanu, 2023). Despite the limited number of studies on ecological and floristic aspects in other dry Afromontane forest, including Seqela forest, there is still a need for further research to better understand the ecosystems and plant communities in these areas. The study aimed to investigate plant community types and the driving environmental factors shaping the existing vegetation in the study area, which could serve as a basis for taking effective conservation measures of the diminishing natural resource (Brooks et al., 2002). There are no earlier previous investigations on floristic composition, structure and regeneration status in the Seqela Dry Afromontane forest. We tested the following hypotheses: (1) the natural vegetation of the forest in the study area will have different plant community types and high species diversity (2) the plant distribution and diversity will be influenced by soil and topographic factors in the study area., (3) the regeneration status and potential of the study area is under good/fair status. More specifically, the objectives of this study were: (1) to document the woody species composition of the Seqela forest; (2) to investigate the major environmental factors determining species distribution and community composition in the Seqela forest; (3) to examine the vegetation structure and regeneration status of Seqela forest. Therefore, this study was conducted to fill the existing gap and serve as a tool for running effective conservation and sustainable utilization of the study forest.

2. MATERIALS AND METHODS

Description of the Study Area

The study was carried out in the Sekela Forest, which is located in Northwestern Ethiopia's Quarit District of the West Gojjam Zone of the Amhara Regional State (Fig. 1). The district is 418 kilometres and 233 kilometres northwest of Addis Ababa and Bahir Dar, respectively, in

the northwest region of Ethiopia. According to Friis et al. (2010), Sekela Forest is categorised as dry evergreen Afromontane Vegetation. The forest is located between 100°56'0" and 100°57'30" N latitude and between 370°28'0" and 370°28'30" E longitude. Eutricvertsols, a subset of vertisols, are the most common form of soil in the research area (Asaminew Tassew et al., 2011).

The 10-year climate data (2012-2021) for the Quarit station was obtained from the National Meteorological Services Agency (NMSA, 2022). The average annual rainfall in the district is 1393 mm. The average mean minimum and maximum temperatures in the study area are 100°C and 30.70°C, respectively, with an average annual temperature of about 20.10°C (Figure 2). The main crop types in the district include cereal crops, oil crops, pulses, and vegetables. Cereal crops include barley (*Hordeum vulgare*), wheat (*Triticum sp.)*, teff (*Eragrostis teff*), and maize (*Zea mays*). Oil crops include Niger seed (Guizotia abyssinica) and sunflower (*Carthamus tinctorius*). Pulses include faba bean (*Vicia faba*) and field pea (*Pisum sativum*). Vegetables include cabbage (*Brassica oleracea*), potatoes (*Solanum tuberosum*), garlic (*Allium sativum*), and onion (*Allium cepa*). Potatoes are the primary traded tuber crop (Gebey and Mekuriaw, 2013).

Sampling design

A reconnaissance study was conducted across the forest to determine the sampling plots for the collection of environmental and vegetation data, as well as to provide an overview of the site characteristics. To collect data on vegetation and the environment, a systematic sampling method was applied A total of 52 plots , 20 m x 20 m in size and 100 m far apart from each other were laid along the transects. The distance between each transect line was 200 m. Five smaller subplots of 5 *5 m^2, four at the corner, and one at the centre of the main plot, were established for seedling and sapling species collection (Masresha *et al*., 2019).

Vegetation data collection

Trees and shrubs were counted individually in each plot to record the species of woody plants. For shrubs and trees larger than 2.5 cm in diameter, the diameter at breast height (DBH) was measured and recorded. Mature shrubs and trees were defined as reaching more than 2.5 metres tall, whereas saplings between 1 and 2.5 metres and seedlings up to 1 metre were deemed mature. Using the modified 1–9 Braun-Blanquet scale, which Van Der Maarel (1979) transformed, the cover-abundance values of trees and shrubs were estimated. R statistical software version 4.12 (R Core Team, 2022) was used to import the three-column data for each species and perform several analyses, including ordination, diversity, and evenness, along with to cluster analysis.

Environmental data collection

For every plot within the forest, GPS was used to capture physiographic characteristics like longitude, latitude, and altitude. Each sample plot's slope was determined using a Suunto clinometer. Five locations, each sized 15 × 15 cm and with a depth of 0–30 cm, were used to gather soil samples from each main plot (four from each corner and one from the center) (Tadesse et al., 2008). After combining these samples to create a composite sample, about 1 kg was removed for examination. Before chemical tests, the soil samples were air dried, crushed, and sieved with a 2 mm screen size to remove organic waste and root particles.

Following standard protocols, measurements and analyses were carried out for pH, electrical conductivity (EC), cation exchange capacity (CEC), soil organic matter (SOM), total nitrogen (N), available phosphorus (P), and soil texture (sand, silt, and clay). The soil analysis was

assessed at Debre Markos Soil Research and Fertility Improvement's soil laboratory (Birhanu, 2020).

Data Analysis

Plant community classification

Using the estimated value of percept cover for each woody species, agglomerative hierarchical cluster analysis was performed in this work to classify the vegetation into plant community types using the free statistical program R version 4.12 (R Core Team, 2022). Based on the relative magnitude of their synoptic values, one or two main indicator tree or shrub species are chosen for identifying the community. A total of 64 plant species and 52 sample plots were used in the analysis.

Diversity and similarity indices

Shannon-Wiener diversity index and Shannon’s evenness were computed to describe the species diversity of the study area

$H' = -\sum_{i=1}^{s} Pi \ln pi$, where by (1)

H' = Shannon diversity index

s = number of species,

p= proportion of individuals or abundance of the ith species expressed as a proportion of total cover in the sample; and *ln* = natural logarithm.

Shannon's evenness index (J) was also calculated using: $J = {}^{H'}/_{H'max}$ (2)

where H' = Shannon–Wiener Diversity Index; and H'max = lns, where s is the number of species in the sample.

Ordination

A multivariate method called ordination is used to show interactions in a small-scale region between species, plots, and environmental variables (McCune and Grace, 2002). Using SAS software, a preliminary analysis of the data was performed by computing Pearson's correlation coefficient to find significant correlations between environmental variables and remove auto-correlated variables from the ordination analysis. The present study used Canonical Correspondence investigation (CCA) to look at the links between environmental variables and vegetation. Thirteen environmental variables (pH, EC, total nitrogen, available phosphorus, CEC, and OM) as well as 52 sample plots, 64 species, and moisture content have been included in the analysis. To determine the effect of environmental factors on the distribution of plant communities, an Adonis test was performed. Cover abundance values for plant species and information on significant environmental variables were shown in the CCA ordination plot.

Structural data analysis

Density, basal area, frequency, IVI, and DBH class distributions were calculated for each woody plant species used for the description of vegetation structure. Thus, the following formulas were used to calculate the frequency, density, and basal area of woody species.

$$Frequency\ (F) = \frac{Number\ of\ plots\ in\ which\ a\ species\ occurs}{Total\ number\ of\ plots} \times 100 \qquad (3)$$

$$Density = \frac{Total\ number\ of\ stems\ of\ all\ trees}{Sample\ size\ in\ hectare} \qquad (4)$$

$$BA = \left(\frac{DBH}{2}\right)^2 \times \pi \qquad \text{With } (\pi = 3.14) \text{ where} \qquad (5)$$

DBH is diameter at breast height

An importance value index (IVI) was computed for all woody species based on their relative density (RD), relative dominance (RDO), and relative frequency (RF). This index is used to determine the overall importance of each species in the forest system (Kent, 2012).

The Importance Value Index (IVI) for each woody species was computed using the following formula

$$RDO = \frac{BA}{TBA} \times 100 \qquad (6)$$

Where, RDO= relative dominance, BA= Basal area, TBA= total basal area

$$Relative\ Density = \frac{Number\ individuals\ of\ each\ species\ per\ ha}{Total\ number\ of\ individuals\ of\ all\ species\ per\ ha} \times 100 \qquad (7)$$

$$Relative\ Frequency = \frac{Number\ of\ sample\ plots\ containing\ a\ species}{Sample\ units\ for\ all\ species\ of\ the\ sample} \times 100 \qquad (8)$$

$$IVI = Relative\ Density + Relative\ Dominance + Relative\ Frequency, \qquad (9)$$

Regeneration status of the forest

The regeneration status of the forest was analysed by comparing the density ratios between seedlings and mature individuals, seedlings and saplings, and saplings and mature individuals. When seedlings are greater than saplings and saplings greater than adults, the status has fair regeneration, whereas seedlings are greater than or less than saplings and

saplings less than adults; the status is poor regeneration, and the species survives only in the sapling stage (saplings may be less than or greater than adults), and a species is present only in adult form it considered as not regenerating (Dhaulkhandi et al., 2008).

3. Results

Woody species composition

In the study area, 68 species of woody plants were identified, belonging to 44 families and 63 genera. Out of them, 64 species were found inside the plots, and 4 species—*Maytenus arbutifolia, Ficus sur, Lippia adoensis, and Cobretum molle*—were found outside the plot (Appendix 1). Of the 64 species of woody plants that have been found, woody lianas make up the least represented growth form, accounting for 10.94% (7 in number) of the species. Of these, trees make up 48.44% (31 in number), and shrubs bring up 40.62% (26 in number) (Appendix 1).

Plant community types

Based on the hierarchical cluster analysis performed in the study area, four plant community types were identified (Figure 3). These communities were grouped as follows: *Prunus africana* - *Croton macrostachyus; Albizia gummifer* - *Ekebergia.capensis; Acacia abyssinica* - *Dombeya torrida; Schefflera abyssinica* - *Teclea nobilis*

Albizia gummifera- Ekebergia capensis Community Type I

This community type was distributed between altitudinal ranges of 2314 –2520 m.a.s.l. The dominant tree species are Albizia. *gummifera, Ekebergia.capensis, Croton macrostachyus, Erythrina brucei, Pittosporum viridiflorum and Olea europaea* subsp. *Cuspidate.* The shrub species *such as Vernonia amygdalina, Maesa lanceolata, Maytenus addat, Clutia abyssinica, Rosa abyssinica, Vernonia myriantha and Bridelia micrantha* were highly dominant. The

most dominant woody Lianas included in this community were *Zehneria scabra* and *Periploca linearifolia.* This community type is represented by 52 species, being the richest in a number of species among the four community types.

***Prunus africana- Croton macrostachyus* Community Type II**

The altitudinal range of this community lies between of 2378 – 2441 m.a.s.l. The most dominant tree species were *Prunus africana, Olinia rochetiana, Apodytes dimidiata* and *Croton macrostachyus.* The shrub layer is dominated by Osyris *quadripartita, Calpurnia aurea, Asparagus africanus, Bersama abyssinica* and *Rytigynia neglecta*. Woody Liana such species *Jasminum abyssinicum, Urera hypselodendron, Clematis longicauda* and *Phytolacca dodecandra* were *dominant dodecandra.* This community is comprised of 34 species.

***Acacia abyssinica -Dombeya torrid* Community Type III**

This community type was located between the altitudinal ranges of 2377 – 2577 m.a.s.l. The dominant plant species from this community were *Acacia abyssinica*, *Dombeya torrida, Allophylus abyssinica, Maytenus obscura, Myrica salicifolia* and *Juniperus procera from* the tree layer and *Dovyalis abyssinica, Capparis tomentosa, Rubus steudneri, Rumex nervosus and Otostegia integrifolia* from shrub layer. This community is comprised of 48 species.

***Schefflera abyssinica - Teclea nobilis* CommunityType IV**

This community type was located between altitudinal ranges of 2335 – 2432 m.a.s.lThe dominant plant species in the community were *Schefflera abyssinica, Teclea nobilis, Euphorbia abyssinica, Dracaena steudneri, Solanecio gigas, Laggera tomentosa, Rhus glutinosa* and *Discopodium penninervium* from tree and shrub layer. This community is comprised of 27 species

Species richness, evenness, and diversity of community types in the Sekela forest

We calculated the Shannon-Wiener diversity index for each of the four types of plant communities. As a result, according to Shannon's diversity, community 1 had the greatest species diversity and richness, followed by community 3, and community 4 had the lowest. Conversely, communities 1 and 2 were followed in order of evenness value, with community 3 having the greatest value. Among all types of communities, Community 4 had the lowest evenness scoring (Table 2).

Similarity among community types in the Sekela forest

To find the differences in species composition between the four community types, Sorensen's similarity coefficient (Ss) was calculated. Among all communities, the overall similarity coefficient varies from 31 to 45%. Communities 1 and 2 had the next-most similarity (43%) after C1 and C3 (45%). Relatively little similarity (31%) was found between Communities 3 and 4. (Table 3).

Relationship between plant community types and environment variables

The gradient length of 3.69 is greater than 3, as shown by the DCA used to choose the optimal ordination technique. As a result, CCA was accepted as an adequate ordination for examining the interactions between the environmental variables and the vegetation. The longest DCA axis for the Sekela forest data set in the present study was 3.69, indicating the heterogeneity of the data as presented in (Table 4). To evaluate the connections between community type and environmental variables, a study using the unimodal response model is suggested. The Canonical correspondence analysis (CCA) ordination method is employed in this analysis (Birhanu 2020).

CCA Ordination

Thirteen environmental factors were taken into consideration in the ordination analysis: aspect, pH, phosphorus, sand, clay, silt, slope, nitrogen, moisture content, altitude, and Cation Exchange Capacity. As Table 5 and Figure 4 indicate, the only factors that significantly ($p < 0.05$) correlated with species compositions and their distributions were altitude, OM, slope, aspect, and phosphorus. The canonical correlation analysis (CCA) diagram showed that aspect, av P, and OM were the main variables related to the first axis, and altitude and slope were the major factors associated with the second axis (Figure 4, Table 6). The first and second axes' respective eigenvalues were 0.34 and 0.22.

For the first and second axes, there was heterogeneity in patterns of plant species distribution and plant community formation of 41% and 22.9%, respectively. The results showed that the first two axes accounted for 63.9% of the variation in patterns of plant species distribution and plant community formation. Thus, it was sufficient to reflect the relationship between species and environmental factors using the first two axes.

Vegetation structure

The distribution of woody plant species in various DBH classes was analyzed and classified into five categories: DBH classes 1 = 2.5-10 cm, 2 = 10.1-20 cm, 3 = 20.1-30 cm, 4 = 30.1-40 cm, and 5 = > 40.1 cm. The first DBH class (2.5-10 cm) had a greater density of woody individuals; 176 of them made up 28.8% of the total. 235 individuals, representing 24.5% of the total, were in the second DBH class (10.1–20 cm) density distribution. The density distributions for the remaining four classes were, in order, 177 (18.5%), 119 (12.4%), 72 (7.5%), and 80 (8.3%). From lower to higher DBH classes, the density distribution of woody individuals showed a declining trend.

In the Seqela natural forest, the overall density of woody species per hectare was 1079.3. *Croton macrostachyus* (88.46 individuals per hectare) was the species with the highest density, followed *by Calpurnia aurea* (68.27 individuals per hectare), *Albizia gummifera* (70.19 individuals per hectare), and *Olinia rochetiana* (78.37 individuals per hectare). *Albizia gummifera* (51.92%), *Croton macrostachyus* (44.23%), *Olinia rochetiana*, and *Teclea nobilis* (36.54%) were the most common woody species in the Seqela natural forest. *Erythrina brucei*, *Juniperus procera*, *Hagenia abyssinica*, *Myrsine africana*, *Laggera tomentosa*, and *Rumex nervosus* were the least common woody species, occurring in 1.92% of cases each. The total basal area of woody plant species with a DBH greater than 2.5 cm was 27.4 m2 per hectare. The basal area of individual plant species increased as their DBH class increased, but their number decreased. Species such as *Erythrina brucei* (6.27 m2/ha), *Prunus africana* (3.67 m2/ha), *Acacia abyssinica* (3.55 m2/ ha), and *Apodytes dimidiate* (2.59 m2/ha) and *Schefflera abyssinica* (2.04 m2/ha) contributed the largest basal area (Table 8).

Importance Value Index (IVI) of Species

Erythrina brucei (IVI = 11.24), *Prunus africana* (8.68), Croton *macrostachyus* (IVI = 7.38), *Albizia gummifera* (IVI = 9.03), and *Acacia abyssinica* (7.02) were the most dominant and ecologically significant species in the Seqela natural forest. Conversely, species like *Vernonia amygdalina*, *Juniperus procera*, *Hagenia abyssinica*, and *Dracaena steudneri* had lower IVI values (less than 1) (Table 9).

Regeneration status of woody plants in Seqela natural forest

The regeneration status of selected woody plant species in the Seqela forest was assessed to predict the forest's future fate. The regeneration status of the Seqela forest was classified as poor regeneration, as seedlings (44.7 individuals/ha) < saplings (68.3 individuals/ha) < mature trees (461.1 individuals/ha), indicating a J-shaped pattern with a small number of

seedlings and saplings compared to mature trees (Figure 5). Woody plant species such as *Schefflera abyssinica*, *Apodytes dimidiata*, *Erythrina brucei*, *Hagenia abyssinica*, *Juniperus procera*, *Myrica salicifolia*, and *Prunus africana* were not represented by both seedling and sapling stages. *Pittosporum viridiflorum*, *Acacia abyssinica*, *Albizia gummifera*, *Buddleja polystachya*, *Clausena anisata*, *Croton macrostachyus*, *Dombeya torrida*, *Dovyalis abyssinica*, *Euphorbia abyssinica*, *Olea europaea*, *Olinia rochetiana*, *Osyris quadripartita*, *Rhamnus prinoides*, *Rytigynia neglecta*, *Teclea nobilis*, and *Vernonia amygdalina* exhibited fair regeneration status. Conversely, woody species such as *Allophylus abyssinicus*, *Buddleja davidii*, *Discopodium penninervium*, *Dracaena steudneri*, *Ekebergia capensis*, *Grewia ferruginea*, *Maytenus obscura*, and *Rhus vulgaris* showed poor regeneration status.

4. DISCUSSION

Woody species composition

In the Seqela forest, we found 68 woody species. In contrast to studies conducted in the dry Afromontane forest in Ethiopia, such as those conducted in the Zege Peninsula forest (Alelign et al., 2007) with 113 species, Tara Gedam and Abebaye forests (Zegeye et al., 2011) with 143 species, and the forest patches in the Dega Damot district (Birhanu et al., 2021) with 106 woody species, the study area showed lower species richness in woody species. The study area's species richness was higher than that of other dry Afromontane forests in Ethiopia, like the 64-species Denkoro Forest in Wello (Ayalew, 2003), the 50-species Zengena Forest (Tadele et al., 2014), the 66-species Kuandisha Forest (Berhanu et al., 2017), and the 57-species Amoro Forest (Birhanu et al., 2018).

Fabaceae, Euphorbiaceae, and Rosaceae were among the plant families that were the most common in the study area. The order of families in terms of species richness is consistent with previous studies on dry evergreen Afromontane forests, which indicated the dominance

of Asteraceae (Yineger et al., 2008; Tilahun et al., 2011; Lulekal, 2014). Similarly, Berhanu et al. (2016) reported that the most frequent family in the Kuandisha dry evergreen Afromontane forest fragment was Fabaceae. According to Tadesse (2004), the two most prominent plant families in the flora of Ethiopia and Eritrea are the Asteraceae and Fabaceae. In Seqela Forest, the dominance of Asteraceae and Fabaceae may suggest that disturbances have occurred in the forest. Asteraceae plants are typically ruderal and thrive best in open, disturbed soil (Dagne and Birhanu, 2023). According to Dagne and Birhanu (2023), the ability of Fabaceae to fix atmospheric nitrogen helps them to survive on soils deficient in nutrients, which accounts for their success in taking over disturbed habitats.

Plant communities

In the Seqela forest, four plant communities were identified based on the findings of the hierarchical cluster analysis. However, species diversity and richness differed among plant communities. For instance, communities 1 and 3 exhibited the highest species richness and diversity. Conversely, community 4 had the lowest species richness and diversity compared to the other communities. The reason for the high species diversity and richness in communities 1 and 3 may be due to the highest altitudinal ranges in which these communities are found, ranging from 2378 to 2441 and 2377 to 2577 m.a.s.l., respectively (Birhanu, 2020). Additionally, community 4 was the most disturbed community, likely due to agricultural expansion and overgrazing by livestock.

Relationship between plant community types and environment variables

The topography (aspect, slope, and altitude) and soil variables combined together to shape the distribution of plant communities in the Seqela forest. The main environmental factors that explained variations in plant species distribution and patterns of plant community formation

in the research area were aspect, slope, altitude, OM, and av P, according to the CCA results. According to Paudel and Vetaas (2014), aspect was shown to be the most significant environmental variable in determining vegetation variation. This could be attributed to variations in incoming solar radiation that impact temperature and moisture levels. Plant growth and the creation of plant communities are strongly influenced by the temperature and moisture of the soil. Slope and altitude were the primary factors influencing vegetation distribution, according to similar studies conducted in other dry Afromontane forests in Ethiopia (Shiferaw et al., 2018; Birhanu et al., 2018). A similar study by Birhanu et al. (2021) also confirms that organic matter and av-P were the most important constraining variables in plant community formation of the study area.

Vegetation structure

The woody plant species distribution in Seqela Forest showed an inverted J-shape pattern, suggesting that small to medium-sized DBH trees dominated the forest rather than large-sized trees. This could be because local communities used these species for various purposes.

The total density of woody species in the Seqela forest was 1079.3 individuals per hectare. This was less than the results of studies conducted by Alelign et al. (2007) in the Zegie Peninsula Forest (3318 individuals per hectare), Berhanu et al. (2016) in the Kundasha Forest (3086 individuals per hectare), and Birhanu et al. (2018) in the Amoro Forest (2860 individuals per hectare). The density was also higher than other forests, such as Wanzaye natural forest (498) (Getnet, 2018). The degree of anthropogenic interference in various forests may account for this variation in density. In the research area, species with the highest density values were *Clutia abyssinica*, *Croton macrostachyu*s, *Albizia gummifera*, *Olinia rochetiana*, and *Calpurnia aurea*. This might be because the species is small, resulting to a greater total count within a given plot size and a fairly excellent ability for regeneration in dry

situations under shade. On the other hand, low-density species may be associated with increased grazing and tree and shrub cutting for charcoal and firewood (Birhanu et al., 2022). The most common species in Seqela woodland were *Albizia gummifera*, *Croton macrostachyus*, *Olinia rochetiana*, *Urera hypselodendron*, and *Teclea nobilis*. These species were pioneer species, as reported by Bekele (1993). *Albizia gummifera* and *C. macrostachyus* were also the most frequent species in Tara Gedam and Abebaye forests, respectively (Zegeye et al., 2011). The total basal area of the Seqela forest was 27.4 m2 per hectare, and the basal area of individual plant species increased as their DBH class increased but their number decreased. The study area had relatively less total basal area than some dry Afromontane forests in Ethiopia, such as Tara Gedam Forest and Abebaye forests (Zegeye et al., 2011) and Wof washa forest (Bekele, 1994), and Dega Damot district forest patches (Birhanu et al., 2019). On the other hand, the basal area was greater than other forests, such as Amoro forest (Birhanu et al., 2018) and Kundasha forest (Birhanu et al., 2016). The total basal area of the study area was comparable to that of the Zengena forest (Tadele et al., 2014). Species such as *Erythrina brucei*, *Prunus africana*, *Acacia abyssinica*, *Apodytes dimidiate*, *Albizia gummifera*, and *Schefflera abyssinica* had high basal area due to their large size, even though they had low density. The Importance Value Index (IVI) value was an important parameter used to determine the significance of species in a given ecosystem for conservation purposes (Lamprecht, 1989). Species with lower IVI values required high conservation efforts (Tadele et al., 2014), while those with higher IVI values needed monitoring and management (Birhanu et al., 2022).

Regeneration status

The regeneration status of natural vegetation is considered poor regeneration if seedlings are greater than or less than saplings and saplings are less than adults (Dhaulkhandi et al., 2008). In the case of the Seqela forest, the regeneration status was considered as poor regeneration

since seedlings (44.7 individuals per hectare) < saplings (68.3 individuals per hectare) < mature trees (461.1 individuals per hectare). The possible reasons for the existence of poor regeneration status in the forest might be grazing intensity and cutting of young plants for various purposes.

5. Conclusions

The woody species composition of the Seqela forest indicates a good level of woody species richness in the area. Four community types, including *Albizia gummifera* - *Ekebergia capensis*, *Prunus Africana* and *Croton macrostachyus*, *Acacia abyssinica* and *Dombeya torrida*, - *Schefflera abyssinica* - *Teclea nobilis*, were identified. Canonical correspondence analysis (CCA) showed that aspect, altitude, slope, OM, and P were the primary factors of plant distribution patterns and plant community types. This suggests that the distribution of plant species and the formation of plant communities are heavily reliant on these environmental conditions. Species with high densities at lower DBH classes were characteristic of the general vegetation structure of Seqela Forest; this may be because shrubby species dominated. The assessment of the forest's regeneration status showed a poor degree of regeneration, which was associated with several disturbance causes such as cutting and signs of charcoal production. As a result of these disturbances, some plant species had no seedlings or saplings, while others had both seedlings and saplings. Species like *Schefflera abyssinica*, *Apodytes dimidiata*, *Erythrina brucei*, *Hagenia abyssinica*, *Juniperus procera*, *Myrica salicifolia*, and *Prunus africana* were among those with no seedlings and saplings.

Data Availability

The data used to support the findings of this study are available from the corresponding author upon reasonable request.

Conflicts of interest

The authors declare that they have no competing interests

Acknowledgments

Agriculture and Rural Development Office of the Quarit District as well as to the respective chairpersons is also highly acknowledged.

Supplementary Materials

Appendix Table 1: List of woody plant species collected from Seqela forest Quarit District

Refrences

Alelign A , Teketay D, Yemshawy, & Sue Edwards (2007) Diversity and status of regeneration of woody plants on the peninsula of Zegie, northwestern Ethiopia.*Tropical Ecology* 48(1)**:** 37-49, 2007 ISSN 0564-3295.

Ayalew A (2003) A Floristic composition and structural analysis of Denkoro Forest, South Wello.M.Sc. Thesis. Addis Ababa University

Bekele T (1993) Vegetation ecology of remnant Afromontane forests on the central plateau of Shewa, Ethiopia. Sv. växtgeografiska sällsk.

Berhanu A, Demissew S, Woldu Z and Didita M (2017) Woody species composition and structure of KuandishaAfromontane forest fragment in northwestern Ethiopia.Journal of Forestry Research 28: 343-355.

Birhanu L, Bekele T and Demissew S (2018) Woody species composition and structure of AmoroForest in West Gojjam Zone, Northwestern Ethiopia. *Journal of ecology andthe natural environment***10** (4): 53-64.

Birhanu L (2020).Ecological Study of the Vegetation and Land Use/ Land Cover Change analysis in Dega Damot District, West Gojjam Zone, Amhara Region, NorthwesternEthiopia PhD thesis , Addis Ababa University, Ethiopia.

Birhanu L, BekeleT., Binyam Tesfaw, and Sebsebe Demissew, (2021) Relationships

between topographic factors soil and plant communities in a dry Afromontane forest patches of North western Ethiopia. PLOS ONE, 16:1–18.

Birhanu L, Bekele T, Tesfaw B, Demissew S, (2022). Soil seed bank composition and aboveground
vegetation in dry Afromontane forest patches of Northwestern Ethiopia,Trees, Forests and People,Volume 9, https://doi.org/10.1016/j.tfp.2022.100292.

Dagne Y, Birhanu L (2023) Floristic composition and plant community distribution along environmental gradients in Guard dry Afromontane forest of Northwestern Ethiopia. BMC Ecol Evol., 28;23(1):43. doi: 10.1186/s12862-023-02154-6.

Dhaulkhandi, M., Dobhal, A., Bhatt, S. and Kumar, M. (2008) Community structure and regeneration potential of natural forest site in Gangotri , India. J Basic Appli Sci.
FAO, *Global Forest Resources Assessment (2020): Main report* (Food and Agriculture Organization of the United Nations, Rome,

Fraticelli M, Perdriault M, Pinsart C, Rafert J (2013) La gouvernance des forêts au Cameroun. CED/AGTER, Nogent sur Marne, 133 p

Friis, I., Demissew S and van Breugel, P. (2010). Atlas of the potential vegetation of Ethiopia. The royal Danish Academy of Sciences and Letters, Copenhagen, DK

Gebey T and Mekuriaw Z (2013). Diagnosis and intervention plans for west Gojjam zone, Amhara Region, Ethiopia

Getnet A (2018). Woody Species Composition, Diversity and Vegetation Structure of Dry Afromontane Forest, Ethiopia.*Journal of Agriculture and Ecology Research International.**16***(3): 1-20.

Kent M (2012) Vegetation Description and Data Analysis a Practical Approach 2nd Eds. Blackwell Publishing, UK.

Lamprecht, H., (1989) Tropical forest ecosystems and their tree species. Silviculture in Tropics, Possibilities, and Methods for Their Long-Term Utilization. Institute of for silviculture of the University of Gottingen, Technical Cooperation of Federal Republic of Germany, Berlin, p. 296.

Lulekal E (2014) Plant diversity and ethnobotanical study of medicinal plants in Ankober District, North ShewaZone of Amhara Region, Ethiopia. Ph.D. Dissertation, Addis Ababa University. 312p.

Magurran AE (2004) Measuring biological diversity. Blackwell Science ltd, Malden, p 132

Masresha G, Asnakew M, Chekole G, Melese G (2019) Comparative study on floristic composition vegetation structure and regeneration status of woody forests in case of GondTeklehaimaot and Arsema Monastery Forest, Department of Biology, College of Natural and Computational Sciences, University of Gondar, Ethiopia.

McCune B, Grace J, Urban D. (2002) Analysis of ecological communities. Gleneden Beach, OR: MjM software design.

Meragiaw, M, Woldu, Z, Martinsen, V. (2021) Floristic composition and structure of the Kibate Forest along environmental gradients in Wonchi, Southwestern Ethiopia. *J. For. Res.* **32**, 2669–2682, https://doi.org/10.1007/s11676-021-01305-z

Paudel S and O. R. Vetaas, (2014) “Effects of topography and land use on woody plant species composition and beta diversity in an arid Trans-Himalayan landscape, Nepal,” *Journal of Mountain Science*, vol. 11, no. 5, pp. 1112–1122

Shiferaw W, Bekele T, Demissew S (2018) Anthropogenic effects on floristic composition, diversity and regeneration potential of the Debrelibanos Monastery forest patch, central Ethiopia. J For Res. ;30(6):2151–61

Tadele D, Lulekal E, Damtie D and Assefa A (2014) Floristic diversity and regeneration status of woody plants in Zengena forest, a remnant montane forest patch in northwestern Ethiopia. Journal of forestry research 25 (2):329–336.

Tadesse G, Bekele T, Demissew S (2008) Dryland woody vegetation along an altitudinal gradient on the eastern escarpment of Wolo, Ethiopia. SINET: Ethiop J Sci 31:43–54

Tadesse M (2004) Asteraceae (Compositae). In: Hedberg, I., Friis, I., Edwards, S., (eds). Flora of Ethiopia and Eritrea. Vol. 4(1). The National Herbarium, AAU, Addis Ababa & Uppsala.

Tassew A, Teffera B, Jafar E, Ashagrie E, Mulugeta G, Tesfaye G, Adimasu S, Limenih S, Genet T, Yihenew G. Selassie andLeyew Z (2011). Comprehensive study on research, development and management interventions in Birr sub-watershed, Quarit district, West Gojjam zone, Amhara national regional state

Teketay D, Bekele T(1995) Floristic composition of Wof- Washa natural forest, Central Ethiopia: Implications for the conservation of biodiversity. Feddes Repertorium. ; 106 (1- 5):127–47.

Tilahun A, Soromessa T, Kelbessa E, and Dibaba A (2011) Floristic composition and community analysis of Menagesha Amba Mariam Forest

(Egdu Forest) in the central plateau of Shewa, Ethiopia.Ethiopian Journal of Biological Sciences**10** (2): 111-136.

Van der Maarel, E. (1979) Transformation of Cover abundance values in phytosociology and its effects on community similarity. *Vegetation **3**9*:97-114

Williams, S.D., J.L. Vivero, S. Spawls, Aneh S. & Ensermu K. (2004). Ethiopian Highlands. In: Mittermeier, R.A., P. Robles-Gil, M. Hoffmann, J.D. Pilgrim, T.M. Brooks, C.G. Mittermeier & G. Fonseca (eds.), Hotspots Revisited: Earth´s Biologically Richest and Most Endangered Ecoregions, pp. 262-273. CEMEX, Mexico city, Mexico.

Yemata G, Haregewoien G (2022) Floristic composition, structure and regeneration status of woody plant species in Northwest Ethiopia,Trees, Forests and People,Volume 9,https://doi.org/10.1016/j.tfp.2022.100291

Yineger H, Kelbessa E, Bekele T, Lulekal E (2008) Floristic composition and structure of the dry Afromontane forest at Bale Mountains National Park, Ethiopia. SINET: Ethiopian Journal of Science. 31(2):103–20.

Zegeye H, Teketay D and Kelbessa K (2011) Diversity and regeneration status of woody species in Tara Gedam and Abebaye Forests, north-westernEthiopia. Journal of Forestry Research 22 (3):315–328

List of Tables

Table 1 Synoptic table of species

Species Name	C-1	C-2	C-3	C-4
Albizia.gummifera	**6**	1.17	2.35	1
Ekebergia.capensis	**5.12**	1.17	0.88	0.42
Maesa.lanceolata	1.35	0	0	0.75
Carissa.spinarum	1.47	0.33	0.71	0
Rosa.abyssinica	1.12	0	0.94	0
Vernonia amygdalina	1.13	0	0	0
Vernonia.myriantha	1.12	1	0.18	0.17
Clutia.abyssinica	1.06	0.5	0.47	0

Erythrina.brucei	0.35	0	0	0
Maytenus addat	0.88	0.33	0	0
Bridelia.micrantha	0.41	0.33	0.12	0
Hypericum.quartinianum	0.35	0	0	0
Rhamnus.prinoides	0.29	0	0.06	0
Zehneria.scabra	0.18	0.17	0.06	0
Olea.europaea	0.71	0	0.12	0
Periploca linearifolia	0.29	0.17	0.12	0
Embelia.schimperi	0.18	0	0.12	0
Pittosporum.viridiflorum	0.76	0	0.12	0
Brucea.antidysenterica	0.47	0.33	0	0.33
Gnidia.glauca	0.18	0	0.12	0
Croton.macrostachyus	4.67	**6.33**	0.65	0
Prunus.Africana	0.71	**6.83**	0.82	3.42
Olinia.rochetiana	2	3.67	2.47	0
Calpurnia.aurea	1	2.33	0.88	0
Apodytes.dimidiata	0.76	2.33	1.18	1.67
Osyris.quadripartita	0.53	1	0.47	0
Bersama.abyssinica	0.82	0.83	0	0.25
Galiniera.saxifraga	0.47	0.67	0	0.17
Phytolacca.dodecandra	0.18	0.33	0	0
Jasminum.abyssinicum	0.29	0.5	0.12	0.08
Hagenia.abyssinica	0	0.33	0	0
Clematis longicauda	0.29	0.33	0.18	0.25
Urera.hypselodendron	0.41	0.83	0.41	0.58
Asparagus.africanus	0	0.33	0.06	0
Rytigynia.neglecta	0.24	1	0.24	0.67
Buddleja.davidii	0.06	0.83	0.71	0.42
Acacia.abyssinica	0	0	**3.41**	0
Dombeya.torrida	1.06	0.67	**3.06**	0.75
Allophylus.abyssinicus	0.65	1.67	2.35	0
Clausena.anisata	1.24	0.83	1.29	0
Buddleja.polystachya	1	0	1.24	0.42
Dovyalis.abyssinica	0.47	0.5	1.24	0
Myrica.salicifolia	0.29	1	1.47	0
Maytenus.obscura	0	0	1.82	

				0
Rhus.vulgaris	0.71	0.17	0.82	0
Otostegia.integrifolia	0.24	0	0.35	0
Acanthus.sennii	0.53	0	0.59	0
Capparis.tomentosa	0.12	0	0.29	0
Juniperus.procera	0	0	0.35	0
Rubus.steudneri	0.35	0	0.53	0.17
Rumex.nervosus	0	0	0.12	0
Grewia.ferruginea	0.12	0	0.82	0
Dodonaea.angustifolia	0.12	0	0.29	0
Maytenus.obscura	0	0	1.82	0
Myrsine.africana	0	0	0.12	0
Schefflera.abyssinica	0	0	0	**6**
Teclea nobilis	0.88	0.33	1.06	**4**
Discopodium.penninervium	0.82	0	0.29	2.92
Solanecio.gigas	0.35	1	0.12	1.5
Euphorbia.abyssinica	0.29	0	0	1.33
Ocimum lamiifolium	0	0	0.29	0.83
Laggera.tomentosa	0	0	0	0.25
Rhus glutinosa	0	0	0	0.5
Dracaena.steudneri	0	0	0	0.17

Table 2. Species Richness, Diversity and Evenness of the communities

Community type	Richness	Diversity H	Shannon evenness
1	52	3.39	0.863
2	34	2.99	0.847
3	48	3.35	0.865
4	27	2.64	0.801

Table 3. Sorensen's similarity of the communities

Community types

-	C-1	C-2	C-3	C-4
C-1	-			
C-2	0.43	-		
C-3	0.45	0.41	-	
C-4	0.38	0.34	0.31	-

Note: C-1: Albizia gummifer-Ekebergia.capensis, C-2: Prunus Africana-Croton macrostachyus, C-3: Acaciaabyssinica-Dombeya torrida and C-4: Schefflera abyssinica -vepris nobilis community types.

Table 4 Detrended correspondence analysis results of Sekela forest

	**CC1**	**CC2**	**CC3**	**CC4**
Eigenvalues	0.5231	0.3083	0.2267	0.2275
Decorana values	0.5451	0.3056	0.2090	0.1766
Axis lengths	3.6875	2.5179	2.9114	1.9855

Table 5 Results of function donis test of environmental variables (significant environmental variables are indicated by Asterix at their p-value)

Environmental variables	Df	Sums of Sqs.	Mean Sqs	F. Model	R^2	Pr (>F)
Slope (%)	1	0.7574	0.75742	3.4429	0.04864	0.01 **
Aspect	1	2.8341	2.83412	12.8827	0.18199	0.01 **
Altitude (m)	1	0.5114	0.51141	2.3246	0.03284	0.03*
MC	1	0.2986	0.29864	1.3575	0.01918	0.19
Sand	1	0.3544	0.35440	1.6109	0.02276	0.09.
Silt	1	0.1304	0.13041	0.5928	0.00837	0.74
Clay	1	0.2647	0.26471	1.2033	0.01700	0.18
PH	1	0.2393	0.23929	1.0877	0.01537	0.33
EC	1	0.1350	0.13501	0.6137	0.00867	0.75
OM	1	0.5804	0.58039	2.6382	0.03727	0.02 *
CEC	1	0.1748	0.17482	0.7947	0.01123	0.67
N	1	0.3728	0.37280	1.6946	0.02394	0.10
P	1	0.5595	0.55946	2.5431	0.03593	0.02 *
Residuals	38	8.3598	0.21999	0.53683		
Total	52	15.5727				1.000

Signif.codes: 0 ‘***’ 0.001 ‘**’ 0.01 ‘*’ 0.05 ‘.’ 0.1 ‘ ’

Table 6 Bi-plot scores for constrained variables

**Variables**	**CCA1**	**CCA2**	**CCA3**	**CCA4**
Slope	-0.22	-0.785	-0.251	0.44660
Aspect	0.92	0.081	0.241	-0.27716
Altitude	0.26	-0.807	0.480	0.16719
OM	0.38	-0.288	0.073	0.00035
P	0.60	0.334	0.162	0.62852
Eigenvalue	0 .3864	0.2149	0.1497	0.1043
Proportion Explained	0.4121	0.2292	0.1597	0.1112
Cumulative Proportion	0.4121	0.6413	0.8010	0.9122

Table 7 Distribution of woody plant species in different DBH classes Classes:1 = 2.5-10 cm, 2 = 10.1-20 cm, 3 = 20.1-30 cm, 4 = 30.1-40 cm, 5 = 40.1 - 50cm and 6 = > 50 cm in Seqela natural forest

No	DBH class	No of individuals	density/ha
1	(2.5-10 cm)	276	132.7
2	(10.01-20 cm)	235	113
3	(20 -30 cm)	177	85.1
4	(30.1-40)	119	57.2
5	(> 40.1)	72	34.6

Table 8 Basal areas of the top 6 dominant species in Seqela forest

No	Scientific Name	Basa area (m^2/ha)
1	*Erythrina brucei*	6.27
2	*Prunus Africana*	3.67
3	*Acacia abyssinica*	3.55
4	*Apodytes dimidiata*	2.59
5	*Albizia gummifera*	2.05
6	*Schefflera abyssinica*	2.04

Table 9: The highest and lowest (IVI) value of woody plant species in the study area

NO.	Plant species Name	R.F	R.D	R.DO	IVI	% IVI
1	*Acacia abyssinica*	1.55	0.535	12.954	15.039	7.07*
2	*Albizia gummifera*	5.232	6.503	7.49	19.225	9.03*
3	*Croton macrostachyus*	4.457	8.196	3.047	15.7	7.38*
4	*Dracaena steudneri*	0.388	0.222	0.42	1.03	0.48**
5	*Erythrina brucei*	0.969	0.089	22.86	23.918	11.24*
6	*Hagenia abyssinica*	0.193	0.089	0.0274	0.3094	0.15**
7	*Juniperus procera*	0.193	0.044	1.104	1.341	0.63**
8	*Prunus africana*	3.489	1.604	13.392	18.485	8.68*
9	*Vernonia amygdalina*	0.775	0.357	0.128	1.26	0.59**

* Indicates the highest value of IVI of woody plant species.

** Indicates the lowest value of IVI of woody plant species

List of Figures

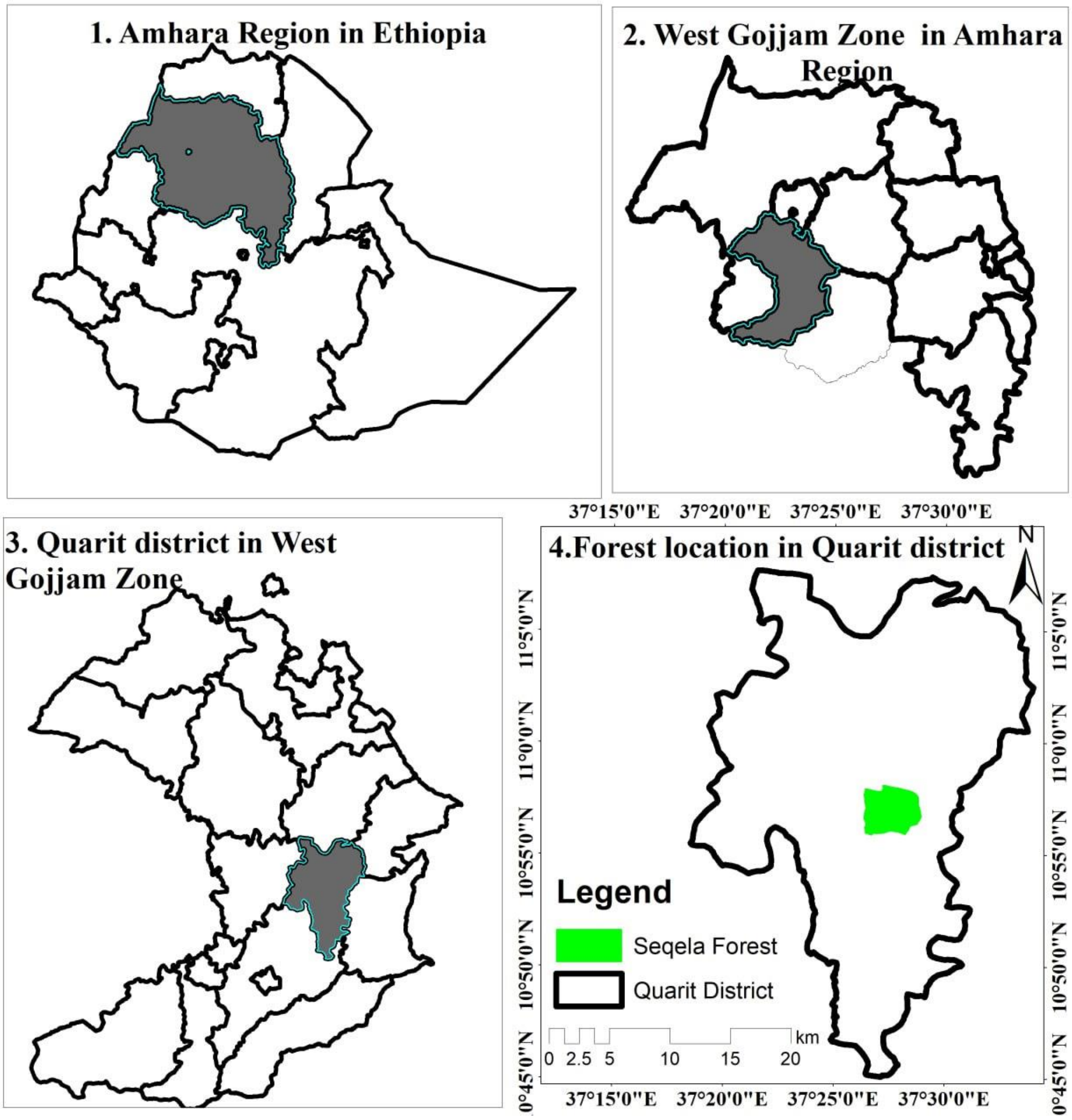

Figure 1. Sekela forest located in Quarit district of West Gojjam Zone, Amhara Region in Ethiopia

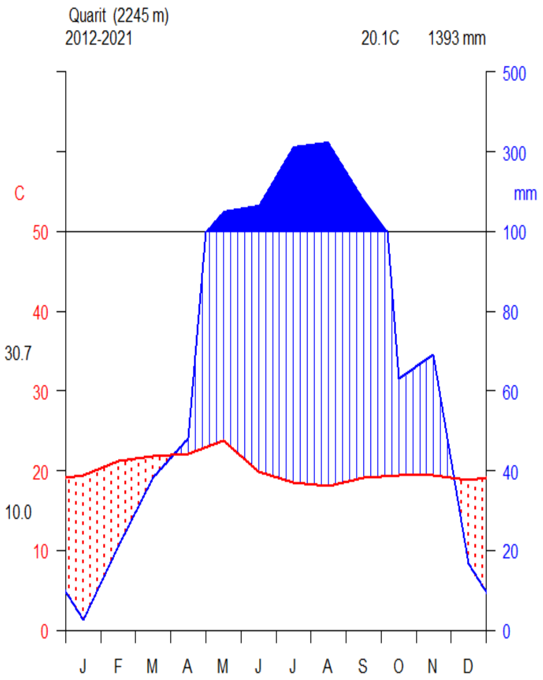

Figure 2. Climate diagram of Quarit (data Source: NMSA, 2022)

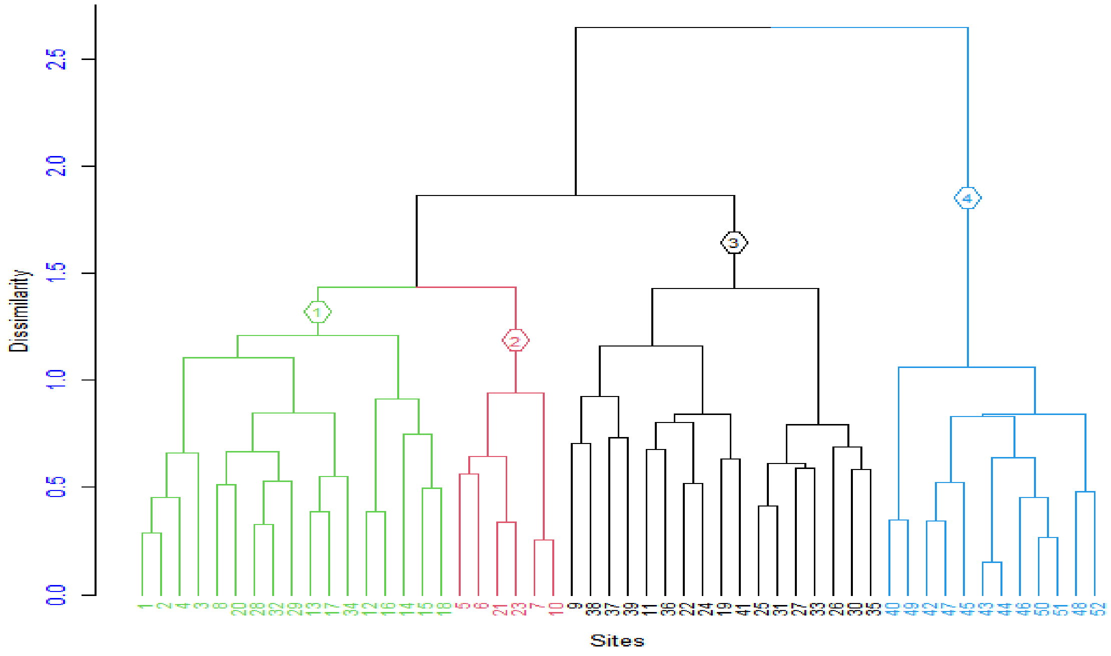

Figure 3. Dendrogram of the vegetation data obtained from hierarchical cluster analysis of the study area (Community type 1, 2, 3, and 4).

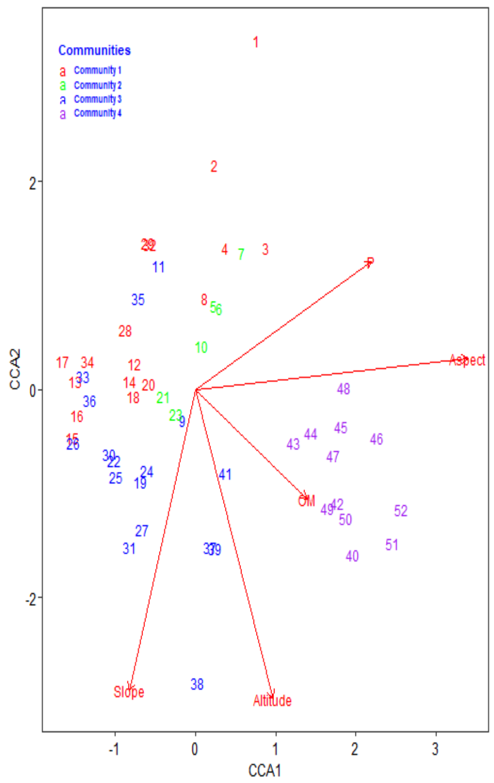

Figure 4. CCA ordination graph, significant environmental variables (p< 0.05), and plant community in the study area

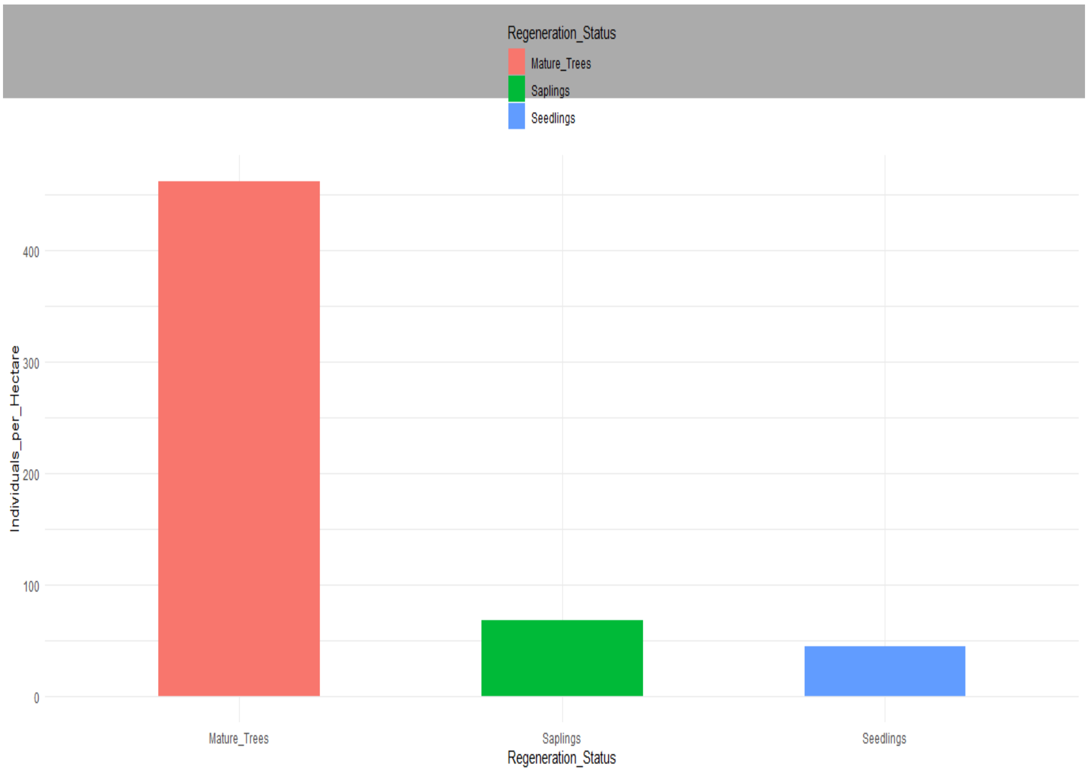

Figure5. Regeneration status of Seqela forest

Click here to access/download
Supporting Information
Appendix 1.docx
