# Supplementary material for: Woody species diversity, structure and community distribution along environmental gradients of Seqela Dry Afromontane forest in Northwestern Ethiopia
Source: PLoS One. 2025 Jan 17;20(1):e0313020. doi: 10.1371/journal.pone.0313020 (PMC11741620; doi:10.1371/journal.pone.0313020)
Supplement: Attachment — Submitted filename: Reviewers comments PLoS ONE.docx [file pone.0313020.s003.docx]

**Manuscript Number: PONE-D-24-05897**

**Plant community distribution along environmental gradient of Seqela Dry Afromontane**

**Forest in Northwestern Ethiopia**

PLOS ONE

**General comments**

The research work presents interesting results but the manuscript needs to improve the comments provided below. Botanical methods of plant species identification and deposition herbarium with voucher numbers are not clearly stated and need to be addressed before any further consideration. Furthermore, the title needs to be rethought. In the current state, it is not very informative.

**Specific comments:**

**Title**: I would suggest that your title needs to be modified in a way that it should accurately describe the contents of your manuscript. Because the phrase “Plant community distribution along environmental gradient” is not inclusive but rather a single part of your paper.

**Abstract**

Line 2: “human population growth” is an equivocal reason even if it will be an umbrella driver for the anthropogenic factors mentioned formerly.

Line 7: “100 m”, is it altitudinal or geographical distance?

Line 12: “A total of 68 woody plant species”, why not include herbaceous species according to your title?

Line 14: Replace “*Prunus Africana*” with “*Prunus africana*” see in Table 3. Additionally, check the revised taxonomic name of “*Acacia abyssinica*”?

**Keywords/phrases**: I would suggest to the authors that it is better to focus on specific and relevant words to your content rather than broad and generic long phrases like “Canonical Correspondence Analysis, Hierarchical cluster analysis”.

**Introduction**

In paragraph 1, the first two statements: The authors wrote the role of plant resources in the Ethiopian context but cited unrelated authors (Fraticelli et al., 2013)?

Lack of connectivity and flow of ideas between statements such as hypotheses and objectives.

**Methodology**

**Study area**

See the legend in your map and the caption here: “Figure 1. Sekela forest is located in the Quarit district of West Gojjam Zone, Amhara Region in Ethiopia” Which is correct “Sekela” or Seqela”?

“Sekela Forest is categorised as dry evergreen Afromontane Vegetation”, this needs to be rephrased because it contradicts with demonstration of the cliamadiagram in Figure 2.

Be consistent in your reference citation… See “Friis et al. (2010)” … “(Asaminew Tassew et al., 2011)”.

What is your reason for your selection of the sub quadrats 5 × 5 m^2^ for the collection of seedling and sapling plants?

**Vegetation data collection**

I find the sampling design rather vague.

Botanical methods, typically herbarium procedure are missed out.

**Data Analysis**

**Plant community classification**

There is a mix of information herein: “agglomerative hierarchical cluster analysis”? In your result section: “Based on the hierarchical cluster analysis performed in the study area, four plant community types were identified (Figure 3).”

Where is the anthropogenic factor analysis, which has been mentioned as a major threatening factor?

**Diversity and similarity indices**

Diversity is okay but where is the similarity index?

**Results**

Line 3: Replace “*Cobretum molle*” with “*Combretum molle*”

**Plant community types**

Replace “*Ekebergia.capensis*” with “*Ekebergia capensis*”

“Figure 3. Dendrogram”, here is my big question why not 2 types or three community types or else? Because the author did not show the value of the cophenetic correlation coefficient where they determined the cutree.

**Community type I.**

Replace “*Olea europaea* subsp. *Cuspidate*” with “*Olea europaea* subsp. *cuspidata*”

Community type II

“*Phytolacca dodecandra* were *dominant dodecandra*” needs to be revised.

**Similarity among community types in the Sekela Forest**

“To find the differences in species composition between the four community types, Sorensen's

similarity coefficient (Ss) was calculated.” My concern here: I did not see this part in the method section.

Figures 3, 4 and 5 need to increase their resolutions.

**References**

Please correct the font type “Alelign A , Teketay D, Yemshawy, & Sue Edwards (2007)”.
