# Supplementary material for: Woody species diversity, structure and community distribution along environmental gradients of Seqela Dry Afromontane forest in Northwestern Ethiopia
Source: PLoS One. 2025 Jan 17;20(1):e0313020. doi: 10.1371/journal.pone.0313020 (PMC11741620; doi:10.1371/journal.pone.0313020)
Supplement: Attachment — Submitted filename: Point by point response to the Reviewer Comments_PLOS ONE.docx [file pone.0313020.s005.docx]

**Manuscript number:** PONE-D-24-05897

**Title:** Plant community distribution along environmental gradient of Seqela Dry Afromontane Forest in Northwestern Ethiopia

**Journal Name:** PLOS ONE **Article type:** Research Paper

We would like to thank the reviewers for their efforts in reviewing the manuscript and providing valuable suggestions for improving the article. All comments and suggestions were addressed. The changes are marked in yellow color as shown in the revised manuscript. We detailed response for each comment is provided below.

**Editor**

Dear Dr. Birhanu,
Thank you for submitting your manuscript to PLOS ONE. After careful consideration, we feel that it has merit but does not fully meet PLOS ONE’s publication criteria as it currently stands. Therefore, we invite you to submit a revised version of the manuscript that addresses the points raised during the review process.
==============================
**ACADEMIC EDITOR:**

The paper needs a major revision.

**Author’s Response:** Thanks a lot for letting us revise our paper. Below are the responses to all reviewer comments.

**Reviewer #1**:

**General comments**

1. The research work presents interesting results but the manuscript needs to improve the comments provided below. Botanical methods of plant species identification and deposition herbarium with voucher numbers are not clearly stated and need to be addressed before any further consideration. Furthermore, the title needs to be rethought. In the current state, it is not very informative.

**Author’s Response:** Thanks a lot for this positive feedback**.** We are sorry that some of the information about Botanical methods of plant species identification and deposition herbarium was not included, and we have improved this in the revised manuscript.

**Specific comments:**

2. **Title**: I would suggest that your title needs to be modified in a way that it should accurately describe the contents of your manuscript. Because the phrase “Plant community distribution along environmental gradient” is not inclusive but rather a single part of your paper.
3. **Author’s Response:** We appreciate the reviewer observation. The title was corrected as Woody species diversity, Vegetation structure and Plant community distribution along environmental gradients of Seqela Dry Afromontane Forest in Northwestern Ethiopia

**Abstract**

4. Line 2: “human population growth” is an equivocal reason even if it will be an umbrella driver for the anthropogenic factors mentioned formerly.

**Author’s Response:** Thank you for this suggestion.

5. Line 7: “100 m”, is it altitudinal or geographical distance?
6. Line 14: Replace “*Prunus Africana*” with “*Prunus africana*” see in Table 3. Additionally, check the revised taxonomic name of “*Acacia abyssinica*”?

**Author’s Response:** Again, we appreciate the reviewer observation reviewer. We have corrected the genus Acacia to Vachellia in the revised version of the manuscript.We have corrected as *Vachellia abyssinica*

7. **/phrases**: I would suggest to the authors that it is better to focus on specific and relevant words to your content rather than broad and generic long phrases like “Canonical Correspondence Analysis, Hierarchical cluster analysis”.

**Author’s Response:** Thanks for this suggestion and improved clarity of the paper in the revised document.

**Introduction**

8. In paragraph 1, the first two statements: The authors wrote the role of plant resources in the Ethiopian context but cited unrelated authors (Fraticelli et al., 2013)

**Author’s Response:**	Thank you for this suggestion.

**Methodology**

**Study area**

9. See the legend in your map and the caption here: “Figure 1. Sekela forest is located in the Quarit district of West Gojjam Zone, Amhara Region in Ethiopia” Which is correct “Sekela” or Seqela”?

**Author’s Response:**	Seqela is the correct one.The correction has been made in the revised manuscript

10. Seqela Forest is categorised as dry evergreen Afromontane Vegetation”, this needs to be rephrased because it contradicts with demonstration of the cliamadiagram in Figure 2. **Author’s Response:***Olea europaea* subsp. *cuspidata*, *Juniperus procera,* and *Podocarpus falcatus* are indicator species to the dry Afromontane forest. Therefore, *Olea europaea* subsp. *Cuspidate* species is found in the Seqela forest. Actually the vegetation type classification based on elevation, climate and indicator species…. Thus it is categorised as dry evergreen Afromontane forest and grassland complex (DAF).
11. Be consistent in your reference citation… See “Friis et al. (2010)” … “(Asaminew Tassew et al., 2011)”.

**Author’s Response:** Thank you very much for your excellent observation. We have corrected any inconsistency written of reference citation

**Vegetation data collection**

12. I find the sampling design rather vague. Botanical methods, typically herbarium procedure are missed out.

**Author’s Response:** Thanks for this suggestion and improved clarity of the paper in the revised manuscript.

**Data Analysis**

**Plant community classification**

13. There is a mix of information herein: “agglomerative hierarchical cluster analysis”? In your result section: “Based on the hierarchical cluster analysis performed in the study area, four plant community types were identified (Figure 3).”

**Author’s Response:** Agglomerative hierarchical cluster analysis. We have corrected the inconsistency writing of this word.

14. Where is the anthropogenic factor analysis, which has been mentioned as a major threatening factor?

**Author’s Response:** No anthropogenic data collection and analysis in our study.

**Diversity and similarity indices**

18. Diversity is okay but where is the similarity index?

**Author’s Response:** We have added the information in the method section of revised manuscript.

**Results**

19. Line 3: Replace “*Cobretum molle*” with “*Combretum molle*”

**Author’s Response:** Corrected in the revised manuscript.

**Plant community types**

20. Replace “*Ekebergia.capensis*” with “*Ekebergia capensis*”

**Author’s Response:** Corrected in the revised manuscript.

21. “Figure 3. Dendrogram”, here is my big question why not 2 types or three community types or else? Because the author did not show the value of the cophenetic correlation coefficient where they determined the cutree.

**Author’s Response:** We appreciate the reviewer observation. We agree that before doing cluster analysis, we need to determine the optimum cluster number (K). Among the methods we use to decide the optimum cluster are Elbow, silhouette*,* gap statistics and K-means partitioning using cascadeKM (calinski) and Similarity Ratio. We have access to scripts for each method. We tried to check the matrified vegetation data with elbow method and simple visual observation during the field work. Accordingly, we have done full analysis and found 4 plant community types. BTW, methods use to decide the optimum cluster alone may not show a clear classification. So visual observation during the field work also helps.

**Community type I.**

Replace “*Olea europaea* subsp. *Cuspidate*” with “*Olea europaea* subsp. *cuspidata*”

**Author’s Response:** corrected in the revised manuscript.

Community type II

“*Phytolacca dodecandra* were *dominant dodecandra*” needs to be revised.

**Author’s Response:** corrected

**Similarity among community types in the Seqela Forest**

“To find the differences in species composition between the four community types, Sorensen's similarity coefficient (Ss) was calculated.” My concern here: I did not see this part in the method section.

**Author’s Response:** We have added the information in the method section of revised manuscript

Figures 3, 4 and 5 need to increase their resolutions.

**Author’s Response:** We have tried to clearly drown and describe the caption of the figure under the diagram and check the revised manuscript.

**References**

Please correct the font type “Alelign A , Teketay D, Yemshawy, & Sue Edwards (2007)”.

**Author’s Response:** The correction has been made in the revised manuscript

**Reviewer #2:** The following suggestions may help to improve the quality of the Ms. further. Overall, the title clearly reflects the contents. The introduction does establish the existing state of knowledge but needs minor revision as suggested. Authors should add the knowledge gap at the end of the introduction with proper citations. The set of measurements was comprehensive. Discussion part needs revision as suggested in the text.
The conclusion needs concreteness. Do not repeat the results in the conclusions. Figure 5 needs proper drawing (reduce the size appropriately).The citations need up-dated.
English needs improvement. I have made more corrections and suggestions directly in the manuscript. Please revise the paper accordingly. Please note that Introduction should be started with the general statement not by specific first. As it is an International paper and will be viewed by international audience not only by Ethiopian’s.

**Author’s Response:** Thank you for your efforts in reviewing the manuscript and providing valuable suggestions for improving the article. You have corrected many things of our manuscript. We have tried to clearly correct the manuscript and check the revised manuscript.
