# Supplementary material for: Woody species diversity, structure and community distribution along environmental gradients of Seqela Dry Afromontane forest in Northwestern Ethiopia
Source: PLoS One. 2025 Jan 17;20(1):e0313020. doi: 10.1371/journal.pone.0313020 (PMC11741620; doi:10.1371/journal.pone.0313020)
Supplement: Attachment — Submitted filename: PONE-D-24-05897R1.docx [file pone.0313020.s006.docx]

**Manuscript Number: PONE-D-24-05897R1**

**Woody species diversity, Vegetation structure and Plant community distribution along environmental gradients of Seqela Dry Afromontane Forest in Northwestern Ethiopia**

PLOS ONE

**General comments**

The authors have satisfactorily addressed the majority of my concerns and comments, although some minor ones still need to be addressed.

**Title**: It needs minor revision “Woody species diversity, structure, and community distribution along environmental gradients of Seqela Dry Afromontane Forest in Northwestern Ethiopia”

**Introduction**

In paragraph 2, replace “[25; 26]” with “[25, 26].

“… environmental factors [e.g. 27- 31]” please restate this statement. Additionally, use proper punctuation and writing techniques throughout your paper such as, replace “27- 31” with “27–31”.

**Methodology**

**Study area**

Paragraph 2 replace “*Eragrostis teff*” with “*Eragrostis tef*”.

**Data Analysis**

**Plant community classification**

##There is a mix of information herein: “agglomerative hierarchical cluster analysis”? Select either “agglomerative cluster analysis” or “hierarchical cluster analysis” with acceptable reason.

##The author did not give sufficient response for this question raised in the first draft.

**Results**

**Plant community types**

\## “Figure 3. Dendrogram”, here is my big question why not 2 types or 3 community types or else? Because the author did not show the value of the cophenetic correlation coefficient where they determined the cutree.

##The author did not give sufficient response for this question raised in the first round of review rather they replied very subjective suggestions.
