# Supplementary material for: Woody species diversity, structure and community distribution along environmental gradients of Seqela Dry Afromontane forest in Northwestern Ethiopia
Source: PLoS One. 2025 Jan 17;20(1):e0313020. doi: 10.1371/journal.pone.0313020 (PMC11741620; doi:10.1371/journal.pone.0313020)
Supplement: Attachment — Submitted filename: Point by point response to the Reviewer Comments__R2.docx [file pone.0313020.s007.docx]

**Manuscript Number: PONE-D-24-05897R2**

**Woody species diversity, Vegetation structure and Plant community distribution along environmental gradients of Seqela Dry Afromontane Forest in Northwestern Ethiopia**

PLOS ONE

**Article type:** Research Paper

We would like to thank the reviewers for their efforts in reviewing the manuscript and providing valuable suggestions for improving the article. All comments and suggestions were addressed. The changes are marked in yellow color as shown in the revised manuscript. We detailed response for each comment is provided below.

**Editor**

The authors have appropriately revised the manuscript and answered all the questions of the reviewers satisfactorily. I recommend the revised version of the manuscript be accepted for publication in PLOS ONE after minor revision.

**Author’s Response:** Thanks a lot for letting us revise our paper. Below are the responses to a reviewer one comments.

**Reviewer #1**:

1. **General comments**

The authors have satisfactorily addressed the majority of my concerns and comments, although some minor ones still need to be addressed.

**Author’s Response:** Thanks a lot for this very positive feedback.

2. **Title**: It needs minor revision “Woody species diversity, structure, and community distribution along environmental gradients of Seqela Dry Afromontane Forest in Northwestern Ethiopia”

**Author’s Response:** Thank you for this.

3. In paragraph 2, replace “[25; 26]” with “[25, 26].

“… environmental factors [e.g. 27- 31]” please restate this statement. Additionally, use proper punctuation and writing techniques throughout your paper such as, replace “27- 31” with “27–31”.

**Author’s Response:** The correction has been made in the revised manuscript.

4. **Study area**

Paragraph 2 replaces “*Eragrostis teff*” with “*Eragrostis tef*”.

**Author’s Response:** Thank you very much for your excellent observation. We have corrected in the revised version of the manuscript.

**Data Analysis**

**Plant community classification**

##There is a mix of information herein: “agglomerative hierarchical cluster analysis”? Select either “agglomerative cluster analysis” or “hierarchical cluster analysis” with acceptable reason.

##The author did not give sufficient response for this question raised in the first draft.

**Author’s Response:** Based on your previous comment, we have corrected it as “agglomerative hierarchical cluster analysis”. Let's clarify this comment.

Cluster analysis in ecology is a statistical method used to classify objects (such as species, communities, or individuals) into groups or clusters based on similarity. There are several types of cluster analysis techniques commonly used in ecology. Basically the methods of cluster analysis fall into **hierarchical and non-hierarchical (partitioning) techniques**.

1. **Hierarchical clustering**

- Hierarchical clustering is an assembling technique of vegetation stands that could be classified into groups, the processes being repeated at different levels to form a tree.
- There are two types of hierarchical clustering methods, namely **agglomerative and divisive**.

A. **Agglomerative hierarchical clustering:** It starts with each object as a separate cluster and then iteratively merges the closest clusters until only one cluster remains. called “**bottom up method**”, construct a tree-like hierarchy,
B. **Divisive Hierarchical Clustering :** Divisive hierarchical clustering method is the opposite of agglomerative classification in that the clustering start at the top and work downwards, beginning with the whole collection of plots, and dividing them to form successive subclasses and ultimately form a more or less similar groups. This method is not too common method for plant classification.

- Agglomerative Hierarchical Clustering is often preferred for plant community classification because it starts with individual data points and progressively groups them together based on similarity, mirroring the natural progression of plant community formation where individual plants come together to form larger groupings. This approach aligns well with the hierarchical structure often observed in plant communities, making it a suitable method for classification.

2. **Partitioning (non-hierarchical clustering):** Partitioning is a method of classifying vegetation stands in the same level, not in hierarchy ( eg. K- means clustering, Fuzzy clustering).

**Results**

**Plant community types**

\## “Figure 3. Dendrogram”, here is my big question why not 2 types or 3 community types or else? Because the author did not show the value of the cophenetic correlation coefficient where they determined the cutree. The author did not give sufficient response for this question raised in the first round of review rather they replied very subjective suggestions.

**Author’s Response:** Again, Let's clarify this comment. The cophenetic correlation coefficient can be used as some kind of measure of the goodness of fit of a particular dendrogram. Based on the agglomerative hierarchical cluster analysis performed in the study area, four plant community types were identified. The cophenetic correlation coefficient reached a value of 0.774, indicating a considerable degree of similarity in the classification. This information included in the revised manuscript.

Figure 3. Dendrogram”, here is my big question why not 2 types or 3 community types or else?

**Author’s Response:** we appreciate you raising this question. I (corresponding author) frequently inquire about this question when examining the students or reviewing manuscripts. Determining the number of groups in a cluster analysis is often the primary goal. Among the methods we used to decide the optimum cluster is **Elbo method**, finally we have got 4 plant community types.
