# Supplementary material for: Woody species diversity, structure and community distribution along environmental gradients of Seqela Dry Afromontane forest in Northwestern Ethiopia
Source: PLoS One. 2025 Jan 17;20(1):e0313020. doi: 10.1371/journal.pone.0313020 (PMC11741620; doi:10.1371/journal.pone.0313020)
Supplement: Attachment — Submitted filename: PONE-D-24-05897_R2.docx [file pone.0313020.s008.docx]

PLOS ONE

Woody species diversity,structure and community distribution along environmental gradientsof Seqela Dry Afromontane Forest in Northwestern Ethiopia

--Manuscript Draft--

| Manuscript Number: | PONE-D-24-05897R2 |
| --- | --- |
| Article Type: | Research Article |
| Full Title: | Woody species diversity,structure and community distribution along environmental gradientsof Seqela Dry Afromontane Forest in Northwestern Ethiopia |
| Short Title: | Plant community distribution |
| Corresponding Author: | Liyew Birhanu  Debre Markos University College of Natural and Computational Science Debremarkos, ETHIOPIA |
| Keywords: | Environmental gradients, plant community, Seqela forest, species diversity, vegetation structure |
| Abstract: | Dry evergreen Afromontane forests are severely threatened due to the expansion of agriculture, overgrazing by livestock, and ineffective management. The objective of this study was to investigate the floristic composition, vegetation structure, and classification of plant communities and assess the relationship between plant community types and environmental variables in the study forest. Systematic sampling was used to collect vegetation and environmental data from 52 (20 m x 20 m) (400 m2) plots established at every 200 m intervals along transect lines that were 100 m apart from each other following altitudinal gradient. Density, DBH, basal area, frequency, and importance value indices (IVI) of woody species were computed to characterize the vegetation structure of the forest. Agglomerative hierarchical cluster analysis and canonical Correspondence Analysis (CCA) with R software were used to identify plant communities and analyse the relationship between plant community types and environmental variables, respectively. A total of 68 woody plant species belonging to 63 genera and 44 families were identified. Four plant community types were identified: Albizia gummifera - Ekebergia capensis,Prunus africana - Croton macrostachyus, Vachelliaabyssinica - Dombeya torrida and Schefflera abyssinica - Teclea nobilis. The CCA results showed that the variation of species distribution and plant community formation were significantly (P < 0.005) related to altitude, organic matter, aspect, slope, and phosphorus. The total basal area and density of woody species were 27.4 m2 ha−1 and 1079.3 individual ha−1, respectively. The regeneration status assessment of the forest revealed a poor regeneration status, which was linked to the presence of multiple disturbance factors such as cutting and charcoal production signs. Therefore, it needs immediate conservation to ensure sustainable use and management of the forest. |
| Order of Authors: | Liyew Birhanu |
|  | Getaneh Moges, MSc. |
|  | Nigussie Amsalu, PhD |
| Response to Reviewers: | Manuscript Number: PONE-D-24-05897R2  Woody species diversity, Vegetation structure and Plant community distribution along environmental gradients of Seqela Dry Afromontane Forest in Northwestern Ethiopia PLOS ONE  Article type: Research Paper  We would like to thank the reviewers for their efforts in reviewing the manuscript and providing valuable suggestions for improving the article. All comments and suggestions were addressed. The changes are marked in yellow color as shown in the revised manuscript. We detailed response for each comment is provided below.  Editor  The authors have appropriately revised the manuscript and answered all the questions of the reviewers satisfactorily. I recommend the revised version of the manuscript be accepted for publication in PLOS ONE after minor revision.  Author’s Response: Thanks a lot for letting us revise our paper. Below are the |

*Powered by Editorial Manager® and ProduXion Manager® from Aries Systems Corporation*

|  | responses to a reviewer one comments. Reviewer #1:   1. General comments   The authors have satisfactorily addressed the majority of my concerns and comments, although some minor ones still need to be addressed.  Author’s Response: Thanks a lot for this very positive feedback.   2. Title: It needs minor revision “Woody species diversity, structure, and community distribution along environmental gradients of Seqela Dry Afromontane Forest in Northwestern Ethiopia”   Author’s Response: Thank you for this.   3. In paragraph 2, replace “[25; 26]” with “[25, 26].   “… environmental factors [e.g. 27- 31]” please restate this statement. Additionally, use proper punctuation and writing techniques throughout your paper such as, replace “27- 31” with “27–31”.  Author’s Response: The correction has been made in the revised manuscript.   4. Study area   Paragraph 2 replaces “Eragrostis teff” with “Eragrostis tef”.  Author’s Response: Thank you very much for your excellent observation. We have corrected in the revised version of the manuscript.  Data Analysis  Plant community classification  ##There is a mix of information herein: “agglomerative hierarchical cluster analysis”? Select either “agglomerative cluster analysis” or “hierarchical cluster analysis” with acceptable reason.  ##The author did not give sufficient response for this question raised in the first draft.  Author’s Response: Based on your previous comment, we have corrected it as “agglomerative hierarchical cluster analysis”. Let's clarify this comment.  Cluster analysis in ecology is a statistical method used to classify objects (such as species, communities, or individuals) into groups or clusters based on similarity. There are several types of cluster analysis techniques commonly used in ecology. Basically the methods of cluster analysis fall into hierarchical and non-hierarchical (partitioning) techniques.   1. Hierarchical clustering   Hierarchical clustering is an assembling technique of vegetation stands that could be classified into groups, the processes being repeated at different levels to form a tree. There are two types of hierarchical clustering methods, namely agglomerative and divisive.   A. Agglomerative hierarchical clustering: It starts with each object as a separate cluster and then iteratively merges the closest clusters until only one cluster remains. called “bottom up method”, construct a tree-like hierarchy, B. Divisive Hierarchical Clustering : Divisive hierarchical clustering method is the opposite of agglomerative classification in that the clustering start at the top and work downwards, beginning with the whole collection of plots, and dividing them to form successive subclasses and ultimately form a more or less similar groups. This method is not too common method for plant classification.   •Agglomerative Hierarchical Clustering is often preferred for plant community classification because it starts with individual data points and progressively groups them together based on similarity, mirroring the natural progression of plant community formation where individual plants come together to form larger groupings. This approach aligns well with the hierarchical structure often observed in plant communities, making it a suitable method for classification.  2.Partitioning (non-hierarchical clustering): Partitioning is a method of classifying vegetation stands in the same level, not in hierarchy ( eg. K- means clustering, Fuzzy clustering).  Results  Plant community types  ## “Figure 3. Dendrogram”, here is my big question why not 2 types or 3 community types or else? Because the author did not show the value of the cophenetic correlation coefficient where they determined the cutree. The author did not give sufficient response for this question raised in the first round of review rather they replied very subjective suggestions.  Author’s Response: Again, Let's clarify this comment. The cophenetic correlation coefficient can be used as some kind of measure of the goodness of fit of a particular |
| --- | --- |

*Powered by Editorial Manager® and ProduXion Manager® from Aries Systems Corporation*

|  | dendrogram. Based on the agglomerative hierarchical cluster analysis performed in the study area, four plant community types were identified. The cophenetic correlation coefficient reached a value of 0.774, indicating a considerable degree of similarity in the classification. This information included in the revised manuscript.  Figure 3. Dendrogram”, here is my big question why not 2 types or 3 community types or else?  Author’s Response: we appreciate you raising this question. I (corresponding author) frequently inquire about this question when examining the students or reviewing manuscripts. Determining the number of groups in a cluster analysis is often the primary goal. Among the methods we used to decide the optimum cluster is Elbo method, finally we have got 4 plant community types. |
| --- | --- |
| Additional Information: |  |
| Question | Response |
| Financial Disclosure  Enter a financial disclosure statement that describes the sources of funding for the work included in this submission. Review the [submission guidelines](http://journals.plos.org/plosone/s/submission-guidelines#loc-financial-disclosure-statement) for detailed requirements. View published research articles from [*PLOS ONE*](https://journals.plos.org/plosone/) for specific examples.  This statement is required for submission and will appear in the published article if the submission is accepted. Please make sure it is accurate. | The author(s) received no specific funding for this work. |
| Funded studies  Enter a statement with the following details:   - Initials of the authors who received each award - Grant numbers awarded to each author - The full name of each funder - URL of each funder website - Did the sponsors or funders play any role in the study design, data collection and analysis, decision to publish, or preparation of the manuscript? |  |
| Did you receive funding for this work? |  |
| Competing Interests  Use the instructions below to enter a competing interest statement for this submission. On behalf of all authors, disclose any [competing interests](http://journals.plos.org/plosone/s/competing-interests) that could be perceived to bias this work—acknowledging all financial support and any other relevant financial or non- | The authors declare that they have no competing interests. |

*Powered by Editorial Manager® and ProduXion Manager® from Aries Systems Corporation*

| financial competing interests.  This statement is required for submission and will appear in the published article if the submission is accepted. Please make sure it is accurate and that any funding sources listed in your Funding Information later in the submission form are also declared in your Financial Disclosure statement.  View published research articles from  [*PLOS ONE*](http://journals.plos.org/plosone/) for specific examples. |  |
| --- | --- |
| NO authors have competing interests  Enter: *The authors have declared that no competing interests exist.*  Authors with competing interests  Enter competing interest details beginning with this statement:  *I have read the journal's policy and the authors of this manuscript have the following competing interests: [insert competing interests here]* |  |
| * typeset |  |
| Ethics Statement  Enter an ethics statement for this submission. This statement is required if the study involved:   - Human participants - Human specimens or tissue - Vertebrate animals or cephalopods - Vertebrate embryos or tissues - Field research   Write "N/A" if the submission does not require an ethics statement.  General guidance is provided below. Consult the [submission guidelines](http://journals.plos.org/plosone/s/submission-guidelines#loc-guidelines-for-specific-study-types) for | "N/A" |

*Powered by Editorial Manager® and ProduXion Manager® from Aries Systems Corporation*

| detailed instructions. Make sure that all information entered here is included in the Methods section of the manuscript. |  |
| --- | --- |
| Format for specific study types  Human Subject Research (involving human participants and/or tissue)   - Give the name of the institutional review board or ethics committee that approved the study - Include the approval number and/or a statement indicating approval of this research - Indicate the form of consent obtained (written/oral) or the reason that consent was not obtained (e.g. the data were analyzed anonymously)   Animal Research (involving vertebrate animals, embryos or tissues)   - Provide the name of the Institutional Animal Care and Use Committee (IACUC) or other relevant ethics board that reviewed the study protocol, and indicate whether they approved this research or granted a formal waiver of ethical approval - Include an approval number if one was obtained - If the study involved *non-human primates*, add *additional details* about animal welfare and steps taken to ameliorate suffering - If anesthesia, euthanasia, or any kind of animal sacrifice is part of the study, include briefly which substances and/or methods were applied   Field Research  Include the following details if this study involves the collection of plant, animal, or other materials from a natural setting:   - Field permit number - Name of the institution or relevant body that granted permission |  |
|  |  |
| Data Availability  Authors are required to make all data underlying the findings described fully available, without restriction, and from the time of publication. PLOS allows rare | Yes - all data are fully available without restriction |

*Powered by Editorial Manager® and ProduXion Manager® from Aries Systems Corporation*

| exceptions to address legal and ethical concerns. See the [PLOS Data Policy](http://journals.plos.org/plosone/s/data-availability) and [FAQ](http://journals.plos.org/plosone/s/data-availability#loc-faqs-for-data-policy) for detailed information.  A Data Availability Statement describing where the data can be found is required at submission. Your answers to this question constitute the Data Availability Statement and will be published in the article, if accepted. |  |
| --- | --- |
| Important: Stating ‘data available on request from the author’ is not sufficient. If your data are only available upon request, select ‘No’ for the first question and explain your exceptional situation in the text box. |  |
| Do the authors confirm that all data underlying the findings described in their manuscript are fully available without restriction? |  |
| Describe where the data may be found in full sentences. If you are copying our sample text, replace any instances of XXX with the appropriate details.   - If the data are held or will be held in a public repository, include URLs, accession numbers or DOIs. If this information will only be available after acceptance, indicate this by ticking the box below. For example: *All XXX files are available from the XXX database (accession number(s) XXX, XXX.*). - If the data are all contained within the manuscript and/or Supporting Information files, enter the following: *All relevant data are within the manuscript and its Supporting Information files.* - If neither of these applies but you are able to provide details of access elsewhere, with or without limitations, please do so. For example:   *Data cannot be shared publicly because of [XXX]. Data are available from the XXX Institutional Data Access / Ethics Committee (contact via XXX) for researchers who meet the criteria for* | All relevant data are within the manuscript. |

*Powered by Editorial Manager® and ProduXion Manager® from Aries Systems Corporation*

| *access to confidential data.*  *The data underlying the results presented in the study are available from (include the name of the third party and contact information or URL).*   - This text is appropriate if the data are owned by a third party and authors do not have permission to share the data.   * typeset |  |
| --- | --- |
| Additional data availability information: |  |

*Powered by Editorial Manager® and ProduXion Manager® from Aries Systems Corporation*

Cover Letter	[Click here to access/download;Manuscript;Liyew PLOS](https://www2.cloud.editorialmanager.com/pone/download.aspx?id=35998631&guid=6855ef8f-f26a-42fc-a338-09760a25ba95&scheme=1)[<https://www2.cloud.editorialmanager.com/pone/download.aspx?id=35998631&guid=6855ef8f-f26a-42fc-a338-09760a25ba95&scheme=1> ONE_Revised_R2_ clean copy.docx](https://www2.cloud.editorialmanager.com/pone/download.aspx?id=35998631&guid=6855ef8f-f26a-42fc-a338-09760a25ba95&scheme=1)

### Woody species diversity, structure and community distribution along environmental gradients of Seqela Dry Afromontane Forest in Northwestern Ethiopia

**Liyew Birhanu^1^*, Getaneh Moges^1^, Nigussie Amsalu^1^**

^1^Department of Biology, Debre Markos University, P.O. Box: 269, Debre Markos, Ethiopia

*****Corresponding

Author: [liyewmtu@gmail.com](mailto:liyewmtu@gmail.com); [**liyew_birhanu@dmu.edu.et**](mailto:liyew_birhanu@dmu.edu.et)

## ABSTRACT

Dry evergreen Afromontane forests are severely threatened due to the expansion of agriculture, overgrazing by livestock, and ineffective management. The objective of this study was to investigate the floristic composition, vegetation structure, and plant communities and assess the relationship between plant community types and environmental variables in the study forest. Systematic sampling was used to collect vegetation and environmental data from 52 (20 m x 20 m) (400 m^2^) plots established at every 200 m interval along transect lines

100 m apart from each other following altitudinal gradient. Density, DBH, basal area, frequency, and importance value index (IVI) of woody species were computed to characterize the vegetation structure of the forest. Agglomerative hierarchical cluster analysis and canonical Correspondence Analysis (CCA) with R software were used to identify plant communities and analyse the relationship between plant community types and environmental variables, respectively. A total of 68 woody plant species belonging to 63 genera and 44 families were identified. Four plant community types were identified: *Albizia gummifera - Ekebergia capensis, Prunus africana - Croton macrostachyus*, *Vachellia abyssinica - Dombeya torrida* and *Schefflera abyssinica - Teclea nobilis*. The CCA results showed that the variation of species distribution and plant community formation were significantly (P < 0.005) related to altitude, organic matter, aspect, slope, and phosphorus. The total basal area and density of woody species were 27.4 m^2^ ha^−1^ and 1079.3 individuals ha^−1^, respectively. The regeneration status assessment of

the forest revealed a poor regeneration status, which was linked to the presence of multiple disturbance factors such as cutting and charcoal production signs. Therefore, it needs immediate conservation to ensure sustainable use and management of the forest.

**Key words:** Environmental gradients, plant community, Seqela forest, species diversity, vegetation structure,

# 1. Introduction

Forest ecosystems are important repositories of terrestrial biodiversity and play a key role in influencing socio-ecological and cultural attributes of human societies including livelihood activities of traditional societies living as well as associated to these forests [1–3]. These ecosystems are highly threatened by anthropogenic activities [4 – 6]. Poverty, population pressure, agricultural expansion/intensification and infrastructural development are the major threats to biodiversity and environment [7–9]. Altitude and latitude have gradients associated to climatic parameters [10-12] which influence the species distribution and their function [13,14]. The fragmentation of a population at an environmental gradient due to disturbance (agents that change the population structure and dynamics), be it anthropogenic or natural, may result in different population growth and vital rates among the forest ecosystems [15, 16]. However, in multispecific or monospecific communities there are cases in which the differentiation of demographic parameters through time is not apparent due to autopoiesis [17, 18], resilience or autogenic regulation [19].

Ethiopia's forests are essential natural resources that offer significant socioeconomic and cultural benefits to local communities [20]. Moreover, these forests play a crucial role in providing various ecosystem services, such as biodiversity conservation, carbon sequestration, and watershed management [21]. However, despite their vital importance, the forest cover in Ethiopia has faced significant threats due to rapid population growth and land use change [22]. According to a recent study [23], Ethiopia's forest cover has decreased by 16% between 1990 and 2020, despite the

revised definition of forests that includes woodlands. Deforestation continues to be a major concern in the country. The diverse environmental conditions in Ethiopia, including variations in altitude, climate, and soil, as well as human interactions, result in varying plant community distribution, structure, and composition [24].

The dry evergreen Afromontane forests in Ethiopia are facing significant threats due to agricultural expansion, overgrazing by livestock, and population growth [22, 20]. Understanding the distribution patterns of plant species and the factors influencing them is essential for effective biodiversity conservation [25, 26]. Several studies have examined the floristic composition, structure, and distribution patterns of plant communities along environmental gradients within the dry evergreen Afromontane forests [e.g. 27–31]. However, no scientific study was made to document woody species composition, structure, and plant community distribution along environmental gradients in the Seqela Dry Afromontane forest. Thus, this study was designed to address the following objectives.(1) to document the woody species composition of the Seqela forest; (2) to identify plant community types in the study area, (3) to identify the major environmental factors determining species distribution and community composition in the Seqela forest; (4) to assess the vegetation structure and regeneration status of Seqela forest. As a result, this study was conducted to fill the existing gap and provide essential data for the effective conservation and sustainable management of the studied forest [32].

## 2. MATERIALS AND METHODS

### Description of the Study Area

The study was carried out in the Seqela Forest, which is located in Northwestern Ethiopia's Quarit District of the West Gojjam Zone of the Amhara Regional State (Fig. 1). The district is 418 kilometres and 233 kilometres northwest of Addis Ababa and Bahir Dar city, respectively, in the northwest region of Ethiopia. Seqela Forest is categorised as dry evergreen Afromontane

forest [33]. The forest is located between 100°56'0'' and 100°57'30'' N and 370°28'0'' and 370°28'30'' E. Eutric, vertsols, a subset of vertisols, are the most common form of soil in the research area [34].

The 10-year climate data (2012-2021) for the Quarit station was obtained from the National Meteorological Services Agency. The average annual rainfall in the district is 1393 mm. The average mean minimum and maximum temperatures in the study area are 10°C and 30.70°C, respectively, with an average annual temperature of about 20.10°C (Figure 2). The main crop types in the district include cereal crops, oil crops, pulses, and vegetables. Cereal crops include barley (*Hordeum vulgare*), wheat (*Triticum sp.),* teff (*Eragrostis tef*), and maize (*Zea mays*). Oil crops include Niger seed (*Guizotia abyssinica*) and sunflower ( *Helianthus annus*). Pulses include faba bean (*Vicia faba*) and field pea (*Pisum sativum*). Vegetables include cabbage (*Brassica oleracea*), potatoes (*Solanum tuberosum*), garlic (*Allium sativum*), and onion (*Allium cepa*). Potatoes are the primary traded tuber crop [35]. Letters of consent were taken from Debre Markos University and Quarit District Agriculture and Rural Development Offices before data collection. Thus, we obtained permission to collect both vegetation and environmental data and conduct our research in the study area.

**Sampling design**

A reconnaissance study was conducted across the forest to determine the sampling plots for the collection of environmental and vegetation data, as well as to get an overview of the site characteristics. To collect data on vegetation and the environment, a systematic sampling method was applied. A total of 52 plots, 20 m x 20 m along transects following altitudinal gradient were laid down. The distance between two consecutive plots and transect lineswas 100 and 200 m apart in the forest, respectively. Five smaller subplots of 5 x 5 m^2^, four at the corner, and one at the center of the main plot, were established for seedling and sapling species data collection [36].

### Vegetation data collection

Trees and shrubs larger than 2.5 cm DBH were counted individually in each plot and all woody plant species recorded. For shrubs and trees larger than 2.5 cm in diameter, the diameter at breast height (DBH) was measured and recorded for plants with DBH >2.5 cm. Plants taller than 2.5 m were considered mature trees and shrubs t, whereas saplings between 1 and 2.5 m and seedlings up to 1 m tall. All collected plant specimens were pressed, dried and taken to the Herbarium of Debre Markos University, for taxonomic identification using the published books of Flora of Ethiopia and Eritrea. Finally, the presence-absence and cover abundance data were converted to the modified 1–9 Braun Blanquet scale [37].

### Environmental data collection

For every plot within the forest, environmental data such as altitude, longitude and latitude were captured using GPS. Each sample plot's slope was determined using a Suunto clinometer. Five locations, each sized 15 × 15 cm and with a depth of 0–30 cm, were used to gather soil samples from each main plot (four from each corner and one from the center) [38]. After combining these samples to create a composite sample, about 1 kg was used for examination/analysis. Before chemical tests, the soil samples were air dried, crushed, and sieved with a 2 mm screen size to remove organic waste and root particles.

Following standard protocols, measurements and analyses were carried out for pH, electrical conductivity (EC), cation exchange capacity (CEC), soil organic matter (SOM), total nitrogen (N), available phosphorus (P), and soil texture (sand, silt, and clay). The soil analysis was assessed at Debre Markos Soil Research and Fertility Improvement's soil laboratory [39].

### Data Analysis

**Plant community classification**

Using the estimated value of percent cover for each woody species, Agglomerative hierarchical cluster analysis was performed in this work to classify the vegetation into plant community types using the free statistical program R version 4.12 [40]. Based on the relative magnitude of their synoptic values, one or two main indicator tree or shrub species were chosen for identifying the community. A total of 64 plant species and 52 sample plots were used in the analysis.

### Diversity and similarity indices

Shannon-Wiener diversity index and Shannon’s evenness were computed to describe the species diversity of the study area [41].

 , where by

H' = Shannon diversity index

**s** = number of species,

p= proportion of individuals or abundance of the ith species expressed as a proportion of total cover in the sample; and *ln* = natural logarithm.

Shannon's evenness index (J) was also calculated using:

where H' = Shannon–Wiener Diversity Index; and H'max = lns, where s is the number of species in the sample.

### Species similarity analysis

So as to compare species composition similarity among community types, Sorensen’s similarity coefficient was calculated by the following formula [41].

Where, Ss = Sorensen’s similarity coefficient

a=number of species common to both samples /communities/study areas b =number of species in sample1, c=number of species in sample 2

### Ordination

A multivariate method called ordination is used to show interactions in a small-scale region between species, plots, and environmental variables [42]. Using SAS software, a preliminary analysis of the data was performed by computing Pearson's correlation coefficient to find significant correlations between environmental variables and remove auto-correlated variables from the ordination analysis. The present study used Canonical Correspondence Analysis (CCA) to look at the links between environmental variables and vegetation. Thirteen soil factors (pH, EC, total nitrogen, available phosphorus, CEC, and OM) as well as

52 sample plots, 64 species, and moisture content have been included in the analysis. To determine the effect of soil on the distribution of plant communities, an Adonis test was performed. Cover abundance values for plant species and information on significant environmental variables were shown in the CCA ordination plot.

### Structural data analysis

Density, basal area, frequency, IVI, and DBH class distributions were calculated for each woody plant species used for the description of vegetation structure. Thus, the following formulas were used to calculate the frequency, density, and basal area of woody species.

**=**(4)

(5)

With ( where	(6)

DBH is diameter at breast height

An importance value index (IVI) was computed for all woody species based on their relative density (RD), relative dominance (RDO), and relative frequency (RF). This index is used to determine the overall importance of each species in the forest system [41].

The Importance Value Index (IVI) for each woody species was computed using the following formula

Where, RDO= relative dominance, BA= Basal area, TBA= total basal area

(7)

(8)

𝑅𝑒𝑙𝑎𝑡𝑖𝑣𝑒 𝐹𝑟𝑒𝑞𝑢𝑒𝑦 =

𝑁𝑢𝑚𝑏𝑒𝑟 𝑜𝑓 𝑠𝑎𝑚𝑝𝑙𝑒 𝑝𝑙𝑜𝑡𝑠 𝑐𝑜𝑛𝑡𝑎𝑖𝑛𝑖𝑛𝑔 𝑎 𝑠𝑝𝑒𝑐𝑖𝑒𝑠

𝑆𝑎𝑚𝑝𝑙𝑒 𝑢𝑛𝑖𝑡𝑠 𝑓𝑜𝑟 𝑎𝑙𝑙 𝑠𝑝𝑒𝑐𝑖𝑒𝑠 𝑜𝑓 𝑡ℎ𝑒 𝑠𝑎𝑚𝑝𝑙𝑒

× 100 ( 9)

𝐼𝑉𝐼 = 𝑅𝑒𝑙𝑎𝑡𝑖𝑣𝑒 𝐷𝑒𝑛𝑠𝑖𝑡𝑦 + 𝑅𝑒𝑙𝑎𝑡𝑖𝑣𝑒 𝐷𝑜𝑚𝑖𝑛𝑎𝑛𝑐 + 𝑅𝑒𝑙𝑎𝑡𝑖𝑣𝑒 𝐹𝑟𝑒𝑞𝑢𝑒𝑛𝑐𝑦, (10)

### Regeneration status of the forest

The regeneration status of the forest was analysed by comparing the density ratios between seedlings and mature individuals, seedlings and saplings, and saplings and mature individuals*.* When seedlings are greater than saplings and saplings greater than adults, the status has good

regeneration, whereas seedlings are greater than or less than saplings and saplings less than adults; the status is poor regeneration, and the species survives only in the sapling stage (saplings may be less than or greater than adults), and a species is present only in adult form it considered as not regenerating [43].

3. **RESULTS**

### Woody species composition

In the study area, 68 species of woody plants were identified, belonging to 44 families and 63 genera. Out of them, 64 species were found inside the plots, and 4 species—*Maytenus arbutifolia, Ficus sur, Lippia adoensis, and Combretum molle* were found outside the plot (Appendix 1). Of the 64 species of woody plants that have been found, woody lianas make up the least represented growth form, accounting for 10.94% (7 in number) of the species. Of these, trees make up 48.44% (31 in number), and shrubs bring up 40.62% (26 in number) (Appendix 1).

**Plant community types**

Based on the agglomerative hierarchical cluster analysis performed in the study area, four plant community types were identified. The cophenetic correlation coefficient reached a value of 0.774, indicating a considerable degree of similarity in the classification (Figure 3). These communities were grouped as follows: *Prunus africana - Croton macrostachyus; Albizia gummifera – Ekebergia capensis; Vachellia abyssinica - Dombeya torrida; Schefflera abyssinica - Teclea nobilis*

***Albizia gummifera- Ekebergia capensis* Community Type I**

This community type was distributed between altitudinal ranges of 2314 –2520 m.a.s.l. The dominant tree species are *Albizia gummifera, Ekebergia capensis, Croton macrostachyus, Erythrina brucei, Pittosporum viridiflorum* and *Olea europaea* subsp. *cuspidata.* The shrub species *such as Vernonia amygdalina, Maesa lanceolata, Maytenus addat, Clutia abyssinica, Rosa abyssinica, Vernonia myriantha and Bridelia micrantha* were highly dominant. The most

dominant woody Lianas included in this community were *Zehneria scabra* and *Periploca linearifolia.* This community type is represented by 52 species, being the richest in a number of species among the four community types.

***Prunus africana- Croton macrostachyus* Community Type II**

The altitudinal range of this community lies between of 2378 – 2441 m.a.s.l. The most dominant tree species were *Prunus africana, Olinia rochetiana, Apodytes dimidiata* and *Croton macrostachyus.* The shrub layer is dominated by *Osyris quadripartita, Calpurnia aurea, Asparagus africanus, Bersama abyssinica* and *Rytigynia neglecta*. Woody liana species such as *Jasminum abyssinicum, Urera hypselodendron, Clematis longicauda* and *Phytolacca dodecandra* were *dominant.* This community is comprised of 34 species.

***Vachellia abyssinica -Dombeya torrid* Community Type III**

This community type was located between the altitudinal ranges of 2377 – 2577 m.a.s.l. The dominant plant species from this community were *Vachellia abyssinica*, *Dombeya torrida, Allophylus abyssinica, Maytenus obscura, Myrica salicifolia* and *Juniperus procera from* the tree layer and *Dovyalis abyssinica, Capparis tomentosa, Rubus steudneri, Rumex nervosus* and *Otostegia integrifolia* from shrub layer. This community is comprised of 48 species.

***Schefflera abyssinica - Teclea nobilis* CommunityType IV**

This community type was located between altitudinal ranges of 2335 – 2432 m.a.s.l. The dominant plant species in the community were *Schefflera abyssinica, Teclea nobilis, Euphorbia abyssinica, Dracaena steudneri, Solanecio gigas, Laggera tomentosa, Rhus glutinosa* and *Discopodium penninervium* from tree and shrub layer. This community is comprised of 27 species.

### Species richness, evenness, and diversity of community types in the Seqela forest

We calculated the Shannon-Wiener diversity index for each of the four types of plant communities. As a result, according to Shannon's diversity, community 1 had the greatest species diversity and richness, followed by community 3 community 4 had the lowest. Conversely, communities 1 and 2 were followed in order of evenness value, with community 3 having the greatest value. Among all types of communities, Community 4 had the lowest evenness scoring (Table 2).

### Similarity among community types in the Seqela forest

To find the differences in species composition between the four community types, Sorensen's similarity coefficient (Ss) was calculated. Among all communities, the overall similarity coefficient varies from 31 to 45%. Communities 1 and 2 had the next-most similarity (43%) after C1 and C3 (45%). Relatively little similarity (31%) was found between Communities 3 and 4. (Table 3).

### Relationship between plant community types and environment variables

The gradient length of 3.69 is greater than 3, as shown by the DCA used to choose the optimal ordination technique. As a result, CCA was accepted as an adequate ordination for examining the interactions between the environmental variables and the vegetation. The longest DCA axis for the Sekela forest data set in the present study was 3.69, indicating the heterogeneity of the data as presented in (Table 4). To evaluate the connections between community type and environmental variables, a study using the unimodal response model is suggested. The Canonical correspondence analysis (CCA) ordination method is employed in this analysis.

### CCA Ordination

Thirteen environmental factors were taken into consideration in the ordination analysis: aspect, pH, phosphorus, sand, clay, silt, slope, nitrogen, moisture content, altitude, and Cation Exchange Capacity. As Table 5 and Figure 4 indicate, the only factors that significantly (p < 0.05) correlated with species compositions and their distributions were altitude, OM, slope, aspect, and phosphorus. The canonical correlation analysis (CCA) diagram showed that aspect, av P, and OM were the main variables related to the first axis, and altitude and slope were the major factors associated with the second axis (Figure 4, Table 6). The first and second axes' respective eigenvalues were 0.34 and 0.22.

For the first and second axes, there was heterogeneity in patterns of plant species distribution and plant community formation of 41% and 22.9%, respectively. The results showed that the first two axes accounted for 63.9% of the variation in patterns of plant species distribution and plant community formation. Thus, it was sufficient to reflect the relationship between species and environmental factors using the first two axes.

### Vegetation structure

The distribution of woody plant species in various DBH classes was analyzed and classified into five categories: DBH classes 1 = 2.5-10 cm, 2 = 10.1-20 cm, 3 = 20.1-30 cm, 4 = 30.1-40 cm, and 5 = > 40.1 cm. The first DBH class (2.5-10 cm) had a greater density of woody individuals; 176 of them made up 28.8% of the total. 235 individuals, representing 24.5% of the total, were in the second DBH class (10.1–20 cm) density distribution. The density distributions for the remaining four classes were, in order, 177 (18.5%), 119 (12.4%), 72 (7.5%), and 80 (8.3%). From lower to higher DBH classes, the density distribution of woody individuals showed a declining trend.

In the Seqela natural forest, the overall density of woody species per hectare was 1079.3. *Croton macrostachyus* (88.46 individuals per hectare) had the highest density, followed *by Calpurnia aurea* (68.27 individuals per hectare), *Albizia gummifera* (70.19 individuals per hectare), and *Olinia rochetiana* (78.37 individuals per hectare). *Albizia gummifera* (51.92%), *Croton macrostachyus* (44.23%), *Olinia rochetiana*, and *Teclea nobilis* (36.54%) were the most common woody species in the Seqela natural forest. *Erythrina brucei*, *Juniperus procera*, *Hagenia abyssinica*, *Myrsine africana*, *Laggera tomentosa*, and *Rumex nervosus* were the least common woody species, occurring in 1.92% of cases each. The total basal area of woody plant species with a DBH greater than 2.5 cm was 27.4 m^2^ per hectare. The basal area of individual plant species increased as their DBH class increased, but their number decreased. Species such as *Erythrina brucei* (6.27 m2/ha), *Prunus africana* (3.67 m2/ha), *Vachellia abyssinica* (3.55 m2/ ha), and *Apodytes dimidiate* (2.59 m2/ha) and *Schefflera abyssinica* (2.04 m2/ha) contributed the largest basal area (Table 8).

### Importance Value Index (IVI) of Species

*Erythrina brucei* (IVI = 11.24), *Prunus africana* (8.68), Croton *macrostachyus* (IVI = 7.38), *Albizia gummifera* (IVI = 9.03), and *Vachellia abyssinica* (7.02) were the most dominant and ecologically significant species in the Seqela natural forest. Conversely, species like *Vernonia amygdalina*, *Juniperus procera*, *Hagenia abyssinica*, and *Dracaena steudneri* had lower IVI values (less than 1) (Table 9).

### Regeneration status of woody plants in Seqela forest

The regeneration status of selected woody plant species in the Seqela forest was assessed to predict the forest's future fate. The regeneration status of the Seqela forest was classified as poor regeneration, as seedlings (44.7 individuals/ha) < saplings (68.3 individuals/ha) < mature trees (461.1 individuals/ha), indicating a J-shaped pattern with a small number of seedlings and

saplings compared to mature trees (Figure 5). Woody plant species such as *Schefflera abyssinica*, *Apodytes dimidiata*, *Erythrina brucei*, *Hagenia abyssinica*, *Juniperus procera, Myrica salicifolia*, and *Prunus africana* were not represented by both seedling and sapling stages. *Pittosporum viridiflorum*, *Vachellia abyssinica*, *Albizia gummifera*, *Buddleja polystachya, Clausena anisata*, *Croton macrostachyus*, *Dombeya torrida*, *Dovyalis abyssinica*, *Euphorbia abyssinica*, *Olea europaea* subsp. *cuspidata*, *Olinia rochetiana*, *Osyris quadripartita*, *Rhamnus prinoides, Rytigynia neglecta*, *Teclea nobilis*, and *Vernonia amygdalina* exhibited fair regeneration status. Conversely, woody species such as *Allophylus abyssinicus*, *Buddleja davidii, Discopodium penninervium*, *Dracaena steudneri*, *Ekebergia capensis*, *Grewia ferruginea*, *Maytenus obscura*, and *Rhus vulgaris* showed poor regeneration status.

## 4. DISCUSSION

### Woody species composition

In the Seqela forest, we found 68 woody species. This lower than other studies conducted in other dry Afromontane forests of Ethiopia, such as those conducted in the Zege Peninsula forest [44] with 113 species, Tara Gedam and Abebaye forests [45] with 143 species, and the forest patches in the Dega Damot district [30] with 106 woody species, the study area showed lower species richness in woody species. The study area's species richness was higher than that of other dry Afromontane forests in Ethiopia, like the 64-species Denkoro Forest in Wello [46], the 50-species Zengena Forest [47], the 66-species Kuandisha Forest [48], and the 57-species Amoro Forest [49].

Fabaceae, Euphorbiaceae, and Rosaceae were among the plant families that were the most common in the study area. The order of families in terms of species richness is consistent with previous studies on dry evergreen Afromontane forests, which indicated the dominance of Asteraceae [29, 50–51]. Similarly, [48] reported that the most frequent family in the Kuandisha dry evergreen Afromontane forest fragment was Fabaceae. According to [52], the two most

prominent plant families in the flora of Ethiopia and Eritrea are the Asteraceae and Fabaceae. In Seqela Forest, the dominance of Asteraceae and Fabaceae may suggest that disturbances have occurred in the forest. Asteraceae plants are typically ruderal and thrive best in open, disturbed soil [31]. According to [31], the ability of Fabaceae to fix atmospheric nitrogen helps them to survive on soils deficient in nutrients, which accounts for their success in taking over disturbed habitats.

### Plant communities

In the Seqela forest, four plant communities were identified based on the findings of the hierarchical cluster analysis. However, species diversity and richness differed among plant communities. The differences in species composition along the communities may be due to the variation in physico-chemical properties of soil, topography, climate, weathering processes, and microbial activities [53–55] and several other biotic and abiotic factors [14]. For instance, communities 1 and 3 exhibited the highest species richness and diversity. Conversely, community 4 had the lowest species richness and diversity compared to the other communities. The reason for the high species diversity and richness in communities 1 and 3 may be due to the highest altitudinal ranges in which these communities are found, ranging from 2378 to 2441 and 2377 to 2577 m.a.s.l., respectively [39]. Additionally, community 4 was the most disturbed community, likely due to agricultural expansion and overgrazing by livestock.

### Relationship between plant community types and environment variables

The topography (aspect, slope, and altitude) and soil variables combined together to shape the distribution of plant communities in the Seqela forest. The main environmental factors that explained variations in plant species distribution and patterns of plant community formation in the research area were aspect, slope, altitude, OM, and av P, according to the CCA results. According to [56], aspect was shown to be the most significant environmental variable in determining vegetation variation. This could be attributed to variations in incoming solar radiation that impact

temperature and moisture levels. Plant growth and the creation of plant communities are strongly influenced by the temperature and moisture of the soil. Slope and altitude were the primary factors influencing vegetation distribution, according to similar studies conducted in other dry Afromontane forests in Ethiopia [57,49]. A similar study by [30] also confirms that organic matter and av-P were the most important constraining variables in plant community formation of the study area.

### Vegetation structure

The woody plant species distribution in Seqela Forest showed an inverted J-shape pattern, suggesting that small to medium-sized DBH trees dominated the forest rather than large-sized trees. This could be because local communities used these species for various purposes.

The total density of woody species in the Seqela forest was 1079.3 individuals per hectare. This was less than the results of studies conducted by [44] in the Zegie Peninsula Forest (3318 individuals per hectare), Berhanu et al. (2016) in the Kundasha Forest (3086 individuals per hectare), and [48] in the Amoro Forest (2860 individuals per hectare). The density was also higher than other forests, such as Wanzaye natural forest (498) [58]. The degree of anthropogenic interference in various forests may account for this variation in density. In the research area, species with the highest density values were *Clutia abyssinica*, *Croton macrostachyu*s, *Albizia gummifera*, *Olinia rochetiana*, and *Calpurnia aurea*. This might be because the species is small, resulting to a greater total count within a given plot size and a fairly excellent ability for regeneration in dry situations under shade. On the other hand, low-density species may be associated with increased grazing and tree and shrub cutting for charcoal and firewood [59]. The most common species in Seqela woodland were *Albizia gummifera*, *Croton macrostachyus*, *Olinia rochetiana*, *Urera hypselodendron*, and *Teclea nobilis*. These species were pioneer species, as reported by [27] *Albizia gummifera* and *C. macrostachyus* were also the most frequent species in Tara Gedam and Abebaye forests, respectively [45]. The total basal area of the Seqela forest was

27.4 m^2^ per hectare, and the basal area of individual plant species increased as their DBH class increased but their number decreased. The study area had relatively less total basal area than some dry Afromontane forests in Ethiopia, such as Tara Gedam Forest and Abebaye forests [45] and Wof washa forest [27], and Dega Damot district forest patches [39]. On the other hand, the basal area was greater than other forests, such as Amoro forest [49] and Kundasha forest [48]. The total basal area of the study area was comparable to that of the Zengena forest [60]. Species such as *Erythrina brucei*, *Prunus africana*, *Acacia abyssinica, Apodytes dimidiate*, *Albizia gummifera*, and *Schefflera abyssinica* had high basal area due to their large size, even though they had low density. The Importance Value Index (IVI) value was an important parameter used to determine the significance of species in a given ecosystem for conservation purposes [61]. Species with lower IVI values required high conservation efforts [60], while those with higher IVI values needed monitoring and management [59].

### Regeneration status

The regeneration status of natural vegetation is considered poor regeneration if seedlings are greater than or less than saplings and saplings are less than adults [43]. In the case of the Seqela forest, the regeneration status was considered as poor regeneration since seedlings (44.7 individuals per hectare) < saplings (68.3 individuals per hectare) < mature trees (461.1 individuals per hectare). The possible reasons for the existence of poor regeneration status in the forest might be grazing intensity and cutting of young plants for various purposes.

## 5. CONCLUSION

The woody species composition of the Seqela forest indicates a good level of woody species richness in the area. Four community types, including *Albizia gummifera - Ekebergia capensis*, *Prunus Africana* and *Croton macrostachyus*, *Vachellia abyssinica* and *Dombeya torrida, - Schefflera abyssinica - Teclea nobilis,* were identified. Canonical correspondence analysis (CCA)

showed that aspect, altitude, slope, OM, and P were the primary factors of plant distribution patterns and plant community types. This suggests that the distribution of plant species and the formation of plant communities are heavily reliant on these environmental conditions. Species with high densities at lower DBH classes were characteristic of the general vegetation structure of Seqela forest; this may be because shrubby species dominated. The assessment of the forest's regeneration status showed a poor degree of regeneration, which was associated with several disturbance causes such as cutting and signs of charcoal production. As a result of these disturbances, some plant species had no seedlings or saplings, while others had both seedlings and saplings. Species like *Schefflera abyssinica*, *Apodytes dimidiata*, *Erythrina brucei, Hagenia abyssinica*, *Juniperus procera*, *Myrica salicifolia*, and *Prunus africana* were among those with no seedlings and saplings.

### Data Availability

All relevant data are within the manuscript.

**Conflicts of interest**

The authors declare that they have no competing interests

### Acknowledgments

Agriculture and Rural Development Office of the Quarit District as well as to the respective chairpersons is also highly acknowledged.

### Supplementary Materials

Appendix 1: List of woody plant species collected from Seqela forest Quarit District

**References**

1. Hermann, T.M.. Indigenous knowledge and management of Araucaria araucana forest in the Chilean Andes: Implication for native forest conservation. Biological Conservation. 2006; 15: 647-662.
2. Karki H, Kiran Bargali, S.S. Bargali, Vibhuti and Y.S. Rawat. Plant diversity, regeneration status and standing biomass under varied degree of disturbances in temperate mixed oak- conifer forest, Kumaun Himalaya. International Journal of Ecology and Environmental Sciences. 2017;43 (4): 331-345.
3. Baboo B, R Sagar, SS Bargali and H Verma. Tree species composition, regeneration and diversity within the protected area of Indian dry tropical forest. Tropical Ecology. 2017;58(3): 409-423.
4. Htun, N. Z., N. Mizoue & S. Yoshida.. Tree species composition and diversity at different levels of disturbance in Popa Mountain Park, Myanmar. Biotropica. 2011; 43: 597–603.
5. Bisht S, SS Bargali, K Bargali, GS Rawat, YS Rawat and A Fartyal . Influence of Anthropogenic Activities on Forest Carbon Stocks-A Case Study from Gori Valley, Western Himalaya. Sustainability.2022, 14:16918 DOI: 10.3390/su142416918
6. Bisht S, GS Rawat, SS Bargali, YS Rawat and A Mehta. 2023. Forest vegetation response to anthropogenic pressures: a case study from Askot Wildlife Sanctuary, Western Himalaya.	Environment,	Development	and	Sustainability.2023; <https://doi.org/10.1007/s10668-023-03130-2>
7. Davidar, P., S. Sahoo, P. C. Mammen, P. Acharya, J. P. Puyravaud, M. Arjunan, J. P. Garrigues & K. Roessingh. 2010. Assessing the extent and causes of forest degradation in India: Where do we stand? Biological Conservation 143: 2937–2944.
8. Awasthi P, K Bargali SS Bargali and MK Jhariya. Structure and Functioning of Coriaria nepalensis Wall dominated Shrublands in degraded hills of Kumaun Himalaya. I. Dry Matter Dynamics. Land Degradation & Development.2022; 33(9): 1474-1494. <https://doi.org/10.1002/ldr.4235>
9. Pandey R, SS Bargali, K Bargali, VC Pandey.Temporal variability in fine root dynamics in relation to tree girth size in sub-tropical Shorea robusta forests. Land Degradation & Development. 2023;34(5): 1522-1537. <https://doi.org/10.1002/ldr.4550>
10. Arriaga L, Espinoza JM, Aguilar C, Martínez E, Gómez L, Loa E. Regiones terrestres prioritarias de México. Comisión Nacional para el Conocimiento y uso de la Biodiversidad.CONBIO. 2000; México.
11. Fartyal A, K Bargali and SS Bargali.The effect of different slope aspects on plant diversity and soil characteristics in a temperate grassland of Kumaun Himalaya. Vegetos. 2023; <https://doi.org/10.1007/s42535-023-00609-1>
12. Khatri K, B Negi, K Bargali and SS Bargali. Trait plasticity: a key attribute in the invasion success of Ageratina adenophora in different forest types of Kumaun Himalaya, India. Environment, Development and Sustainability. 2023a; DOI: 10.1007/s10668-023-03529-x
13. Manral V, K Bargali, SS Bargali MK Jhariya and K Padalia .Relationships between soil and microbial biomass properties and annual flux of nutrients in Central Himalayan forests, India. Land Degradation & Development.2022; 33 (12): 2014-2025. [https://doi.org/10.1002/ldr.4283.](https://doi.org/10.1002/ldr.4283)
14. Pandey R, SS Bargali, K Bargali, H Karki, and RK Chaturvedi . Dynamics of nitrogen mineralization and fine root decomposition in sub-tropical Shorea robusta Gaertner f. forests of Central Himalaya, India. Science of The Total Environment.2024; 921, <https://doi.org/10.1016/j.scitotenv.2024.170896>
15. Nordstrom SW, Dykstra AB, Wagenius S. Fires slow population declines of a long-lived prairie plant through multiple vital rates. Oecologia.2021;196:679•691, [https://doi.org/10.1007/s00442-021-04955-2.](https://doi.org/10.1007/s00442-021-04955-2)
16. Mourya NR, K Bargali, S.S. Bargali .Effect of Coriaria nepalensis Wall. colonization in a mixed conifer forest of Indian Central Himalaya. Journal of Forestry Research. 2019. ;30 (1), 305-317. DOI: 10.1007/s11676-018-0613-x.
17. Romo-González T, Larralde C, Puga-Olguín A, Vargas-Madrazo E.Hystory, landscapes, metaphors and ghosts around the concelt of specificity. Transdisciplinary Journal of Engineering & Science. 2021. 12, DOI: <https://doi.org/10.22545/2021/00158>.
18. Mackey B, Morgan E, Keith H.Evaluating forest landscape management for ecosystem integrity. Landscape Research, DOI: 10.1080/01426397.2023.2284938.
19. Muñoz R, Bongers F, Rozendaal DMA, González EJ, Dupuy JM, Meave JA. Autogenic regulation and resilience in tropical dry forest. Journal of Ecology.2021 109:3295• 3307,
20. Meragiaw, M, Woldu, Z, Martinsen, V. Floristic composition and structure of the Kibate Forest along environmental gradients in Wonchi, Southwestern Ethiopia. *J. For. Res.* 2021;**32**, 2669–2682, <https://doi.org/10.1007/s11676-021-01305-z>
21. Fekadu Gurmessa, Bikila Warkineh, Teshome Soromessa & Sebsebe Demissew). Species diversity and plant community distribution along environmental gradient in Tulu Lafto Forest, western Ethiopia. Phytocoenologia 2022
22. Williams, S.D., J.L. Vivero, S. Spawls, Aneh S. & Ensermu K. Ethiopian Highlands. In: Mittermeier, R.A., P. Robles-Gil, M. Hoffmann, J.D. Pilgrim, T.M. Brooks, C.G. Mittermeier & G. Fonseca (eds.), Hotspots Revisited: Earth´s Biologically Richest and Most Endangered Ecoregions, 2004; pp. 262-273. CEMEX, Mexico city, Mexico.
23. FAO, *Global Forest Resources Assessment: Main report* (Food and Agriculture Organization of the United Nations, 2020; Rome
24. Yemata G, Haregewoien G. Floristic composition, structure and regeneration status of woody plant species in Northwest Ethiopia,Trees, Forests and People. 2022;Volume 9,https://doi.org/10.1016/j.tfp.2022.100291
25. Brooks TM, Mittermeier RA, Mittermeier CG. Habitat loss and extinction in the hotspots of biodiversity. Conserv Biol 16:909–923
26. Magurran AE. Measuring biological diversity. Blackwell Science. 2004 , ltd, Malden, p 132
27. Bekele T. Vegetation ecology of remnant Afromontane forests on the central plateau of Shewa, Ethiopia. Sv. växtgeografiska sällsk. 1993
28. Teketay D, Bekele T.Floristic composition of Wof‐ Washa natural forest, Central Ethiopia: Implications for the conservation of biodiversity. Feddes Repertorium. 1995 ; 106 (1‐ 5):127–47.
29. Yineger H, Kelbessa E, Bekele T, Lulekal E. Floristic composition and structure of the dry Afromontane forest at Bale Mountains National Park, Ethiopia. SINET: Ethiopian Journal of Science. 2008; 31(2):103–20.
30. Birhanu L, BekeleT., Binyam Tesfaw, and Sebsebe Demissew. Relationships between topographic factors soil and plant communities in a dry Afromontane forest patches of North western Ethiopia. PLOS ONE, 2021; 16:1–18.
31. Dagne Y, Birhanu L. Floristic composition and plant community distribution along environmental gradients in Guard dry Afromontane forest of Northwestern Ethiopia. BMC Ecol Evol. 2023; 28;23(1):43. doi: 10.1186/s12862-023-02154-6.
32. Girmay, M., Bekele, T., Demissew, S. *et al.* Ecological and floristic study of Hirmi woodland vegetation in Tigray Region, Northern Ethiopia. *Ecol Process.2020;* **9**, 53 <https://doi.org/10.1186/s13717-020-00257-2>
33. Friis, I., Demissew S and van Breugel, P. Atlas of the potential vegetation of Ethiopia. The royal Danish Academy of Sciences and Letters.2010; Copenhagen,DK

| 34. Tassew | A, | Teffera | B, | Jafar | E, | Ashagrie | E, | Mulugeta | G, |
| --- | --- | --- | --- | --- | --- | --- | --- | --- | --- |
| Tesfaye | G, | Adimasu | S, | Limenih |  | S, Genet | T, | Yihenew | G. |

Selassie andLeyew Z. Comprehensive study on research, development and management interventions in Birr sub-watershed, Quarit district, West Gojjam zone, Amhara national regional state. 2011.

35. Gebey T and Mekuriaw Z. Diagnosis and intervention plans for west Gojjam zone, Amhara Region, Ethiopia. 2013.
36. Masresha G, Asnakew M, Chekole G, Melese G. Comparative study on floristic composition vegetation structure and regeneration status of woody forests in case of GondTeklehaimaot and Arsema Monastery Forest, Department of Biology, College of Natural and Computational Sciences, University of Gondar, Ethiopia.2019.
37. Van der Maarel, E. Transformation of Cover abundance values in phytosociology and its effects on community similarity. *Vegetation.1979;* ***3*9**:97-114
38. Tadesse G, Bekele T, Demissew S. Dryland woody vegetation along an altitudinal gradient on the eastern escarpment of Wolo, Ethiopia. SINET: Ethiop J Sci 2008; 31:43–54
39. Birhanu L. Ecological Study of the Vegetation and Land Use/ Land Cover Change analysis in Dega Damot District, West Gojjam Zone, Amhara Region, NorthwesternEthiopia PhD thesis ; Addis Ababa University, Ethiopia. 2020
40. R Development Core Team. R: A Language and Environment for Statistical Computing. A Programming Environment for Data Analysis and Graphics. Version 4.12. 2022
41. Kent M. Vegetation Description and Data Analysis a Practical Approach 2nd Eds. Blackwell Publishing, UK.
42. McCune B, Grace J, Urban D. Analysis of ecological communities. Gleneden Beach, OR: MjM software design. 2002.
43. Dhaulkhandi, M., Dobhal, A., Bhatt, S. and Kumar, M. Community structure and regeneration potential of natural forest site in Gangotri , India. J Basic Appli Sci.2008.
44. Alelign A , Teketay D, Yemshawy, & Sue Edwards .Diversity and status of regeneration of woody plants on the peninsula of Zegie, northwestern Ethiopia**.***Tropical Ecology.2007.* 48(1)**:** 37-49, 2007 ISSN 0564-3295.
45. Zegeye H, Teketay D and Kelbessa K. Diversity and regeneration status of woody species in Tara Gedam and Abebaye Forests, north-westernEthiopia. Journal of Forestry Research.2011; 22 (3):315–328
46. Ayalew A. A Floristic composition and structural analysis of Denkoro Forest, South Wello.M.Sc. Thesis. Addis Ababa University.2003.
47. Tadele D, Lulekal E, Damtie D and Assefa A. Floristic diversity and regeneration status of woody plants in Zengena forest, a remnant montane forest patch in northwestern Ethiopia. Journal of forestry research.2014; 25 (2):329–336.
48. Berhanu A, Demissew S, Woldu Z and Didita M. Woody species composition andstructure of KuandishaAfromontane forest fragment in northwestern Ethiopia.Journal of Forestry Research.2016; 28: 343-355.
49. Birhanu L, Bekele T and Demissew S . Woody species composition and structure of AmoroForest in West Gojjam Zone, Northwestern Ethiopia. *Journal of ecology andthe natural environment. 2018;***10** (4): 53-64.
50. Tilahun A, Soromessa T, Kelbessa E, and Dibaba A. Floristic composition and community analysis	of	Menagesha	Amba	Mariam	Forest (Egdu Forest) in the central plateau of Shewa, Ethiopia.Ethiopian Journal of Biological Sciences. 2011; **10** (2): 111-136.
51. Lulekal E. Plant diversity and ethnobotanical study of medicinal plants in Ankober District, North ShewaZone of Amhara Region, Ethiopia. Ph.D. Dissertation, Addis Ababa University. 312p. 2014.
52. Tadesse M. Asteraceae (Compositae). In: Hedberg, I., Friis, I., Edwards, S., (eds). Flora of Ethiopia and Eritrea. Vol. 4(1). The National Herbarium, AAU, Addis Ababa & Uppsala.2004.
53. Paudel, S., Sah, J.P. Physico chemical characters of soil in tropical soil (Shorea robusta Gaertn.) forests in eastern Nepal. Himal. J. Sci. 2003; 1 (2), 107–110.
54. Padalia K, SS Bargali, K Bargali and V Manral. Soil microbial biomass phosphorus under different land use systems. Tropical Ecology 63:30-48.2022; <https://doi.org/10.1007/s42965-021-00184-z>
55. Manral V, K Bargali, SS Bargali and C Shahi. Changes in soil biochemical properties following replacement of Banj oak forest with Chir pine in Central Himalaya, India. Ecological Processes.2020; 9:30 <https://doi.org/10.1186/s13717-020-00235-8>
56. Paudel S and O. R. Vetaas. “Effects of topography and land use on woody plant species composition and beta diversity in an arid Trans-Himalayan landscape, Nepal,” *Journal of Mountain Science*, 2014; vol. 11, no. 5, pp. 1112–1122
57. Shiferaw W, Bekele T, Demissew S. Anthropogenic effects on floristic composition, diversity	and	regeneration	potential	of	the	Debre	libanos Monastery forest patch, central Ethiopia. J For Res.2018; ;30(6):2151–61
58. Getnet A. Woody Species Composition, Diversity and Vegetation Structure of Dry Afromontane Forest, Ethiopia.*Journal of Agriculture and Ecology Research International. 2018;* ***16***(3): 1-20.
59. Birhanu L, Bekele T, Tesfaw B, Demissew S. Soil seed bank composition and aboveground vegetation in dry Afromontane forest patches of Northwestern Ethiopia,Trees, Forests and People,2022; Volume 9, <https://doi.org/10.1016/j.tfp.2022.100292>.
60. Tadele D, Lulekal E, Damtie D and Assefa A. Floristic diversity and regeneration status of woody plants in Zengena forest, a remnant montane forest patch in northwestern Ethiopia. Journal of forestry research.2014; 25 (2):329–336.
61. Lamprecht, H. Tropical forest ecosystems and their tree species. Silviculture in Tropics, Possibilities, and Methods for Their Long-Term Utilization. Institute of for silviculture of the University of Gottingen, Technical Cooperation of Federal Republic of Germany, Berlin, p. 296. 1989.

### List of Tables

Table 1 Synoptic table of species

| Species Name C-1 C-2 C-3 C-4 | | | | | |
| --- | --- | --- | --- | --- | --- |
| *Albizia.gummifera* | **6** |  | 1.17 2.35 | | 1 |
| *Ekebergia.capensis* | **5.12** |  | 1.17 0.88 | | 0.42 |
| *Maesa.lanceolata* | 1.35 | | 0 0 | | 0.75 |
| *Carissa.spinarum* | 1.47 | | 0.33 0.71 | | 0 |
| *Rosa.abyssinica* | 1.12 | | 0 0.94 | | 0 |
| *Vernonia amygdalina* | 1.13 | | 0 0 | | 0 |
| *Vernonia.myriantha* | 1.12 | | 1 0.18 | | 0.17 |
| *Clutia.abyssinica* | 1.06 | | 0.5 0.47 | | 0 |
| *Erythrina.brucei* | 0.35 | | 0 0 | | 0 |
| *Maytenus addat* | 0.88 | | 0.33 0 | | 0 |
| *Bridelia.micrantha* | 0.41 | | 0.33 0.12 | | 0 |
| *Hypericum.quartinianum* | 0.35 | | 0 0 | | 0 |
| *Rhamnus.prinoides* | 0.29 | | 0 0.06 | | 0 |
| *Zehneria.scabra* | 0.18 | | 0.17 0.06 | | 0 |
| *Olea.europaea* | 0.71 | | 0 0.12 | | 0 |
| *Periploca linearifolia* | 0.29 | | 0.17 0.12 | | 0 |
| *Embelia.schimperi* | 0.18 | | 0 0.12 | | 0 |
| *Pittosporum.viridiflorum* | 0.76 | | 0 0.12 | | 0 |
| *Brucea.antidysenterica* | 0.47 | | 0.33 0 | | 0.33 |
| *Gnidia.glauca* | 0.18 | | 0 0.12 | | 0 |
| *Croton.macrostachyus* | 4.67 |  | **6.33** | 0.65 0 | |
| *Prunus.Africana* | 0.71 |  | **6.83** | 0.82 3.42 | |
| *Olinia.rochetiana* | 2 |  | 3.67 | 2.47 0 | |
| *Calpurnia.aurea* | 1 |  | 2.33 | 0.88 0 | |
| *Apodytes.dimidiata* | 0.76 |  | 2.33 | 1.18 1.67 | |
| *Osyris.quadripartita* | 0.53 |  | 1 | 0.47 0 | |
| *Bersama.abyssinica* | 0.82 |  | 0.83 | 0 0.25 | |
| *Galiniera.saxifraga* | 0.47 |  | 0.67 | 0 0.17 | |
| *Phytolacca.dodecandra* | 0.18 |  | 0.33 | 0 0 | |
| *Jasminum.abyssinicum* | 0.29 |  | 0.5 | 0.12 0.08 | |
| *Hagenia.abyssinica* | 0 |  | 0.33 | 0 0 | |

| *Clematis longicauda* | 0.29 | 0.33 |  | 0.18 | 0.25 |
| --- | --- | --- | --- | --- | --- |
| *Urera.hypselodendron* | 0.41 | 0.83 |  | 0.41 | 0.58 |
| *Asparagus.africanus* | 0 | 0.33 |  | 0.06 | 0 |
| *Rytigynia.neglecta* | 0.24 | 1 |  | 0.24 | 0.67 |
| *Buddleja.davidii* | 0.06 | 0.83 |  | 0.71 | 0.42 |
| *Vachellia abyssinica* | 0 | 0 |  | **3.41** | 0 |
| *Dombeyatorrida* | 1.06 | 0.67 |  | **3.06** | 0.75 |
| *Allophylus abyssinicus* | 0.65 | 1.67 |  | 2.35 | 0 |
| *Clausena.anisata* | 1.24 | 0.83 |  | 1.29 | 0 |
| *Buddleja.polystachya* | 1 | 0 |  | 1.24 | 0.42 |
| *Dovyalis.abyssinica* | 0.47 | 0.5 |  | 1.24 | 0 |
| *Myrica.salicifolia* | 0.29 | 1 |  | 1.47 | 0 |
| *Maytenus obscura* | 0 | 0 | 1.82 | | 0 |
| *Rhus.vulgaris* | 0.71 | 0.17 | 0.82 | | 0 |
| *Otostegia.integrifolia* | 0.24 | 0 | 0.35 | | 0 |
| *Acanthus.sennii* | 0.53 | 0 | 0.59 | | 0 |
| *Capparis.tomentosa* | 0.12 | 0 | 0.29 | | 0 |
| *Juniperus.procera* | 0 | 0 | 0.35 | | 0 |
| *Rubus.steudneri* | 0.35 | 0 | 0.53 | | 0.17 |
| *Rumex.nervosus* | 0 | 0 | 0.12 | | 0 |
| *Grewia.ferruginea* | 0.12 | 0 | 0.82 | | 0 |
| *Dodonaea.angustifolia* | 0.12 | 0 | 0.29 | | 0 |
| *Maytenus.obscura* | 0 | 0 | 1.82 | | 0 |
| *Myrsine.africana* | 0 | 0 | 0.12 | | 0 |
| *Schefflera.abyssinica* | 0 | 0 | 0 | | **6** |
| *Teclea nobilis* | 0.88 | 0.33 | 1.06 | | **4** |
| *Discopodium.penninervium* | 0.82 | 0 | 0.29 | | 2.92 |
| *Solanecio.gigas* | 0.35 | 1 | 0.12 | | 1.5 |
| *Euphorbia.abyssinica* | 0.29 | 0 | 0 | | 1.33 |
| *Ocimum lamiifolium* | 0 | 0 | 0.29 | | 0.83 |
| *Laggera.tomentosa* | 0 | 0 | 0 | | 0.25 |
| *Rhus glutinosa* | 0 | 0 | 0 | | 0.5 |
| *Dracaena.steudneri* | 0 | 0 | 0 | | 0.17 |

Table 2. Species Richness, Diversity and Evenness of the communities

| Community  type | Richness | Diversity H | Shannon evenness |
| --- | --- | --- | --- |
| 1 | 52 | 3.39 | 0.863 |
| 2 | 34 | 2.99 | 0.847 |
| 3 | 48 | 3.35 | 0.865 |
| 4 | 27 | 2.64 | 0.801 |

Table 3. Sorensen's similarity of the communities

### Community types

| - | C-1 | C-2 | C-3 | C-4 |  |
| --- | --- | --- | --- | --- | --- |
| C-1  C-2 | - 0.43 | - |  |  |  |
| C-3 | 0.45 | 0.41 | - |  |  |
| C-4 | 0.38 | 0.34 | 0.31 | - |  |

*Note: C-1: Albizia gummifera-Ekebergia.capensis, C-2: Prunus Africana- Croton macrostachyus, C-3: Vachellia abyssinica -Dombeya torrida and C-4: Schefflera abyssinica -vepris nobilis community types.*

Table 4	Detrended correspondence analysis results of Seqela forest

|  | **CC1** | **CC2** | **CC3** | **CC4** |
| --- | --- | --- | --- | --- |
| Eigenvalues | 0.5231 | 0.3083 | 0.2267 | 0.2275 |
| Decorana values | 0.5451 | 0.3056 | 0.2090 | 0.1766 |
| Axis lengths | 3.6875 | 2.5179 | 2.9114 | 1.9855 |

Table 5 Results of function donis test of environmental variables (significant environmental variables are indicated by Asterix at their p-value)

Environmental	Df	Sums of Sqs. Mean Sqs	F. Model	R^2^	Pr (>F) variables

| Slope (%) | 1 | 0.7574 | 0.75742 | 3.4429 | 0.04864 | 0.01 ** |
| --- | --- | --- | --- | --- | --- | --- |
| Aspect | 1 | 2.8341 | 2.83412 | 12.8827 | 0.18199 | 0.01 ** |
| Altitude (m) | 1 | 0.5114 | 0.51141 | 2.3246 | 0.03284 | 0.03* |
| MC | 1 | 0.2986 | 0.29864 | 1.3575 | 0.01918 | 0.19 |
| Sand | 1 | 0.3544 | 0.35440 | 1.6109 | 0.02276 | 0.09. |
| Silt | 1 | 0.1304 | 0.13041 | 0.5928 | 0.00837 | 0.74 |
| Clay | 1 | 0.2647 | 0.26471 | 1.2033 | 0.01700 | 0.18 |
| PH | 1 | 0.2393 | 0.23929 | 1.0877 | 0.01537 | 0.33 |
| EC | 1 | 0.1350 | 0.13501 | 0.6137 | 0.00867 | 0.75 |
| OM | 1 | 0.5804 | 0.58039 | 2.6382 | 0.03727 | 0.02 * |
| CEC | 1 | 0.1748 | 0.17482 | 0.7947 | 0.01123 | 0.67 |
| N | 1 | 0.3728 | 0.37280 | 1.6946 | 0.02394 | 0.10 |
| P | 1 | 0.5595 | 0.55946 | 2.5431 | 0.03593 | 0.02 * |
| Residuals | 38 | 8.3598 | 0.21999 | 0.53683 |  |  |
| Total | 52 | 15.5727 |  |  |  | 1.000 |

Signif.codes:	0 ‘***’ 0.001 ‘**’ 0.01 ‘*’ 0.05 ‘.’ 0.1 ‘ ’

Table 6 Bi-plot scores for constrained variables

### Variables CCA1 CCA2 CCA3 CCA4

| Slope | -0.22 | -0.785 | -0.251 | 0.44660 |
| --- | --- | --- | --- | --- |
| Aspect | 0.92 | 0.081 | 0.241 | -0.27716 |
| Altitude | 0.26 | -0.807 | 0.480 | 0.16719 |
| OM | 0.38 | -0.288 | 0.073 | 0.00035 |
| P | 0.60 | 0.334 | 0.162 | 0.62852 |
| Eigenvalue | 0 .3864 | 0.2149 | 0.1497 | 0.1043 |

Proportion Explained 0.4121	0.2292	0.1597	0.1112

Cumulative Proportion 0.4121	0.6413	0.8010	0.9122

Table 7	Distribution of woody plant species in different DBH classes Classes:1 = 2.5-10 cm, 2

= 10.1-20 cm, 3 = 20.1-30 cm, 4 = 30.1-40 cm, 5 = 40.1 - 50cm	and 6 = > 50 cm in Seqela forest

| No | DBH class | No of individuals | density/ha |
| --- | --- | --- | --- |
| 1 | (2.5-10 cm) | 276 | 132.7 |
| 2 | (10.01-20 cm) | 235 | 113 |
| 3 | (20 -30 cm) | 177 | 85.1 |
| 4 | (30.1-40) | 119 | 57.2 |
| 5 | ( > 40.1) | 72 | 34.6 |

Table 8 Basal areas of the top 6 dominant species in Seqela forest

| No | Scientific Name | Basal area (m^2^/ha) |
| --- | --- | --- |
| 1 | *Erythrina brucei* | 6.27 |
| 2 | *Prunus Africana* | 3.67 |
| 3 | *Vachellia abyssinica* | 3.55 |
| 4 | *Apodytes dimidiata* | 2.59 |
| 5 | *Albizia gummifera* | 2.05 |
| 6 | *Schefflera abyssinica* | 2.04 |

Table 9: The highest and lowest (IVI) value of woody plant species in the study area

| NO. | Plant species Name | R.F | R.D | R.DO | IVI | % IVI |
| --- | --- | --- | --- | --- | --- | --- |
| 1 | *Vachellia abyssinica* | 1.55 | 0.535 | 12.954 | 15.039 | 7.07* |
| 2 | *Albizia gummifera* | 5.232 | 6.503 | 7.49 | 19.225 | 9.03* |
| 3 | *Croton macrostachyus* | 4.457 | 8.196 | 3.047 | 15.7 | 7.38* |
| 4 | *Dracaena steudneri* | 0.388 | 0.222 | 0.42 | 1.03 | 0.48** |
| 5 | *Erythrina brucei* | 0.969 | 0.089 | 22.86 | 23.918 | 11.24* |
| 6 | *Hagenia abyssinica* | 0.193 | 0.089 | 0.0274 | 0.3094 | 0.15** |
| 7 | *Juniperus procera* | 0.193 | 0.044 | 1.104 | 1.341 | 0.63** |
| 8 | *Prunus africana* | 3.489 | 1.604 | 13.392 | 18.485 | 8.68* |
| 9 | *Vernonia amygdalina* | 0.775 | 0.357 | 0.128 | 1.26 | 0.59** |

* Indicates the highest value of IVI of woody plant species.

** Indicates the lowest value of IVI of woody plant species

### List of Figures

**Figure 1.** Seqela forest located in Quarit district of West Gojjam Zone, Amhara Region in Ethiopia (Our study does not need to supply a copy right notice for Fig 1. Because the location map of the study area (Fig 1) shape file data was obtained from Ethiopian Mapping Agency (https://africaopendata.org/dataset/ethiopia-shapefiles) is free and open to researchers.

Figure 2. Climate diagram of Quarit (data Source: NMSA, 2022)

Figure 3. Dendrogram of the vegetation data obtained from agglomerative hierarchical cluster analysis of the study area (Community type 1, 2, 3, and 4).

Figure 4. CCA ordination graph, significant environmental variables (p< 0.05), and plant community in the study area

Figure5. Regeneration status of Seqela forest

Cover Letter

[Click here to access/download](https://www2.cloud.editorialmanager.com/pone/download.aspx?id=35998636&guid=bca7dd33-35b4-4658-b50b-04f09f5d5e48&scheme=1)

[Supporting Information Appendix 1.docx](https://www2.cloud.editorialmanager.com/pone/download.aspx?id=35998636&guid=bca7dd33-35b4-4658-b50b-04f09f5d5e48&scheme=1)

Cover Letter

### Woody species diversity, structure and community distribution along environmental gradients of Seqela Dry Afromontane Forest in Northwestern Ethiopia

**Liyew Birhanu^1^*, Getaneh Moges^1^, Nigussie Amsalu^1^**

^1^Department of Biology, Debre Markos University, P.O. Box: 269, Debre Markos, Ethiopia

*****Corresponding

Author: [liyewmtu@gmail.com](mailto:liyewmtu@gmail.com); [**liyew_birhanu@dmu.edu.et**](mailto:liyew_birhanu@dmu.edu.et)

## ABSTRACT

Dry evergreen Afromontane forests are severely threatened due to the expansion of agriculture, overgrazing by livestock, and ineffective management. The objective of this study was to investigate the floristic composition, vegetation structure, and classification of plant communities and assess the relationship between plant community types and environmental variables in the study forest. Systematic sampling was used to collect vegetation and environmental data from 52 (20 m x 20 m) (400 m^2^) plots established at every 200 m intervals along transect lines that were

100 m apart from each other following altitudinal gradient. Density, DBH, basal area, frequency, and importance value indices (IVI) of woody species were computed to characterize the vegetation structure of the forest. Agglomerative hierarchical cluster analysis and canonical Correspondence Analysis (CCA) with R software were used to identify plant communities and analyse the relationship between plant community types and environmental variables, respectively. A total of 68 woody plant species belonging to 63 genera and 44 families were identified. Four plant community types were identified: *Albizia gummifera - Ekebergia capensis, Prunus africana - Croton macrostachyus*, *Vachellia abyssinica - Dombeya torrida* and *Schefflera abyssinica - Teclea nobilis*. The CCA results showed that the variation of species distribution and plant community formation were significantly (P < 0.005) related to altitude, organic matter, aspect, slope, and phosphorus. The total basal area and density of woody species were 27.4 m^2^ ha^−1^ and 1079.3 individual ha^−1^, respectively. The regeneration status assessment of

the forest revealed a poor regeneration status, which was linked to the presence of multiple disturbance factors such as cutting and charcoal production signs. Therefore, it needs immediate conservation to ensure sustainable use and management of the forest.

**Key words:** Environmental gradients, plant community, Seqela forest, species diversity, vegetation structure,

# 1. Introduction

Forest ecosystems are important repositories of terrestrial biodiversity and play a key role in influencing socio-ecological and cultural attributes of human societies including livelihood

activities of traditional societies living as well as associated to these forests [1–3]. These

ecosystems are highly threatened by anthropogenic activities [4 – 6]. Poverty, population pressure, agricultural expansion/intensification and infrastructural development are the major threats to

biodiversity and environment [7–9]. Altitude and latitude have gradients associated to climatic parameters [10-12] which influence the species distribution and their function [13,14]. The fragmentation of a population at an environmental gradient due to disturbance (agents that change the population structure and dynamics), be it anthropogenic or natural, may result in different population growth rates and vital rates among fragments [15, 16]. However, in multispecific or monospecific communities there are cases in which the differentiation of demographic parameters through time is not apparent due to autopoiesis [17, 18], resilience or autogenic regulation [19].

Ethiopia's forests are essential natural resources that offer significant socioeconomic and cultural benefits to local communities [20]. Moreover, these forests play a crucial role in providing various ecosystem services, such as biodiversity conservation, carbon sequestration, and watershed management [21]. However, despite their vital importance, the forest cover in Ethiopia has faced significant threats due to rapid population growth and land use change [22]. According to a recent study [23], Ethiopia's forest cover has decreased by 16% between 1990 and 2020, despite the

revised definition of forests that includes woodlands. Deforestation continues to be a major concern in the country. The diverse environmental conditions in Ethiopia, including variations in altitude, climate, and soil, as well as human interactions, result in varying plant community distribution, structure, and composition [24].

The dry evergreen Afromontane forests in Ethiopia are facing significant threats due to agricultural expansion, overgrazing by livestock, and population growth [22, 20]. Understanding the distribution patterns of plant species and the factors influencing them is essential for effective

biodiversity conservation [25, 26]. Several studies have examined the floristic composition,

structure, and distribution patterns of plant communities along environmental gradients within the

dry evergreen Afromontane forests [e.g. 27–31]. However, no scientific study was made to document woody species composition, structure, and plant community distribution along environmental gradients in the Seqela Dry Afromontane forest. Thus, this study was designed to address the following objectives.(1) to document the woody species composition of the Seqela forest; (2) to identify plant community types in the study area, (3) to investigate the major environmental factors determining species distribution and community composition in the Seqela forest; (4) to examine the vegetation structure and regeneration status of Seqela forest. As a result, this study was conducted to fill the existing gap and provide essential data for the effective conservation and sustainable management of the studied forest [32].

## 2. MATERIALS AND METHODS

### Description of the Study Area

The study was carried out in the Seqela Forest, which is located in Northwestern Ethiopia's Quarit District of the West Gojjam Zone of the Amhara Regional State (Fig. 1). The district is 418 kilometres and 233 kilometres northwest of Addis Ababa and Bahir Dar, respectively, in the northwest region of Ethiopia. Seqela Forest is categorised as dry evergreen Afromontane

Vegetation [33]. The forest is located between 100°56'0'' and 100°57'30'' N and between 370°28'0'' and 370°28'30'' E. Eutricvertsols, a subset of vertisols, are the most common form of soil in the research area[34].

The 10-year climate data (2012-2021) for the Quarit station was obtained from the National Meteorological Services Agency. The average annual rainfall in the district is 1393 mm. The average mean minimum and maximum temperatures in the study area are 100°C and 30.70°C, respectively, with an average annual temperature of about 20.10°C (Figure 2). The main crop types in the district include cereal crops, oil crops, pulses, and vegetables. Cereal crops include

barley (*Hordeum vulgare*), wheat (*Triticum sp.),* teff (*Eragrostis tef*), and maize (*Zea mays*). Oil crops include Niger seed (*Guizotia abyssinica*) and sunflower (*Carthamus tinctorius*). Pulses include faba bean (*Vicia faba*) and field pea (*Pisum sativum*). Vegetables include cabbage (*Brassica oleracea*), potatoes (*Solanum tuberosum*), garlic (*Allium sativum*), and onion (*Allium cepa*). Potatoes are the primary traded tuber crop [35]. Letters of consent were taken from Debre Markos University and Quarit District Agriculture and Rural Development Offices before data collection. Thus, we obtained permission to collect both vegetation and environmental data and conduct our research in the study area.

**Sampling design**

A reconnaissance study was conducted across the forest to determine the sampling plots for the collection of environmental and vegetation data, as well as to provide an overview of the site characteristics. To collect data on vegetation and the environment, a systematic sampling method was applied. A total of 52 plots, 20 m x 20 m along transects following altitudinal gradient. The elevation between two consecutive plots and transect were 100 and 200 ma.s.l apart in the forests, respectively. Five smaller subplots of 5 x 5 m^2^, four at the corner, and one at the centre of the main plot, were established for seedling and sapling species collection [36].

### Vegetation data collection

Trees and shrubs were counted individually in each plot to record the species of woody plants. For shrubs and trees larger than 2.5 cm in diameter, the diameter at breast height (DBH) was measured and recorded. Mature shrubs and trees were defined as reaching more than 2.5 metres tall, whereas saplings between 1 and 2.5 metres and seedlings up to 1 metre were deemed mature. All collected plant specimens were pressed, dried and taken to the Herbarium of Debre Markos University, for taxonomic identification using the published book Flora of Ethiopia and Eritrea. Finally, the presence-absence and cover abundance data were converted to the modified 1–9 Braun Blanquet scale [37].

### Environmental data collection

For every plot within the forest, GPS was used to capture physiographic characteristics like longitude, latitude, and altitude. Each sample plot's slope was determined using a Suunto clinometer. Five locations, each sized 15 × 15 cm and with a depth of 0–30 cm, were used to gather soil samples from each main plot (four from each corner and one from the center) [38]. After combining these samples to create a composite sample, about 1 kg was removed for examination. Before chemical tests, the soil samples were air dried, crushed, and sieved with a 2 mm screen size to remove organic waste and root particles.

Following standard protocols, measurements and analyses were carried out for pH, electrical conductivity (EC), cation exchange capacity (CEC), soil organic matter (SOM), total nitrogen (N), available phosphorus (P), and soil texture (sand, silt, and clay). The soil analysis was assessed at Debre Markos Soil Research and Fertility Improvement's soil laboratory [39].

### Data Analysis

**Plant community classification**

Using the estimated value of percept cover for each woody species, Agglomerative hierarchical cluster analysis was performed in this work to classify the vegetation into plant community types using the free statistical program R version 4.12 [40]. Based on the relative magnitude of their synoptic values, one or two main indicator tree or shrub species are chosen for identifying the community. A total of 64 plant species and 52 sample plots were used in the analysis.

### Diversity and similarity indices

Shannon-Wiener diversity index and Shannon’s evenness were computed to describe the species diversity of the study area [41].

 , where by

H' = Shannon diversity index

**s** = number of species,

p= proportion of individuals or abundance of the ith species expressed as a proportion of total cover in the sample; and *ln* = natural logarithm.

Shannon's evenness index (J) was also calculated using:

where H' = Shannon–Wiener Diversity Index; and H'max = lns, where s is the number of species in the sample.

### Species similarity analysis

So as to compare species composition similarity among community types, Sorensen’s similarity coefficient was calculated by the following formula [41].

Where, Ss = Sorensen’s similarity coefficient

a=number of species common to both samples /communities/study areas b =number of species in sample1, c=number of species in sample 2

### Ordination

A multivariate method called ordination is used to show interactions in a small-scale region between species, plots, and environmental variables [42]. Using SAS software, a preliminary analysis of the data was performed by computing Pearson's correlation coefficient to find significant correlations between environmental variables and remove auto-correlated variables from the ordination analysis. The present study used Canonical Correspondence investigation (CCA) to look at the links between environmental variables and vegetation. Thirteen environmental variables (pH, EC, total nitrogen, available phosphorus, CEC, and OM) as well as

52 sample plots, 64 species, and moisture content have been included in the analysis. To determine the effect of environmental factors on the distribution of plant communities, an Adonis test was performed. Cover abundance values for plant species and information on significant environmental variables were shown in the CCA ordination plot.

### Structural data analysis

Density, basal area, frequency, IVI, and DBH class distributions were calculated for each woody plant species used for the description of vegetation structure. Thus, the following formulas were used to calculate the frequency, density, and basal area of woody species.

**=**(4)

(5)

With ( where	(6)

DBH is diameter at breast height

An importance value index (IVI) was computed for all woody species based on their relative density (RD), relative dominance (RDO), and relative frequency (RF). This index is used to determine the overall importance of each species in the forest system [41].

The Importance Value Index (IVI) for each woody species was computed using the following formula

Where, RDO= relative dominance, BA= Basal area, TBA= total basal area

(7)

(8)

𝑅𝑒𝑙𝑎𝑡𝑖𝑣𝑒 𝐹𝑟𝑒𝑞𝑢𝑒𝑦 =

𝑁𝑢𝑚𝑏𝑒𝑟 𝑜𝑓 𝑠𝑎𝑚𝑝𝑙𝑒 𝑝𝑙𝑜𝑡𝑠 𝑐𝑜𝑛𝑡𝑎𝑖𝑛𝑖𝑛𝑔 𝑎 𝑠𝑝𝑒𝑐𝑖𝑒𝑠

𝑆𝑎𝑚𝑝𝑙𝑒 𝑢𝑛𝑖𝑡𝑠 𝑓𝑜𝑟 𝑎𝑙𝑙 𝑠𝑝𝑒𝑐𝑖𝑒𝑠 𝑜𝑓 𝑡ℎ𝑒 𝑠𝑎𝑚𝑝𝑙𝑒

× 100 ( 9)

𝐼𝑉𝐼 = 𝑅𝑒𝑙𝑎𝑡𝑖𝑣𝑒 𝐷𝑒𝑛𝑠𝑖𝑡𝑦 + 𝑅𝑒𝑙𝑎𝑡𝑖𝑣𝑒 𝐷𝑜𝑚𝑖𝑛𝑎𝑛𝑐 + 𝑅𝑒𝑙𝑎𝑡𝑖𝑣𝑒 𝐹𝑟𝑒𝑞𝑢𝑒𝑛𝑐𝑦, (10)

### Regeneration status of the forest

The regeneration status of the forest was analysed by comparing the density ratios between seedlings and mature individuals, seedlings and saplings, and saplings and mature individuals*.* When seedlings are greater than saplings and saplings greater than adults, the status has fair

regeneration, whereas seedlings are greater than or less than saplings and saplings less than adults; the status is poor regeneration, and the species survives only in the sapling stage (saplings may be less than or greater than adults), and a species is present only in adult form it considered as not regenerating [43].

3. **RESULTS**

### Woody species composition

In the study area, 68 species of woody plants were identified, belonging to 44 families and 63 genera. Out of them, 64 species were found inside the plots, and 4 species—*Maytenus arbutifolia, Ficus sur, Lippia adoensis, and Combretum molle* were found outside the plot (Appendix 1). Of the 64 species of woody plants that have been found, woody lianas make up the least represented growth form, accounting for 10.94% (7 in number) of the species. Of these, trees make up 48.44% (31 in number), and shrubs bring up 40.62% (26 in number) (Appendix 1).

**Plant community types**

Based on the agglomerative hierarchical cluster analysis performed in the study area, four plant community types were identified. The cophenetic correlation coefficient reached a value of 0.774, indicating a considerable degree of similarity in the classification (Figure 3). These communities were grouped as follows: *Prunus africana - Croton macrostachyus; Albizia gummifera – Ekebergia capensis; Vachellia abyssinica - Dombeya torrida; Schefflera abyssinica - Teclea nobilis*

***Albizia gummifera- Ekebergia capensis* Community Type I**

This community type was distributed between altitudinal ranges of 2314 –2520 m.a.s.l. The dominant tree species are *Albizia gummifera, Ekebergia capensis, Croton macrostachyus, Erythrina brucei, Pittosporum viridiflorum* and *Olea europaea* subsp. *cuspidata.* The shrub species *such as Vernonia amygdalina, Maesa lanceolata, Maytenus addat, Clutia abyssinica, Rosa abyssinica, Vernonia myriantha and Bridelia micrantha* were highly dominant. The most

dominant woody Lianas included in this community were *Zehneria scabra* and *Periploca linearifolia.* This community type is represented by 52 species, being the richest in a number of species among the four community types.

***Prunus africana- Croton macrostachyus* Community Type II**

The altitudinal range of this community lies between of 2378 – 2441 m.a.s.l. The most dominant tree species were *Prunus africana, Olinia rochetiana, Apodytes dimidiata* and *Croton macrostachyus.* The shrub layer is dominated by *Osyris quadripartita, Calpurnia aurea, Asparagus africanus, Bersama abyssinica* and *Rytigynia neglecta*. Woody liana species such as *Jasminum abyssinicum, Urera hypselodendron, Clematis longicauda* and *Phytolacca dodecandra* were *dominant.* This community is comprised of 34 species.

***Vachellia abyssinica -Dombeya torrid* Community Type III**

This community type was located between the altitudinal ranges of 2377 – 2577 m.a.s.l. The dominant plant species from this community were *Vachellia abyssinica*, *Dombeya torrida, Allophylus abyssinica, Maytenus obscura, Myrica salicifolia* and *Juniperus procera from* the tree layer and *Dovyalis abyssinica, Capparis tomentosa, Rubus steudneri, Rumex nervosus* and *Otostegia integrifolia* from shrub layer. This community is comprised of 48 species.

***Schefflera abyssinica - Teclea nobilis* CommunityType IV**

This community type was located between altitudinal ranges of 2335 – 2432 m.a.s.l. The dominant plant species in the community were *Schefflera abyssinica, Teclea nobilis, Euphorbia abyssinica, Dracaena steudneri, Solanecio gigas, Laggera tomentosa, Rhus glutinosa* and *Discopodium penninervium* from tree and shrub layer. This community is comprised of 27 species.

### Species richness, evenness, and diversity of community types in the Seqela forest

We calculated the Shannon-Wiener diversity index for each of the four types of plant communities. As a result, according to Shannon's diversity, community 1 had the greatest species diversity and richness, followed by community 3, and community 4 had the lowest. Conversely, communities 1 and 2 were followed in order of evenness value, with community 3 having the greatest value. Among all types of communities, Community 4 had the lowest evenness scoring (Table 2).

### Similarity among community types in the Seqela forest

To find the differences in species composition between the four community types, Sorensen's similarity coefficient (Ss) was calculated. Among all communities, the overall similarity coefficient varies from 31 to 45%. Communities 1 and 2 had the next-most similarity (43%) after C1 and C3 (45%). Relatively little similarity (31%) was found between Communities 3 and 4. (Table 3).

### Relationship between plant community types and environment variables

The gradient length of 3.69 is greater than 3, as shown by the DCA used to choose the optimal ordination technique. As a result, CCA was accepted as an adequate ordination for examining the interactions between the environmental variables and the vegetation. The longest DCA axis for the Sekela forest data set in the present study was 3.69, indicating the heterogeneity of the data as presented in (Table 4). To evaluate the connections between community type and environmental variables, a study using the unimodal response model is suggested. The Canonical correspondence analysis (CCA) ordination method is employed in this analysis.

### CCA Ordination

Thirteen environmental factors were taken into consideration in the ordination analysis: aspect, pH, phosphorus, sand, clay, silt, slope, nitrogen, moisture content, altitude, and Cation Exchange Capacity. As Table 5 and Figure 4 indicate, the only factors that significantly (p < 0.05) correlated with species compositions and their distributions were altitude, OM, slope, aspect, and phosphorus. The canonical correlation analysis (CCA) diagram showed that aspect, av P, and OM were the main variables related to the first axis, and altitude and slope were the major factors associated with the second axis (Figure 4, Table 6). The first and second axes' respective eigenvalues were 0.34 and 0.22.

For the first and second axes, there was heterogeneity in patterns of plant species distribution and plant community formation of 41% and 22.9%, respectively. The results showed that the first two axes accounted for 63.9% of the variation in patterns of plant species distribution and plant community formation. Thus, it was sufficient to reflect the relationship between species and environmental factors using the first two axes.

### Vegetation structure

The distribution of woody plant species in various DBH classes was analyzed and classified into five categories: DBH classes 1 = 2.5-10 cm, 2 = 10.1-20 cm, 3 = 20.1-30 cm, 4 = 30.1-40 cm, and 5 = > 40.1 cm. The first DBH class (2.5-10 cm) had a greater density of woody individuals; 176 of them made up 28.8% of the total. 235 individuals, representing 24.5% of the total, were in the second DBH class (10.1–20 cm) density distribution. The density distributions for the remaining four classes were, in order, 177 (18.5%), 119 (12.4%), 72 (7.5%), and 80 (8.3%). From lower to higher DBH classes, the density distribution of woody individuals showed a declining trend.

In the Seqela natural forest, the overall density of woody species per hectare was 1079.3. *Croton macrostachyus* (88.46 individuals per hectare) was the species with the highest density, followed *by Calpurnia aurea* (68.27 individuals per hectare), *Albizia gummifera* (70.19 individuals per hectare), and *Olinia rochetiana* (78.37 individuals per hectare). *Albizia gummifera* (51.92%), *Croton macrostachyus* (44.23%), *Olinia rochetiana*, and *Teclea nobilis* (36.54%) were the most common woody species in the Seqela natural forest. *Erythrina brucei*, *Juniperus procera*, *Hagenia abyssinica*, *Myrsine africana*, *Laggera tomentosa*, and *Rumex nervosus* were the least common woody species, occurring in 1.92% of cases each. The total basal area of woody plant species with a DBH greater than 2.5 cm was 27.4 m2 per hectare. The basal area of individual plant species increased as their DBH class increased, but their number decreased. Species such as *Erythrina brucei* (6.27 m2/ha), *Prunus africana* (3.67 m2/ha), *Vachellia abyssinica* (3.55 m2/ ha), and *Apodytes dimidiate* (2.59 m2/ha) and *Schefflera abyssinica* (2.04 m2/ha) contributed the largest basal area (Table 8).

### Importance Value Index (IVI) of Species

*Erythrina brucei* (IVI = 11.24), *Prunus africana* (8.68), Croton *macrostachyus* (IVI = 7.38), *Albizia gummifera* (IVI = 9.03), and *Vachellia abyssinica* (7.02) were the most dominant and ecologically significant species in the Seqela natural forest. Conversely, species like *Vernonia amygdalina*, *Juniperus procera*, *Hagenia abyssinica*, and *Dracaena steudneri* had lower IVI values (less than 1) (Table 9).

### Regeneration status of woody plants in Seqela forest

The regeneration status of selected woody plant species in the Seqela forest was assessed to predict the forest's future fate. The regeneration status of the Seqela forest was classified as poor regeneration, as seedlings (44.7 individuals/ha) < saplings (68.3 individuals/ha) < mature trees (461.1 individuals/ha), indicating a J-shaped pattern with a small number of seedlings and

saplings compared to mature trees (Figure 5). Woody plant species such as *Schefflera abyssinica*, *Apodytes dimidiata*, *Erythrina brucei*, *Hagenia abyssinica*, *Juniperus procera, Myrica salicifolia*, and *Prunus africana* were not represented by both seedling and sapling stages. *Pittosporum viridiflorum*, *Vachellia abyssinica*, *Albizia gummifera*, *Buddleja polystachya, Clausena anisata*, *Croton macrostachyus*, *Dombeya torrida*, *Dovyalis abyssinica*, *Euphorbia abyssinica*, *Olea europaea* subsp. *cuspidata*, *Olinia rochetiana*, *Osyris quadripartita*, *Rhamnus prinoides, Rytigynia neglecta*, *Teclea nobilis*, and *Vernonia amygdalina* exhibited fair regeneration status. Conversely, woody species such as *Allophylus abyssinicus*, *Buddleja davidii, Discopodium penninervium*, *Dracaena steudneri*, *Ekebergia capensis*, *Grewia ferruginea*, *Maytenus obscura*, and *Rhus vulgaris* showed poor regeneration status.

## 4. DISCUSSION

### Woody species composition

In the Seqela forest, we found 68 woody species. In contrast to studies conducted in the dry Afromontane forest in Ethiopia, such as those conducted in the Zege Peninsula forest [44] with 113 species, Tara Gedam and Abebaye forests [45] with 143 species, and the forest patches in the Dega Damot district [30] with 106 woody species, the study area showed lower species richness in woody species. The study area's species richness was higher than that of other dry Afromontane forests in Ethiopia, like the 64-species Denkoro Forest in Wello [46], the 50-species Zengena Forest [47], the 66-species Kuandisha Forest [48], and the 57-species Amoro Forest [49].

Fabaceae, Euphorbiaceae, and Rosaceae were among the plant families that were the most common in the study area. The order of families in terms of species richness is consistent with previous studies on dry evergreen Afromontane forests, which indicated the dominance of

Asteraceae [29, 50–51]. Similarly, [48] reported that the most frequent family in the Kuandisha dry evergreen Afromontane forest fragment was Fabaceae. According to [52], the two most

prominent plant families in the flora of Ethiopia and Eritrea are the Asteraceae and Fabaceae. In Seqela Forest, the dominance of Asteraceae and Fabaceae may suggest that disturbances have occurred in the forest. Asteraceae plants are typically ruderal and thrive best in open, disturbed soil [31]. According to [31], the ability of Fabaceae to fix atmospheric nitrogen helps them to survive on soils deficient in nutrients, which accounts for their success in taking over disturbed habitats.

### Plant communities

In the Seqela forest, four plant communities were identified based on the findings of the hierarchical cluster analysis. However, species diversity and richness differed among plant communities. The differences in species composition along the communities may be due to the variation in physico-chemical properties of soil, topography, climate, weathering processes, and microbial activities [53–55] and several other biotic and abiotic factors [14]. For instance, communities 1 and 3 exhibited the highest species richness and diversity. Conversely, community 4 had the lowest species richness and diversity compared to the other communities. The reason for the high species diversity and richness in communities 1 and 3 may be due to the highest altitudinal ranges in which these communities are found, ranging from 2378 to 2441 and 2377 to 2577 m.a.s.l., respectively [39]. Additionally, community 4 was the most disturbed community, likely due to agricultural expansion and overgrazing by livestock.

### Relationship between plant community types and environment variables

The topography (aspect, slope, and altitude) and soil variables combined together to shape the distribution of plant communities in the Seqela forest. The main environmental factors that explained variations in plant species distribution and patterns of plant community formation in the research area were aspect, slope, altitude, OM, and av P, according to the CCA results. According to [56], aspect was shown to be the most significant environmental variable in determining vegetation variation. This could be attributed to variations in incoming solar radiation that impact

temperature and moisture levels. Plant growth and the creation of plant communities are strongly influenced by the temperature and moisture of the soil. Slope and altitude were the primary factors influencing vegetation distribution, according to similar studies conducted in other dry Afromontane forests in Ethiopia [57,49]. A similar study by [30] also confirms that organic matter and av-P were the most important constraining variables in plant community formation of the study area.

### Vegetation structure

The woody plant species distribution in Seqela Forest showed an inverted J-shape pattern, suggesting that small to medium-sized DBH trees dominated the forest rather than large-sized trees. This could be because local communities used these species for various purposes.

The total density of woody species in the Seqela forest was 1079.3 individuals per hectare. This was less than the results of studies conducted by [44] in the Zegie Peninsula Forest (3318 individuals per hectare), Berhanu et al. (2016) in the Kundasha Forest (3086 individuals per hectare), and [48] in the Amoro Forest (2860 individuals per hectare). The density was also higher than other forests, such as Wanzaye natural forest (498) [58]. The degree of anthropogenic interference in various forests may account for this variation in density. In the research area, species with the highest density values were *Clutia abyssinica*, *Croton macrostachyu*s, *Albizia gummifera*, *Olinia rochetiana*, and *Calpurnia aurea*. This might be because the species is small, resulting to a greater total count within a given plot size and a fairly excellent ability for regeneration in dry situations under shade. On the other hand, low-density species may be associated with increased grazing and tree and shrub cutting for charcoal and firewood [59]. The most common species in Seqela woodland were *Albizia gummifera*, *Croton macrostachyus*, *Olinia rochetiana*, *Urera hypselodendron*, and *Teclea nobilis*. These species were pioneer species, as reported by [27] *Albizia gummifera* and *C. macrostachyus* were also the most frequent species in Tara Gedam and Abebaye forests, respectively [45]. The total basal area of the Seqela forest was

27.4 m^2^ per hectare, and the basal area of individual plant species increased as their DBH class increased but their number decreased. The study area had relatively less total basal area than some dry Afromontane forests in Ethiopia, such as Tara Gedam Forest and Abebaye forests [45] and Wof washa forest [27], and Dega Damot district forest patches [39]. On the other hand, the basal area was greater than other forests, such as Amoro forest [49] and Kundasha forest [48]. The total basal area of the study area was comparable to that of the Zengena forest [60]. Species such as *Erythrina brucei*, *Prunus africana*, *Acacia abyssinica, Apodytes dimidiate*, *Albizia gummifera*, and *Schefflera abyssinica* had high basal area due to their large size, even though they had low density. The Importance Value Index (IVI) value was an important parameter used to determine the significance of species in a given ecosystem for conservation purposes [61]. Species with lower IVI values required high conservation efforts [60], while those with higher IVI values needed monitoring and management [59].

### Regeneration status

The regeneration status of natural vegetation is considered poor regeneration if seedlings are greater than or less than saplings and saplings are less than adults [43]. In the case of the Seqela forest, the regeneration status was considered as poor regeneration since seedlings (44.7 individuals per hectare) < saplings (68.3 individuals per hectare) < mature trees (461.1 individuals per hectare). The possible reasons for the existence of poor regeneration status in the forest might be grazing intensity and cutting of young plants for various purposes.

## 5. CONCLUSION

The woody species composition of the Seqela forest indicates a good level of woody species richness in the area. Four community types, including *Albizia gummifera - Ekebergia capensis*, *Prunus Africana* and *Croton macrostachyus*, *Vachellia abyssinica* and *Dombeya torrida, - Schefflera abyssinica - Teclea nobilis,* were identified. Canonical correspondence analysis (CCA)

showed that aspect, altitude, slope, OM, and P were the primary factors of plant distribution patterns and plant community types. This suggests that the distribution of plant species and the formation of plant communities are heavily reliant on these environmental conditions. Species with high densities at lower DBH classes were characteristic of the general vegetation structure of Seqela forest; this may be because shrubby species dominated. The assessment of the forest's regeneration status showed a poor degree of regeneration, which was associated with several disturbance causes such as cutting and signs of charcoal production. As a result of these disturbances, some plant species had no seedlings or saplings, while others had both seedlings and saplings. Species like *Schefflera abyssinica*, *Apodytes dimidiata*, *Erythrina brucei, Hagenia abyssinica*, *Juniperus procera*, *Myrica salicifolia*, and *Prunus africana* were among those with no seedlings and saplings.

### Data Availability

All relevant data are within the manuscript.

**Conflicts of interest**

The authors declare that they have no competing interests

### Acknowledgments

Agriculture and Rural Development Office of the Quarit District as well as to the respective chairpersons is also highly acknowledged.

### Supplementary Materials

Appendix 1: List of woody plant species collected from Seqela forest Quarit District

**References**

1. Hermann, T.M.. Indigenous knowledge and management of Araucaria araucana forest in the Chilean Andes: Implication for native forest conservation. Biological Conservation. 2006; 15: 647-662.
2. Karki H, Kiran Bargali, S.S. Bargali, Vibhuti and Y.S. Rawat. Plant diversity, regeneration status and standing biomass under varied degree of disturbances in temperate mixed oak- conifer forest, Kumaun Himalaya. International Journal of Ecology and Environmental Sciences. 2017;43 (4): 331-345.
3. Baboo B, R Sagar, SS Bargali and H Verma. Tree species composition, regeneration and diversity within the protected area of Indian dry tropical forest. Tropical Ecology. 2017;58(3): 409-423.
4. Htun, N. Z., N. Mizoue & S. Yoshida.. Tree species composition and diversity at different levels of disturbance in Popa Mountain Park, Myanmar. Biotropica. 2011; 43: 597–603.
5. Bisht S, SS Bargali, K Bargali, GS Rawat, YS Rawat and A Fartyal . Influence of Anthropogenic Activities on Forest Carbon Stocks-A Case Study from Gori Valley, Western Himalaya. Sustainability.2022, 14:16918 DOI: 10.3390/su142416918
6. Bisht S, GS Rawat, SS Bargali, YS Rawat and A Mehta. 2023. Forest vegetation response to anthropogenic pressures: a case study from Askot Wildlife Sanctuary, Western Himalaya.	Environment,	Development	and	Sustainability.2023; <https://doi.org/10.1007/s10668-023-03130-2>
7. Davidar, P., S. Sahoo, P. C. Mammen, P. Acharya, J. P. Puyravaud, M. Arjunan, J. P. Garrigues & K. Roessingh. 2010. Assessing the extent and causes of forest degradation in India: Where do we stand? Biological Conservation 143: 2937–2944.
8. Awasthi P, K Bargali SS Bargali and MK Jhariya. Structure and Functioning of Coriaria nepalensis Wall dominated Shrublands in degraded hills of Kumaun Himalaya. I. Dry Matter Dynamics. Land Degradation & Development.2022; 33(9): 1474-1494. <https://doi.org/10.1002/ldr.4235>
9. Pandey R, SS Bargali, K Bargali, VC Pandey.Temporal variability in fine root dynamics in relation to tree girth size in sub-tropical Shorea robusta forests. Land Degradation & Development. 2023;34(5): 1522-1537. <https://doi.org/10.1002/ldr.4550>
10. Arriaga L, Espinoza JM, Aguilar C, Martínez E, Gómez L, Loa E. Regiones terrestres prioritarias de México. Comisión Nacional para el Conocimiento y uso de la Biodiversidad.CONBIO. 2000; México.
11. Fartyal A, K Bargali and SS Bargali.The effect of different slope aspects on plant diversity and soil characteristics in a temperate grassland of Kumaun Himalaya. Vegetos. 2023; <https://doi.org/10.1007/s42535-023-00609-1>
12. Khatri K, B Negi, K Bargali and SS Bargali. Trait plasticity: a key attribute in the invasion success of Ageratina adenophora in different forest types of Kumaun Himalaya, India. Environment, Development and Sustainability. 2023a; DOI: 10.1007/s10668-023-03529-x
13. Manral V, K Bargali, SS Bargali MK Jhariya and K Padalia .Relationships between soil and microbial biomass properties and annual flux of nutrients in Central Himalayan forests, India. Land Degradation & Development.2022; 33 (12): 2014-2025. [https://doi.org/10.1002/ldr.4283.](https://doi.org/10.1002/ldr.4283)
14. Pandey R, SS Bargali, K Bargali, H Karki, and RK Chaturvedi . Dynamics of nitrogen mineralization and fine root decomposition in sub-tropical Shorea robusta Gaertner f. forests of Central Himalaya, India. Science of The Total Environment.2024; 921, <https://doi.org/10.1016/j.scitotenv.2024.170896>
15. Nordstrom SW, Dykstra AB, Wagenius S. Fires slow population declines of a long-lived prairie plant through multiple vital rates. Oecologia.2021;196:679•691, [https://doi.org/10.1007/s00442-021-04955-2.](https://doi.org/10.1007/s00442-021-04955-2)
16. Mourya NR, K Bargali, S.S. Bargali .Effect of Coriaria nepalensis Wall. colonization in a mixed conifer forest of Indian Central Himalaya. Journal of Forestry Research. 2019. ;30 (1), 305-317. DOI: 10.1007/s11676-018-0613-x.
17. Romo-González T, Larralde C, Puga-Olguín A, Vargas-Madrazo E.Hystory, landscapes, metaphors and ghosts around the concelt of specificity. Transdisciplinary Journal of Engineering & Science. 2021. 12, DOI: <https://doi.org/10.22545/2021/00158>.
18. Mackey B, Morgan E, Keith H.Evaluating forest landscape management for ecosystem integrity. Landscape Research, DOI: 10.1080/01426397.2023.2284938.
19. Muñoz R, Bongers F, Rozendaal DMA, González EJ, Dupuy JM, Meave JA. Autogenic regulation and resilience in tropical dry forest. Journal of Ecology.2021 109:3295• 3307,
20. Meragiaw, M, Woldu, Z, Martinsen, V. Floristic composition and structure of the Kibate Forest along environmental gradients in Wonchi, Southwestern Ethiopia. *J. For. Res.* 2021;**32**, 2669–2682, <https://doi.org/10.1007/s11676-021-01305-z>
21. Fekadu Gurmessa, Bikila Warkineh, Teshome Soromessa & Sebsebe Demissew). Species diversity and plant community distribution along environmental gradient in Tulu Lafto Forest, western Ethiopia. Phytocoenologia 2022
22. Williams, S.D., J.L. Vivero, S. Spawls, Aneh S. & Ensermu K. Ethiopian Highlands. In: Mittermeier, R.A., P. Robles-Gil, M. Hoffmann, J.D. Pilgrim, T.M. Brooks, C.G. Mittermeier & G. Fonseca (eds.), Hotspots Revisited: Earth´s Biologically Richest and Most Endangered Ecoregions, 2004; pp. 262-273. CEMEX, Mexico city, Mexico.
23. FAO, *Global Forest Resources Assessment: Main report* (Food and Agriculture Organization of the United Nations, 2020; Rome
24. Yemata G, Haregewoien G. Floristic composition, structure and regeneration status of woody plant species in Northwest Ethiopia,Trees, Forests and People. 2022;Volume 9,https://doi.org/10.1016/j.tfp.2022.100291
25. Brooks TM, Mittermeier RA, Mittermeier CG. Habitat loss and extinction in the hotspots of biodiversity. Conserv Biol 16:909–923
26. Magurran AE. Measuring biological diversity. Blackwell Science. 2004 , ltd, Malden, p 132
27. Bekele T. Vegetation ecology of remnant Afromontane forests on the central plateau of Shewa, Ethiopia. Sv. växtgeografiska sällsk. 1993
28. Teketay D, Bekele T.Floristic composition of Wof‐ Washa natural forest, Central Ethiopia: Implications for the conservation of biodiversity. Feddes Repertorium. 1995 ; 106 (1‐ 5):127–47.
29. Yineger H, Kelbessa E, Bekele T, Lulekal E. Floristic composition and structure of the dry Afromontane forest at Bale Mountains National Park, Ethiopia. SINET: Ethiopian Journal of Science. 2008; 31(2):103–20.
30. Birhanu L, BekeleT., Binyam Tesfaw, and Sebsebe Demissew. Relationships between topographic factors soil and plant communities in a dry Afromontane forest patches of North western Ethiopia. PLOS ONE, 2021; 16:1–18.
31. Dagne Y, Birhanu L. Floristic composition and plant community distribution along environmental gradients in Guard dry Afromontane forest of Northwestern Ethiopia. BMC Ecol Evol. 2023; 28;23(1):43. doi: 10.1186/s12862-023-02154-6.
32. Girmay, M., Bekele, T., Demissew, S. *et al.* Ecological and floristic study of Hirmi woodland vegetation in Tigray Region, Northern Ethiopia. *Ecol Process.2020;* **9**, 53 <https://doi.org/10.1186/s13717-020-00257-2>
33. Friis, I., Demissew S and van Breugel, P. Atlas of the potential vegetation of Ethiopia. The royal Danish Academy of Sciences and Letters.2010; Copenhagen,DK

| 34. Tassew | A, | Teffera | B, | Jafar | E, | Ashagrie | E, | Mulugeta | G, |
| --- | --- | --- | --- | --- | --- | --- | --- | --- | --- |
| Tesfaye | G, | Adimasu | S, | Limenih |  | S, Genet | T, | Yihenew | G. |

Selassie andLeyew Z. Comprehensive study on research, development and management interventions in Birr sub-watershed, Quarit district, West Gojjam zone, Amhara national regional state. 2011.

35. Gebey T and Mekuriaw Z. Diagnosis and intervention plans for west Gojjam zone, Amhara Region, Ethiopia. 2013.
36. Masresha G, Asnakew M, Chekole G, Melese G. Comparative study on floristic composition vegetation structure and regeneration status of woody forests in case of GondTeklehaimaot and Arsema Monastery Forest, Department of Biology, College of Natural and Computational Sciences, University of Gondar, Ethiopia.2019.
37. Van der Maarel, E. Transformation of Cover abundance values in phytosociology and its effects on community similarity. *Vegetation.1979;* ***3*9**:97-114
38. Tadesse G, Bekele T, Demissew S. Dryland woody vegetation along an altitudinal gradient on the eastern escarpment of Wolo, Ethiopia. SINET: Ethiop J Sci 2008; 31:43–54
39. Birhanu L. Ecological Study of the Vegetation and Land Use/ Land Cover Change analysis in Dega Damot District, West Gojjam Zone, Amhara Region, NorthwesternEthiopia PhD thesis ; Addis Ababa University, Ethiopia. 2020
40. R Development Core Team. R: A Language and Environment for Statistical Computing. A Programming Environment for Data Analysis and Graphics. Version 4.12. 2022
41. Kent M. Vegetation Description and Data Analysis a Practical Approach 2nd Eds. Blackwell Publishing, UK.
42. McCune B, Grace J, Urban D. Analysis of ecological communities. Gleneden Beach, OR: MjM software design. 2002.
43. Dhaulkhandi, M., Dobhal, A., Bhatt, S. and Kumar, M. Community structure and regeneration potential of natural forest site in Gangotri , India. J Basic Appli Sci.2008.
44. Alelign A , Teketay D, Yemshawy, & Sue Edwards .Diversity and status of regeneration of woody plants on the peninsula of Zegie, northwestern Ethiopia**.***Tropical Ecology.2007.* 48(1)**:** 37-49, 2007 ISSN 0564-3295.
45. Zegeye H, Teketay D and Kelbessa K. Diversity and regeneration status of woody species in Tara Gedam and Abebaye Forests, north-westernEthiopia. Journal of Forestry Research.2011; 22 (3):315–328
46. Ayalew A. A Floristic composition and structural analysis of Denkoro Forest, South Wello.M.Sc. Thesis. Addis Ababa University.2003.
47. Tadele D, Lulekal E, Damtie D and Assefa A. Floristic diversity and regeneration status of woody plants in Zengena forest, a remnant montane forest patch in northwestern Ethiopia. Journal of forestry research.2014; 25 (2):329–336.
48. Berhanu A, Demissew S, Woldu Z and Didita M. Woody species composition andstructure of KuandishaAfromontane forest fragment in northwestern Ethiopia.Journal of Forestry Research.2016; 28: 343-355.
49. Birhanu L, Bekele T and Demissew S . Woody species composition and structure of AmoroForest in West Gojjam Zone, Northwestern Ethiopia. *Journal of ecology andthe natural environment. 2018;***10** (4): 53-64.
50. Tilahun A, Soromessa T, Kelbessa E, and Dibaba A. Floristic composition and community analysis	of	Menagesha	Amba	Mariam	Forest (Egdu Forest) in the central plateau of Shewa, Ethiopia.Ethiopian Journal of Biological Sciences. 2011; **10** (2): 111-136.
51. Lulekal E. Plant diversity and ethnobotanical study of medicinal plants in Ankober District, North ShewaZone of Amhara Region, Ethiopia. Ph.D. Dissertation, Addis Ababa University. 312p. 2014.
52. Tadesse M. Asteraceae (Compositae). In: Hedberg, I., Friis, I., Edwards, S., (eds). Flora of Ethiopia and Eritrea. Vol. 4(1). The National Herbarium, AAU, Addis Ababa & Uppsala.2004.
53. Paudel, S., Sah, J.P. Physico chemical characters of soil in tropical soil (Shorea robusta Gaertn.) forests in eastern Nepal. Himal. J. Sci. 2003; 1 (2), 107–110.
54. Padalia K, SS Bargali, K Bargali and V Manral. Soil microbial biomass phosphorus under different land use systems. Tropical Ecology 63:30-48.2022; <https://doi.org/10.1007/s42965-021-00184-z>
55. Manral V, K Bargali, SS Bargali and C Shahi. Changes in soil biochemical properties following replacement of Banj oak forest with Chir pine in Central Himalaya, India. Ecological Processes.2020; 9:30 <https://doi.org/10.1186/s13717-020-00235-8>
56. Paudel S and O. R. Vetaas. “Effects of topography and land use on woody plant species composition and beta diversity in an arid Trans-Himalayan landscape, Nepal,” *Journal of Mountain Science*, 2014; vol. 11, no. 5, pp. 1112–1122
57. Shiferaw W, Bekele T, Demissew S. Anthropogenic effects on floristic composition, diversity	and	regeneration	potential	of	the	Debre	libanos Monastery forest patch, central Ethiopia. J For Res.2018; ;30(6):2151–61
58. Getnet A. Woody Species Composition, Diversity and Vegetation Structure of Dry Afromontane Forest, Ethiopia.*Journal of Agriculture and Ecology Research International. 2018;* ***16***(3): 1-20.
59. Birhanu L, Bekele T, Tesfaw B, Demissew S. Soil seed bank composition and aboveground vegetation in dry Afromontane forest patches of Northwestern Ethiopia,Trees, Forests and People,2022; Volume 9, <https://doi.org/10.1016/j.tfp.2022.100292>.
60. Tadele D, Lulekal E, Damtie D and Assefa A. Floristic diversity and regeneration status of woody plants in Zengena forest, a remnant montane forest patch in northwestern Ethiopia. Journal of forestry research.2014; 25 (2):329–336.
61. Lamprecht, H. Tropical forest ecosystems and their tree species. Silviculture in Tropics, Possibilities, and Methods for Their Long-Term Utilization. Institute of for silviculture of the University of Gottingen, Technical Cooperation of Federal Republic of Germany, Berlin, p. 296. 1989.

### List of Tables

Table 1 Synoptic table of species

| Species Name C-1 C-2 C-3 C-4 | | | | | |
| --- | --- | --- | --- | --- | --- |
| *Albizia.gummifera* | **6** |  | 1.17 2.35 | | 1 |
| *Ekebergia.capensis* | **5.12** |  | 1.17 0.88 | | 0.42 |
| *Maesa.lanceolata* | 1.35 | | 0 0 | | 0.75 |
| *Carissa.spinarum* | 1.47 | | 0.33 0.71 | | 0 |
| *Rosa.abyssinica* | 1.12 | | 0 0.94 | | 0 |
| *Vernonia amygdalina* | 1.13 | | 0 0 | | 0 |
| *Vernonia.myriantha* | 1.12 | | 1 0.18 | | 0.17 |
| *Clutia.abyssinica* | 1.06 | | 0.5 0.47 | | 0 |
| *Erythrina.brucei* | 0.35 | | 0 0 | | 0 |
| *Maytenus addat* | 0.88 | | 0.33 0 | | 0 |
| *Bridelia.micrantha* | 0.41 | | 0.33 0.12 | | 0 |
| *Hypericum.quartinianum* | 0.35 | | 0 0 | | 0 |
| *Rhamnus.prinoides* | 0.29 | | 0 0.06 | | 0 |
| *Zehneria.scabra* | 0.18 | | 0.17 0.06 | | 0 |
| *Olea.europaea* | 0.71 | | 0 0.12 | | 0 |
| *Periploca linearifolia* | 0.29 | | 0.17 0.12 | | 0 |
| *Embelia.schimperi* | 0.18 | | 0 0.12 | | 0 |
| *Pittosporum.viridiflorum* | 0.76 | | 0 0.12 | | 0 |
| *Brucea.antidysenterica* | 0.47 | | 0.33 0 | | 0.33 |
| *Gnidia.glauca* | 0.18 | | 0 0.12 | | 0 |
| *Croton.macrostachyus* | 4.67 |  | **6.33** | 0.65 0 | |
| *Prunus.Africana* | 0.71 |  | **6.83** | 0.82 3.42 | |
| *Olinia.rochetiana* | 2 |  | 3.67 | 2.47 0 | |
| *Calpurnia.aurea* | 1 |  | 2.33 | 0.88 0 | |
| *Apodytes.dimidiata* | 0.76 |  | 2.33 | 1.18 1.67 | |
| *Osyris.quadripartita* | 0.53 |  | 1 | 0.47 0 | |
| *Bersama.abyssinica* | 0.82 |  | 0.83 | 0 0.25 | |
| *Galiniera.saxifraga* | 0.47 |  | 0.67 | 0 0.17 | |
| *Phytolacca.dodecandra* | 0.18 |  | 0.33 | 0 0 | |
| *Jasminum.abyssinicum* | 0.29 |  | 0.5 | 0.12 0.08 | |
| *Hagenia.abyssinica* | 0 |  | 0.33 | 0 0 | |

| *Clematis longicauda* | 0.29 | 0.33 |  | 0.18 | 0.25 |
| --- | --- | --- | --- | --- | --- |
| *Urera.hypselodendron* | 0.41 | 0.83 |  | 0.41 | 0.58 |
| *Asparagus.africanus* | 0 | 0.33 |  | 0.06 | 0 |
| *Rytigynia.neglecta* | 0.24 | 1 |  | 0.24 | 0.67 |
| *Buddleja.davidii* | 0.06 | 0.83 |  | 0.71 | 0.42 |
| *Vachellia abyssinica* | 0 | 0 |  | **3.41** | 0 |
| *Dombeyatorrida* | 1.06 | 0.67 |  | **3.06** | 0.75 |
| *Allophylus abyssinicus* | 0.65 | 1.67 |  | 2.35 | 0 |
| *Clausena.anisata* | 1.24 | 0.83 |  | 1.29 | 0 |
| *Buddleja.polystachya* | 1 | 0 |  | 1.24 | 0.42 |
| *Dovyalis.abyssinica* | 0.47 | 0.5 |  | 1.24 | 0 |
| *Myrica.salicifolia* | 0.29 | 1 |  | 1.47 | 0 |
| *Maytenus obscura* | 0 | 0 | 1.82 | | 0 |
| *Rhus.vulgaris* | 0.71 | 0.17 | 0.82 | | 0 |
| *Otostegia.integrifolia* | 0.24 | 0 | 0.35 | | 0 |
| *Acanthus.sennii* | 0.53 | 0 | 0.59 | | 0 |
| *Capparis.tomentosa* | 0.12 | 0 | 0.29 | | 0 |
| *Juniperus.procera* | 0 | 0 | 0.35 | | 0 |
| *Rubus.steudneri* | 0.35 | 0 | 0.53 | | 0.17 |
| *Rumex.nervosus* | 0 | 0 | 0.12 | | 0 |
| *Grewia.ferruginea* | 0.12 | 0 | 0.82 | | 0 |
| *Dodonaea.angustifolia* | 0.12 | 0 | 0.29 | | 0 |
| *Maytenus.obscura* | 0 | 0 | 1.82 | | 0 |
| *Myrsine.africana* | 0 | 0 | 0.12 | | 0 |
| *Schefflera.abyssinica* | 0 | 0 | 0 | | **6** |
| *Teclea nobilis* | 0.88 | 0.33 | 1.06 | | **4** |
| *Discopodium.penninervium* | 0.82 | 0 | 0.29 | | 2.92 |
| *Solanecio.gigas* | 0.35 | 1 | 0.12 | | 1.5 |
| *Euphorbia.abyssinica* | 0.29 | 0 | 0 | | 1.33 |
| *Ocimum lamiifolium* | 0 | 0 | 0.29 | | 0.83 |
| *Laggera.tomentosa* | 0 | 0 | 0 | | 0.25 |
| *Rhus glutinosa* | 0 | 0 | 0 | | 0.5 |
| *Dracaena.steudneri* | 0 | 0 | 0 | | 0.17 |

Table 2. Species Richness, Diversity and Evenness of the communities

| Community  type | Richness | Diversity H | Shannon evenness |
| --- | --- | --- | --- |
| 1 | 52 | 3.39 | 0.863 |
| 2 | 34 | 2.99 | 0.847 |
| 3 | 48 | 3.35 | 0.865 |
| 4 | 27 | 2.64 | 0.801 |

Table 3. Sorensen's similarity of the communities

### Community types

| - | C-1 | C-2 | C-3 | C-4 |  |
| --- | --- | --- | --- | --- | --- |
| C-1  C-2 | - 0.43 | - |  |  |  |
| C-3 | 0.45 | 0.41 | - |  |  |
| C-4 | 0.38 | 0.34 | 0.31 | - |  |

*Note: C-1: Albizia gummifer-Ekebergia.capensis, C-2: Prunus Africana- Croton macrostachyus, C-3: Vachellia abyssinica -Dombeya torrida and C-4: Schefflera abyssinica -vepris nobilis community types.*

Table 4	Detrended correspondence analysis results of Seqela forest

|  | **CC1** | **CC2** | **CC3** | **CC4** |
| --- | --- | --- | --- | --- |
| Eigenvalues | 0.5231 | 0.3083 | 0.2267 | 0.2275 |
| Decorana values | 0.5451 | 0.3056 | 0.2090 | 0.1766 |
| Axis lengths | 3.6875 | 2.5179 | 2.9114 | 1.9855 |

Table 5 Results of function donis test of environmental variables (significant environmental variables are indicated by Asterix at their p-value)

Environmental	Df	Sums of Sqs. Mean Sqs	F. Model	R^2^	Pr (>F) variables

| Slope (%) | 1 | 0.7574 | 0.75742 | 3.4429 | 0.04864 | 0.01 ** |
| --- | --- | --- | --- | --- | --- | --- |
| Aspect | 1 | 2.8341 | 2.83412 | 12.8827 | 0.18199 | 0.01 ** |
| Altitude (m) | 1 | 0.5114 | 0.51141 | 2.3246 | 0.03284 | 0.03* |
| MC | 1 | 0.2986 | 0.29864 | 1.3575 | 0.01918 | 0.19 |
| Sand | 1 | 0.3544 | 0.35440 | 1.6109 | 0.02276 | 0.09. |
| Silt | 1 | 0.1304 | 0.13041 | 0.5928 | 0.00837 | 0.74 |
| Clay | 1 | 0.2647 | 0.26471 | 1.2033 | 0.01700 | 0.18 |
| PH | 1 | 0.2393 | 0.23929 | 1.0877 | 0.01537 | 0.33 |
| EC | 1 | 0.1350 | 0.13501 | 0.6137 | 0.00867 | 0.75 |
| OM | 1 | 0.5804 | 0.58039 | 2.6382 | 0.03727 | 0.02 * |
| CEC | 1 | 0.1748 | 0.17482 | 0.7947 | 0.01123 | 0.67 |
| N | 1 | 0.3728 | 0.37280 | 1.6946 | 0.02394 | 0.10 |
| P | 1 | 0.5595 | 0.55946 | 2.5431 | 0.03593 | 0.02 * |
| Residuals | 38 | 8.3598 | 0.21999 | 0.53683 |  |  |
| Total | 52 | 15.5727 |  |  |  | 1.000 |

Signif.codes:	0 ‘***’ 0.001 ‘**’ 0.01 ‘*’ 0.05 ‘.’ 0.1 ‘ ’

Table 6 Bi-plot scores for constrained variables

### Variables CCA1 CCA2 CCA3 CCA4

| Slope | -0.22 | -0.785 | -0.251 | 0.44660 |
| --- | --- | --- | --- | --- |
| Aspect | 0.92 | 0.081 | 0.241 | -0.27716 |
| Altitude | 0.26 | -0.807 | 0.480 | 0.16719 |
| OM | 0.38 | -0.288 | 0.073 | 0.00035 |
| P | 0.60 | 0.334 | 0.162 | 0.62852 |
| Eigenvalue | 0 .3864 | 0.2149 | 0.1497 | 0.1043 |

Proportion Explained 0.4121	0.2292	0.1597	0.1112

Cumulative Proportion 0.4121	0.6413	0.8010	0.9122

Table 7	Distribution of woody plant species in different DBH classes Classes:1 = 2.5-10 cm, 2

= 10.1-20 cm, 3 = 20.1-30 cm, 4 = 30.1-40 cm, 5 = 40.1 - 50cm	and 6 = > 50 cm in Seqela forest

| No | DBH class | No of individuals | density/ha |
| --- | --- | --- | --- |
| 1 | (2.5-10 cm) | 276 | 132.7 |
| 2 | (10.01-20 cm) | 235 | 113 |
| 3 | (20 -30 cm) | 177 | 85.1 |
| 4 | (30.1-40) | 119 | 57.2 |
| 5 | ( > 40.1) | 72 | 34.6 |

Table 8 Basal areas of the top 6 dominant species in Seqela forest

| No | Scientific Name | Basa area (m^2^/ha) |
| --- | --- | --- |
| 1 | *Erythrina brucei* | 6.27 |
| 2 | *Prunus Africana* | 3.67 |
| 3 | *Vachellia abyssinica* | 3.55 |
| 4 | *Apodytes dimidiata* | 2.59 |
| 5 | *Albizia gummifera* | 2.05 |
| 6 | *Schefflera abyssinica* | 2.04 |

Table 9: The highest and lowest (IVI) value of woody plant species in the study area

| NO. | Plant species Name | R.F | R.D | R.DO | IVI | % IVI |
| --- | --- | --- | --- | --- | --- | --- |
| 1 | *Vachellia abyssinica* | 1.55 | 0.535 | 12.954 | 15.039 | 7.07* |
| 2 | *Albizia gummifera* | 5.232 | 6.503 | 7.49 | 19.225 | 9.03* |
| 3 | *Croton macrostachyus* | 4.457 | 8.196 | 3.047 | 15.7 | 7.38* |
| 4 | *Dracaena steudneri* | 0.388 | 0.222 | 0.42 | 1.03 | 0.48** |
| 5 | *Erythrina brucei* | 0.969 | 0.089 | 22.86 | 23.918 | 11.24* |
| 6 | *Hagenia abyssinica* | 0.193 | 0.089 | 0.0274 | 0.3094 | 0.15** |
| 7 | *Juniperus procera* | 0.193 | 0.044 | 1.104 | 1.341 | 0.63** |
| 8 | *Prunus africana* | 3.489 | 1.604 | 13.392 | 18.485 | 8.68* |
| 9 | *Vernonia amygdalina* | 0.775 | 0.357 | 0.128 | 1.26 | 0.59** |

* Indicates the highest value of IVI of woody plant species.

** Indicates the lowest value of IVI of woody plant species

### List of Figures

**Figure 1.** Seqela forest located in Quarit district of West Gojjam Zone, Amhara Region in Ethiopia (Our study does not need to supply a copy right notice for Fig 1. Because the location map of the study area (Fig 1) shape file data was obtained from Ethiopian Mapping Agency (https://africaopendata.org/dataset/ethiopia-shapefiles) is free and open to researchers.

Figure 2. Climate diagram of Quarit (data Source: NMSA, 2022)

Figure 3. Dendrogram of the vegetation data obtained from agglomerative hierarchical cluster analysis of the study area (Community type 1, 2, 3, and 4).

Figure 4. CCA ordination graph, significant environmental variables (p< 0.05), and plant community in the study area

Figure5. Regeneration status of Seqela forest

Cover Letter

### Manuscript Number: PONE-D-24-05897R2

**Woody species diversity, Vegetation structure and Plant community distribution along environmental gradients of Seqela Dry Afromontane Forest in Northwestern Ethiopia**

PLOS ONE

**Article type:** Research Paper

We would like to thank the reviewers for their efforts in reviewing the manuscript and providing valuable suggestions for improving the article. All comments and suggestions were

addressed. The changes are marked in yellow color as shown in the revised manuscript. We detailed response for each comment is provided below.

### Editor

The authors have appropriately revised the manuscript and answered all the questions of the reviewers satisfactorily. I recommend the revised version of the manuscript be accepted for publication in PLOS ONE after minor revision.

**Author’s Response:** Thanks a lot for letting us revise our paper. Below are the responses to a reviewer one comments.

### Reviewer #1:

1. **General comments**

The authors have satisfactorily addressed the majority of my concerns and comments, although some minor ones still need to be addressed.

**Author’s Response:** Thanks a lot for this very positive feedback.

2. **Title**: It needs minor revision “Woody species diversity, structure, and community distribution along environmental gradients of Seqela Dry Afromontane Forest in Northwestern Ethiopia”

**Author’s Response:** Thank you for this.

3. In paragraph 2, replace “[25; 26]” with “[25, 26].

“… environmental factors [e.g. 27- 31]” please restate this statement. Additionally, use proper punctuation and writing techniques throughout your paper such as, replace “27- 31” with “27–31”.

**Author’s Response:** The correction has been made in the revised manuscript.

### 4. Study area

Paragraph 2 replaces “*Eragrostis teff*” with “*Eragrostis tef*”.

**Author’s Response:** Thank you very much for your excellent observation. We have corrected in the revised version of the manuscript.

### Data Analysis

**Plant community classification**

##There is a mix of information herein: “agglomerative hierarchical cluster analysis”? Select either “agglomerative cluster analysis” or “hierarchical cluster analysis” with acceptable reason.

##The author did not give sufficient response for this question raised in the first draft.

**Author’s Response:** Based on your previous comment, we have corrected it as “agglomerative hierarchical cluster analysis”. Let's clarify this comment.

Cluster analysis in ecology is a statistical method used to classify objects (such as species, communities, or individuals) into groups or clusters based on similarity. There are several types of cluster analysis techniques commonly used in ecology. Basically the methods of cluster analysis fall into **hierarchical and non-hierarchical (partitioning) techniques**.

### 1. Hierarchical clustering

- Hierarchical clustering is an assembling technique of vegetation stands that could be classified into groups, the processes being repeated at different levels to form a tree.
- There are two types of hierarchical clustering methods, namely **agglomerative and divisive**.

A. **Agglomerative hierarchical clustering:** It starts with each object as a separate cluster and then iteratively merges the closest clusters until only one cluster remains. called “**bottom up method**”, construct a tree-like hierarchy,
B. **Divisive Hierarchical Clustering :** Divisive hierarchical clustering method is the opposite of agglomerative classification in that the clustering start at the top and work downwards, beginning with the whole collection of plots, and dividing them to form successive subclasses and ultimately form a more or less similar groups. This method is not too common method for plant classification.

- Agglomerative Hierarchical Clustering is often preferred for plant community classification because it starts with individual data points and progressively groups them

together based on similarity, mirroring the natural progression of plant community formation where individual plants come together to form larger groupings. This approach aligns well with the hierarchical structure often observed in plant communities, making it a suitable method for classification.

2. **Partitioning (non-hierarchical clustering):** Partitioning is a method of classifying vegetation stands in the same level, not in hierarchy ( eg. K- means clustering, Fuzzy clustering).

### Results

**Plant community types**

\## “Figure 3. Dendrogram”, here is my big question why not 2 types or 3 community types or else? Because the author did not show the value of the cophenetic correlation coefficient where they determined the cutree. The author did not give sufficient response for this question raised in the first round of review rather they replied very subjective suggestions.

**Author’s Response:** Again, Let's clarify this comment. The cophenetic correlation coefficient can be used as some kind of measure of the goodness of fit of a particular dendrogram. Based on the agglomerative hierarchical cluster analysis performed in the study area, four plant community types were identified. The cophenetic correlation coefficient reached a value of 0.774, indicating a considerable degree of similarity in the classification. This information included in the revised manuscript.

Figure 3. Dendrogram”, here is my big question why not 2 types or 3 community types or else?

**Author’s Response:** we appreciate you raising this question. I (corresponding author) frequently inquire about this question when examining the students or reviewing manuscripts. Determining the number of groups in a cluster analysis is often the primary goal. Among the methods we used to decide the optimum cluster is **Elbo method**, finally we have got 4 plant community types.
