# Supplementary material for: Woody species diversity, structure and community distribution along environmental gradients of Seqela Dry Afromontane forest in Northwestern Ethiopia
Source: PLoS One. 2025 Jan 17;20(1):e0313020. doi: 10.1371/journal.pone.0313020 (PMC11741620; doi:10.1371/journal.pone.0313020)
Supplement: Attachment — Submitted filename: Point by point.docx [file pone.0313020.s009.docx]

**Manuscript Number: PONE-D-24-05897R3**

**Woody species diversity, Vegetation structure and Plant community distribution along environmental gradients of Seqela Dry Afromontane Forest in Northwestern Ethiopia**

PLOS ONE

**Article type:** Research Paper

We would like to thank the reviewers for their efforts in reviewing the manuscript and providing valuable suggestions for improving the article. All comments and suggestions were addressed. The changes are marked in yellow color as shown in the revised manuscript. We detailed response for each comment is provided below.

**Reviewer #1**:

**Author’s Response:** Thanks a lot for this very positive feedback.

The correction has been made in the revised manuscript.

**Reviewer #3:** The study focuses on an interesting and timely area of research. However, the write up, organization, grammar and especially the methodology are not to the standard. The methodology missed how the environmental factors such as altitude, aspect and slope were considered during plot establishment, while most of the data and the conclusion relied on. There are some data such as Shannon diversity index and evenness already collected, but not presented.

**Author’s Response:** We have collected data on altitude, aspect, and slope, and included these variables in the ordination analysis. The conclusions are based on this analysis, with Table 9 providing supporting evidence. But we have included miss information provided in the methodology section. We have also conducted an overall diversity analysis, which can be reviewed in Appendix 2.

**Abstract**

1. The abstract lacks the major results of the study such as

Diversity and evenness data (diversity index0

Vegetation structure (height, DBH, frequency, IVI etc

The order of presenting the results is not logical. It should start with diversity, structure, regeneration and community types

**Author’s Response:** The correction has been made in the revised manuscript.

**Introduction**

Since you are going to study the influence of environmental factors (altitude, aspect, slope and edaphic factors), the introduction should clearly show this through review of literature.

This has to be addressed in detail so that the readers can see the research gap.

**Author’s Response:** Thank you very much for your very good observation. We have corrected in the revised version of the manuscript.

**Method**

Altitude, slope and aspect are the environmental factors that you studied. However, you didn’t mention the slope and aspect you gathered vegetation and soil data?

**Author’s Response:** The correction has been made in the revised manuscript.

/// Letters of consent were taken from Debre Markos University and Quarit District Agriculture and Rural Development Offices before data collection. Thus, we obtained permission to collect both vegetation and environmental data and conduct our research in the study area. I don’t think that this is important for your study as long as you didn’t involve and gather data from human beings

**Author’s Response:** Permission for field work is one of the criteria for PLOSE ONE guideline.
