# Supplementary material for: Woody species diversity, structure and community distribution along environmental gradients of Seqela Dry Afromontane forest in Northwestern Ethiopia
Source: PLoS One. 2025 Jan 17;20(1):e0313020. doi: 10.1371/journal.pone.0313020 (PMC11741620; doi:10.1371/journal.pone.0313020)
Supplement: Attachment — Submitted filename: Point by point_R4.docx [file pone.0313020.s010.docx]

**Manuscript Number: PONE-D-24-05897R4**

**Woody Species Diversity, Structure and Plant Community Distribution Along Environmental Gradients of Seqela Dry Afromontane Forest in Northwestern Ethiopia**

PLOS ONE

**Article type:** Research Paper

We would like to thank the reviewers for their efforts in reviewing the manuscript and providing valuable suggestions for improving the article. All comments and suggestions were addressed. The changes are marked in yellow color as shown in the revised manuscript. We detailed response for each comment is provided below.

**Reviewer #3: -** The authors have addressed the comments given in the first round revision and the manuscript is very much improved. The comments for further improvement include
1. The size of the study forest is not mentioned. However, in the discussion under woody species composition, a sentence saying the variation in Shannon diversity index might be due to the size of the forest is included. Therefore, including the size the forest in the paper helps to see such comparisons
2. I couldn't see disturbance data in this manuscript. However, a significant portion of the conclusion is about the effect of
disturbances. Thus, the conclusion should focus on the findings of the manuscript.
3. The width of the bars in Figure 3 is not to the standard

**Author’s Response:** Thank you for your valuable feedback; we have included the size of the Seqela Forest (197.6 ha) in the manuscript, revised the conclusion to focus on the findings, and corrected the width of the bars in Figure 3 to meet standard requirements.

**Reviewer #4:** Please correct the species names in the abstract. The objectives can be fine tuned to align with the results.

**Author’s Response:** Thank you for your valuable feedback. We have corrected the objectives in the revised manuscript to align more closely with the results. However, we would like to clarify that the species names in the abstract are correct as originally presented, except for ***Acacia abyssinica****,* which has been updated to ***Vachellia abyssinica*** as per the recent taxonomic classification.
